# Genome-wide identification of WRKY transcription factor family members in *Miscanthus sinensis* (*Miscanthus sinensis Anderss*)

**DOI:** 10.1038/s41598-024-55849-1

**Published:** 2024-03-06

**Authors:** Yongkang Yan, Zhanyou Yan, Guofang Zhao

**Affiliations:** 1 Faculty of Science, the University of Hong Kong, Hong Kong, China; 2 Shijiazhuang Tiedao University, Shijiazhuang, China; 3 Hebei Vocational University of Industry and Technology, Shijiazhuang, China

**Keywords:** *Miscanthus sinensis*, WRKY, Phylogenetic analysis, Biotic stress, Expression profiling, Molecular biology, Plant sciences

## Abstract

Miscanthus is an emerging sustainable bioenergy crop whose growing environment is subject to many abiotic and biological stresses. WRKY transcription factors play an important role in stress response and growth of biotic and abiotic. To clarify the distribution and expression of the WRKY genes in Miscanthus, it is necessary to classify and phylogenetically analyze the WRKY genes in Miscanthus. The v7.1 genome assembly of Miscanthus was analyzed by constructing an evolutionary tree. In Miscanthus, there are 179 WRKY genes were identified. The 179 *MsWRKY*s were classified into three groups with conserved gene structure and motif composition. The tissue expression profile of the WRKY genes showed that *MsWRKY* genes played an essential role in all growth stages of plants. At the early stage of plant development, the *MsWRKY* gene is mainly expressed in the rhizome of plants. In the middle stage, it is mainly expressed in the leaf. At the end stage, mainly in the stem. According to the results, it showed significant differences in the expression of the *MsWRKY* in different stages of *Miscanthus sinensis*. The results of the study contribute to a better understanding of the role of the *MsWRKY* gene in the growth and development of Miscanthus.

## Introduction

WRKY transcription factors (TFs) were widely distributed in plants, which were first discovered in sweet potato (Ipomoea batatas)^1^. With genome-wide analyses of different species, WRKY genes have been identified in more species, that included 66 WRKY genes in Arabidopsis^2^, 119 WRKY genes in maize^3^, 94 WRKY genes in sorghum^4^, 79 WRKY genes in potato^5^, 70 WRKY genes in chickpea^6^, and 61 WRKY genes in cucumber^7^. The WRKY protein contains a conserved WRKYGQK motif at its N-terminal and a 60-amino acid-long zinc finger motif at its C-terminus^8^. Zinc finger motifs can be classified as C2H2 or C2HC. WRKY proteins can be classified into three categories (I, II, III) based on the number of WRKY domains and the type of zinc finger motif^9^. Group I members have two WRKY domains and zinc fingers of type C2H2. Group II members have only one WRKY domain and one C2H2 zinc finger motif, and Group III members have one WRKY domain and one C2HC zinc finger. Group II can be further subdivided into five subgroups: IIa, IIb, IIc, IId, and IIe^10,11^.

Studies have shown that WRKY TFs play an important regulatory role in plant growth and development^12^. For example, *AtWRKY12* and *AtWRKY13* can exert important regulation on the flowering process of Arabidopsis^13^. The over-expression of *GsWRKY20* from *Glycine soja* in Arabidopsis also altered the plant's flowering process^14^. However, *OsWRKY11* in rice can control flowering time and plant height^15^. These depend on WRKY genes being expressed at different times in different parts of the plant. Similarly, the expression of these genes also affects various aspects of plants, such as hormone regulation and stress resistance^16^.

For example, overexpression of *OsWRKY45* in rice enhances disease resistance and drought resistance^17^. The *AtWRKY25* and *AtWRKY33* in Arabidopsis thaliana enhance salt tolerance^18^. Some *AcWRKY* TFs of kiwifruit were up-regulated under salt stress^19^. Considering the important role of WRKY TFs in plant growth and development, the study of these genes is very important for agricultural production. The overexpression of *TaWRKY2* in wheat enhanced the tolerance to drought stress and increased the yield^20^. *Vitis amurensis VaWRKY12* gene enhanced the cold tolerance of transgenic grape callus^21^.

Most studies have focused on the genes of annual grasses, but few studies have focused on the genes of perennial grasses. Miscanthus is a perennial grass that has historically been cultivated as a papermaking material and as an ornamental plant. In recent years, Miscanthus has played a role in the direction of ecological restoration and sustainable bioenergy crops^22^. Therefore, the study has great significance for the genetic improvement of Miscanthus. The improvements can increase productivity and ensure that the crops remain robust despite persistent biological and abiotic stresses. As an essential stress resistance gene, WRKY TFs have high research value. The availability of the whole genome assembly of *Miscanthus sinensis* enabled identifying *MsWRKY*. The identification and study of *MsWRKY* are helpful in understanding the mechanism of plant stress resistance. The genomes of at least four Miscanthus species (*M. sacchariflorus*, *M. sinensis*, *M. lutarioriparius*, and *M. floridulus*) have been sequenced. *M. sinensis* was used as the object of this study. M. sacchariflorus has strong stress resistance and large biomass. However, the growth cycle of the plant is too long for commercial biomass production. M. lutarioriparius and M. floridulus have a narrow distribution range and are only found in parts of East Asia. Their biomass yield is low, and the fiber content is too high. Therefore, they are not suitable for the bioenergy development. The function of the Miscanthus WRKY family has been identified and characterized by various methods.

## Material and methods

### Identification of WRKY family genes in Miscanthus

The genomic data for Miscanthus were obtained from Phytozome 13 (https://phytozome-next.jgi.doe.gov/). From these data, the putative *MsWRKY* genes can be identified. The database contains the amino acid sequences of Miscanthus WRKY proteins. The Joint Genome Institute (JGI) (https://phytozome.jgi.doe.gov/pz/portal.html#) and the WRKY domain ID were used to identify the potential WRKY (PF03106) proteins of Miscanthus. The data used was *Miscanthus sinensis* v7.1. To ensure the quality of the data, the CD-HIT suite (https://github.com/weizhongli/cdhit) and Simple Modular Architecture the Research Tool (SMART) (http://smart.embl-heidelberg.de/#) were used to process the resulting sequence^23^. The duplicate sequences and incomplete sequences were removed.

At the same time, the ExPASy proteomic server (http://web.expasy.org/protparam) was used to predict the physical and chemical properties of the proteins. Their isoelectric point (pI) and molecular weight (MW) were obtained.

### Chromosome mapping and classification and phylogenetic analysis of *MsWRKY* genes

The chromosomal locations of all identified *MsWRKY* genes were obtained from the Phytozome BioMart tool (https://phytozome.jgi.doe.gov/biomart/martview/). And the MG2C v2.1 (mg2c.iask.in/mg2c_v2.1/) was used for Chromosome mapping of *MsWRKY* genes.

In constructing a phylogenetic tree to classify *MsWRKY* genes, we need to use *Sorghum bicolor* WRKY amino acid sequences. The data were obtained from The Arabidopsis Information Resource (TAIR) (https://www.arabidopsis.org/). They will be used with our *MsWRKY* sequences. The MEGA v7.0 (https://www.megasoftware.net/) for constructing a phylogenetic tree used multiple sequence alignments with ClustalW to process *SbWRKY* and *MsWRKY* protein sequences. The Neighbor-Joining method and the p-distance model were used in the process, and the pairwise deletion and 1000 bootstrap replicates were selected^24^. Eventually, the phylogenetic tree of *SbWRKY* and *MsWRKY* sequences was obtained. Thus, the unknown *MsWRKY* genes can be divided into different groups and subgroups. By using the sequence alignment data and the phylogenetic tree, the putative Miscanthus WRKY orthologs in Arabidopsis can be identified^5^.

### Gene structure analysis and conserved motif distribution analysis of *MsWRKY* genes

The genomic sequence and coding sequence (CDS) of each *MsWRKY* gene can be used to predict the gene structure of the *MsWRKY* gene. The exon–intron structure of *MsWRKY*s was analyzed using TBtools^25^.

The Multiple Em for Motif Elicitation (MEME) (v.5.5.3; https://meme-suite.org/meme/tools/meme) used the parameters which are: maximum motif number: 20; site distributions: any number of repetitions; minimum and maximum width: 6 and 50, respectively to distinguish *MsWRKY* proteins with conserved motif.^26^.

### Gene ontology annotation and analysis of cis-acting elements of *MsWRKY* genes

For the gene ontology (GO) annotation analysis of the obtained *MsWRKY* proteins, the eggNOG-mapper 2.1.12 (http://eggnog-mapper.embl.de/)^27^ was used. Then, the TBtools was used to map and annotate the obtained data. Ultimately, the biological processes, molecular functions and cellular components of these proteins were obtained.

The online website PlantCARE (http://bioinformatics.psb.ugent.be/webtools/plantcare/html/) analyzed 2000 bp of the upstream region for all *MsWRKY* genes to analyze the cis-acting elements of *MsWRKY* genes. It provided the cis-acting elements of *MsWRKY* genes.

### Synteny analysis of *MsWRKY* genes

The Multiple Collinearity Scan toolkit (MCScanX) was used to examine the gene duplication events with the default parameters. The TBtools was the platform used to analyze the data. The Evalue of the blastP was 15. To explore the syntenic relationships of the WRKY genes obtained from Miscanthus and other selected species, syntenic analysis maps were constructed using MCScanX.

### Digital expression pattern analysis of *MsWRKY* genes

The TBtools analyzed the Miscanthus transcriptomic array data to make heatmaps of the *MsWRKY* expression profiles to survey *MsWRKY* expression profiles. In the meantime, the Miscanthus transcriptomic array data was obtained from the JGI database (https://phytozome-next.jgi.doe.gov/).

## Results

### Identification of WRKY family members in Miscanthus

To identify members of the *MsWRKY* family, the WRKY domain consensus sequence (PF03106) and the keyword WRKY were used to search in the database. The most complete genome assembly for Miscanthus (*Miscanthus sinensis* v7.1) in the JGI database was selected. In the *MsWRKY* family, a conserved domain exists as the basic criterion for inclusion of genes. Using the conserved domain called the WRKYGQK or WRKYGQK-like conserved domain, 203 genes were identified in the JGI database. In these genes, the duplicates and incomplete were removed by the multiple sequence alignments of MEGA 7.0. At the same time, we removed the severely incomplete gene, identified the location of WRKY domain, and kept some of the incomplete genes because they clearly belonged to the *MsWRKY* family. It was done using the SMART database. In the end, there are a total of 179 non-redundant *MsWRKY* sequences. The sequences and proteins of these genes are summarized in Table 1.

**Table 1:** Characteristics of the identified *MsWRKY* genes.

Gene name	Gene locus ID	Chromosome location	Gene start	Gene end	pI	MW	Conserved heptapeptide	Zinc finger type	Domain number	Group	Protein length (aa)
*MsWRKY01*	Misin01G001000.1	Chr01	256,399	260,899	5.96	42,019.13	WRKYGQK	C2H2	1	IIe	388
*MsWRKY02*	Misin01G047900.1	Chr01	7,447,384	7,449,297	9.28	45,084.5	WRKYGQK	C2H2	1	IId	423
*MsWRKY03*	Misin01G063100.1	Chr01	9,967,017	9,972,013	6.41	34,538.04	WRKYGQK	C2H2	1	IIc	331
*MsWRKY04*	Misin01G064300.1	Chr01	10,185,437	10,187,725	6.27	44,986.89	WRKYGQK	C2H2	2	I	420
*MsWRKY05*	Misin01G080100.1	Chr01	13,181,193	13,184,725	10.03	43,273.5	WRKYGQK	C2H2	1	IId	402
*MsWRKY06*	Misin01G144700.1	Chr01	27,617,080	27,617,952	9.51	11,896.32	WRKYGQK	C2H2	1	IId	108
*MsWRKY07*	Misin01G144800.1	Chr01	27,658,926	27,661,691	8.79	14,941.48	WKKYGQK	C2H2	1	IId	138
*MsWRKY08*	Misin01G263700.1	Chr01	84,350,262	84,352,266	6.35	47,584.95	WRKYGQK	C2H2	1	IIe	441
*MsWRKY09*	Misin01G315300.1	Chr01	105,230,385	105,233,381	6.67	29,695.24	WRKYGQK	C2H2	2	I	270
*MsWRKY10*	Misin01G341600.1	Chr01	112,898,915	112,899,930	10.23	18,701.01	WRKYGQK	C2	1	IId	179
*MsWRKY11*	Misin01G362700.1	Chr01	118,028,855	118,030,800	5.97	33,081.46	WRKYGQK	C2HC	1	III	319
*MsWRKY12*	Misin01G370500.1	Chr01	119,779,719	119,781,349	8.71	23,777.31	WRKYGEK	C2HC	1	III	219
*MsWRKY13*	Misin02G032200.1	Chr02	4,903,340	4,905,683	9.42	46,713.58	WRKYGQK	C2H2	1	IId	439
*MsWRKY14*	Misin02G061600.1	Chr02	10,345,970	10,348,174	6.33	45,153.2	WRKYGQK	C2H2	2	I	419
*MsWRKY15*	Misin02G062400.1	Chr02	10,531,789	10,536,585	8.28	36,115.94	WRKYGQK	C2H2	1	IIc	342
*MsWRKY16*	Misin02G074100.1	Chr02	12,502,555	12,505,918	10.03	43,237.35	WRKYGQK	C2H2	1	IId	403
*MsWRKY17*	Misin02G115600.1	Chr02	21,231,516	21,233,686	8.81	15,261.74	WKKYGQK	C2H2	1	IId	142
*MsWRKY18*	Misin02G115700.1	Chr02	21,277,852	21,278,769	9.3	17,306.11	WRKYGQK	C2H2	1	IId	161
*MsWRKY19*	Misin02G129800.1	Chr02	24,888,414	24,891,276	5.13	29,550.9	WRKYGQK	C2HC	1	III	275
*MsWRKY20*	Misin02G139500.1	Chr02	27,977,090	27,980,493	9.78	37,940.88	WRKYGQK	C2H2	1	IId	351
*MsWRKY21*	Misin02G258000.1	Chr02	82,777,287	82,779,248	6.21	51,394.67	WRKYGQK	C2H2	1	IIe	477
*MsWRKY22*	Misin02G303500.1	Chr02	98,992,567	99,008,454	7.04	51,566.58	WRKYGQK	C2H2	2	I	487
*MsWRKY23*	Misin02G345000.1	Chr02	109,902,400	109,904,305	5.39	32,934.03	WRKYGQK	C2HC	1	III	316
*MsWRKY24*	Misin02G360900.1	Chr02	113,665,512	113,666,881	8.5	23,918.39	WRKYGEK	C2HC	1	III	221
*MsWRKY25*	Misin03G030100.1	Chr03	7,315,277	7,319,424	5.91	40,003.58	WRKYGQK	C2H2	1	IIc	385
*MsWRKY26*	Misin03G090400.1	Chr03	26,714,328	26,724,781	6.53	65,823.66	WKIYHEK	C2H2	1	III	577
*MsWRKY27*	Misin03G145800.1	Chr03	58,356,338	58,358,329	5.9	34,210.05	WRKYGQK	C2HC	1	III	323
*MsWRKY28*	Misin03G145900.1	Chr03	58,463,020	58,464,851	5.15	32,889.18	WRKYGQK	C2HC	1	III	308
*MsWRKY29*	Misin03G167600.1	Chr03	67,670,898	67,672,930	7.5	34,390.44	WRKYGQK	C2H2	1	IIa	322
*MsWRKY30*	Misin03G167700.1	Chr03	67,713,031	67,714,003	5.67	27,360.52	WRKYGQK	C2H2	1	IIa	255
*MsWRKY31*	Misin03G309200.1	Chr03	99,305,845	99,312,404	6.22	65,765.89	WRKYGQK	C2H2	2	I	612
*MsWRKY32*	Misin03G348200.1	Chr03	105,876,921	105,879,425	6.52	34,512.02	WRKYGQK	C2HC	1	III	334
*MsWRKY33*	Misin04G012600.1	Chr04	2,811,844	2,816,422	5.71	40,011.36	WRKYGQK	C2H2	1	IIc	388
*MsWRKY34*	Misin04G103000.1	Chr04	28,871,792	28,881,760	6.64	180,043.9	WEKFGEK	C2H2	1	III	1620
*MsWRKY35*	Misin04G121800.1	Chr04	37,165,681	37,167,742	8.1	32,376.41	WRKYGQK	C2HC	1	III	310
*MsWRKY36*	Misin04G159500.1	Chr04	59,513,396	59,515,552	5.8	33,477.21	WRKYGQK	C2HC	1	III	314
*MsWRKY37*	Misin04G159600.1	Chr04	59,590,103	59,591,840	6.27	35,403.26	WRKYGQK	C2HC	1	III	333
*MsWRKY38*	Misin04G159700.1	Chr04	59,628,053	59,630,374	6.41	32,262.04	WRKYGQK	C2HC	1	III	300
*MsWRKY39*	Misin04G189100.1	Chr04	70,520,338	70,521,730	8.61	24,998.4	WRKYGQK	C2H2	1	IIa	232
*MsWRKY40*	Misin04G189300.1	Chr04	70,613,566	70,615,037	6.45	29,149.56	WSKYGQK	C2H2	1	IIa	272
*MsWRKY41*	Misin04G224400.1	Chr04	80,781,032	80,788,531	6.07	62,448.14	WRKYGQK	C2H2	2	I	584
*MsWRKY42*	Misin04G335900.1	Chr04	103,115,716	103,123,318	6.2	65,841.98	WRKYGQK	C2H2	2	I	613
*MsWRKY43*	Misin04G395900.1	Chr04	113,184,557	113,186,904	9.49	28,237.28	WRKYGQK	C2HC	1	III	266
*MsWRKY44*	Misin05G004000.1	Chr05	1,062,340	1,065,068	6.95	40,831.11	–––-	C2H2	0	IIb	398
*MsWRKY45*	Misin05G004600.1	Chr05	1,127,680	1,130,708	9.02	61,031.03	WRKYGQK	C2H2	1	IIb	586
*MsWRKY46*	Misin05G039300.1	Chr05	8,714,579	8,716,081	6.51	25,862.72	WRKYGKK	C2H2	1	IIc	247
*MsWRKY47*	Misin05G039400.1	Chr05	8,724,881	8,727,718	6.74	54,286.43	WRKYGQK	C2H2	1	IIb	529
*MsWRKY48*	Misin05G043000.1	Chr05	9,436,264	9,443,373	9.66	29,836.26	WRKYGQK	C2H2	1	IIc	282
*MsWRKY49*	Misin05G044100.1	Chr05	9,661,358	9,662,378	10.01	25,167.29	WRKYGQK	––	1	IIc	243
*MsWRKY50*	Misin05G133400.1	Chr05	33,763,626	33,768,855	6.92	58,669.54	WRKYGQK	C2H2	1	IIb	565
*MsWRKY51*	Misin05G179500.1	Chr05	61,537,333	61,538,346	5.02	23,472.75	WRK––	C2H2	1	IIc	218
*MsWRKY52*	Misin05G181000.1	Chr05	62,453,032	62,454,436	9.45	34,432.88	WRKYGQK	C2H2	1	IIb	334
*MsWRKY53*	Misin05G204000.1	Chr05	71,047,834	71,051,020	6.39	38,748.61	WRKYGQK	C2H2	1	IIc	358
*MsWRKY54*	Misin05G204800.1	Chr05	71,261,560	71,263,661	8.16	42,492.78	WRKYGQK	C2H2	1	IIe	399
*MsWRKY55*	Misin05G223600.1	Chr05	76,270,790	76,274,606	7.75	40,925.8	WRKYGQK	C2H2	1	IIc	392
*MsWRKY56*	Misin05G245300.1	Chr05	82,376,407	82,381,492	6.65	27,467.87	WRKYGQK	C2H2	1	IIc	257
*MsWRKY57*	Misin05G247500.1	Chr05	82,585,497	82,587,483	5.09	31,767.99	WRKYGQK	C2H2	1	IId	309
*MsWRKY58*	Misin05G257300.1	Chr05	84,706,704	84,708,239	8.87	23,279.05	WRKYGKK	C2H2	1	IIc	217
*MsWRKY59*	Misin05G266000.1	Chr05	87,076,200	87,078,166	4.84	33,807.07	WRKYGQK	C2H2	1	IIe	311
*MsWRKY60*	Misin05G314300.1	Chr05	97,084,382	97,087,219	6.07	58,493.75	WRKYGQK	C2H2	2	I	547
*MsWRKY61*	Misin05G318500.1	Chr05	97,853,542	97,855,451	6.13	28,871.07	WRKYGQK	C2HC	1	III	273
*MsWRKY62*	Misin05G318700.1	Chr05	97,894,342	97,897,352	5.55	35,408.38	WRKYGQK	C2HC	1	III	321
*MsWRKY63*	Misin05G318800.1	Chr05	97,904,621	97,907,196	5.88	39,828.44	WRKYGQK	C2HC	1	III	365
*MsWRKY64*	Misin05G318900.1	Chr05	97,921,736	97,926,790	5.56	29,563.32	WRKYGQK	C2HC	1	III	263
*MsWRKY65*	Misin05G319000.1	Chr05	97,947,986	97,954,617	5.15	24,871.51	–––-	C2HC	0	III	222
*MsWRKY66*	Misin05G341800.1	Chr05	102,614,804	102,620,037	6.21	37,871.09	WRKYGQK	C2H2	1	IId	360
*MsWRKY67*	Misin06G033600.1	Chr06	8,287,503	8,288,890	6.88	25,986.1	WRKYGKK	C2H2	1	IIc	248
*MsWRKY68*	Misin06G033700.1	Chr06	8,316,137	8,318,460	6.6	54,945.21	WRKYGQK	C2H2	1	IIb	535
*MsWRKY69*	Misin06G035300.1	Chr06	8,827,872	8,837,074	9.84	30,409.98	WRKYGQK	C2H2	1	IIc	288
*MsWRKY70*	Misin06G118100.1	Chr06	32,022,401	32,028,104	6.68	58,207.58	WRKYGQK	C2H2	1	IIb	559
*MsWRKY71*	Misin06G173600.1	Chr06	64,362,468	64,364,136	5.41	25,009.44	WRKYGKK	C2H2	1	IIc	230
*MsWRKY72*	Misin06G176500.1	Chr06	65,837,433	65,838,824	9.46	34,595.22	WRKYGQK	C2H2	1	IIb	330
*MsWRKY73*	Misin06G194000.1	Chr06	71,485,939	71,489,452	6.64	37,778.45	WRKYGQK	C2H2	1	IIe	352
*MsWRKY74*	Misin06G201700.1	Chr06	74,689,039	74,691,538	6.65	35,420.7	WRKYGQK	C2HC	1	III	341
*MsWRKY75*	Misin06G223300.1	Chr06	81,494,458	81,497,861	6.95	40,981.74	WRKYGQK	C2H2	1	IIc	394
*MsWRKY76*	Misin06G257000.1	Chr06	89,646,615	89,650,205	6.29	27,463.97	WRKYGQK	C2H2	1	IIc	256
*MsWRKY77*	Misin06G263000.1	Chr06	91,159,450	91,161,480	4.82	33,231.57	WRKYGQK	C2H2	1	IIe	308
*MsWRKY78*	Misin06G304500.1	Chr06	98,736,612	98,740,191	5.98	26,953.02	WRKYGQK	C2HC	1	III	238
*MsWRKY79*	Misin06G304600.1	Chr06	98,786,343	98,796,970	5.61	29,373.08	WRKYGQK	C2HC	1	III	262
*MsWRKY80*	Misin06G304700.1	Chr06	98,801,362	98,804,326	7.08	39,644.48	WRKYGQK	C2HC	1	III	365
*MsWRKY81*	Misin06G304800.1	Chr06	98,822,340	98,825,116	6.14	33,304.99	WRKYGQK	C2HC	1	III	308
*MsWRKY82*	Misin06G304900.1	Chr06	98,833,287	98,834,794	5.98	29,436.72	WRKYGQK	C2HC	1	III	276
*MsWRKY83*	Misin06G308400.1	Chr06	99,650,347	99,653,285	6.65	56,227.46	WRKYGQK	C2H2	2	I	524
*MsWRKY84*	Misin06G319700.1	Chr06	101,544,841	101,551,366	7.72	35,540.77	WRKYGQK	C2H2	1	IId	337
*MsWRKY85*	Misin07G063600.1	Chr07	11,867,503	11,869,447	8.81	38,782.96	WRKYGQK	C2H2	1	IIa	361
*MsWRKY86*	Misin07G113800.1	Chr07	22,968,339	22,970,872	5.77	40,000.76	WRKYGQK	C2H2	1	IIe	375
*MsWRKY87*	Misin07G139300.1	Chr07	29,364,149	29,365,802	9.23	11,245.87	WRKYGQK	C2H2	2	I	98
*MsWRKY88*	Misin07G156800.1	Chr07	33,883,167	33,890,997	6.45	62,499.9	WRKYGQK	C2H2	2	I	577
*MsWRKY89*	Misin07G221700.1	Chr07	48,363,328	48,367,783	6.18	73,536.66	WRKYGQK	C2H2	2	I	680
*MsWRKY90*	Misin07G320900.1	Chr07	80,414,542	80,415,531	6.49	35,113.11	WRKYGQK	C2HC	1	III	329
*MsWRKY91*	Misin07G336100.1	Chr07	97,182,907	97,186,054	9.53	32,283.89	WRKYGQK	C2H2	1	IId	304
*MsWRKY92*	Misin07G427800.1	Chr07	133,666,880	133,670,859	8.74	26,212.78	WRKYGQK	C2H2	1	IIc	237
*MsWRKY93*	Misin07G453800.1	Chr07	139,859,854	139,863,939	5.97	50,932.84	WRKYGQK	C2H2	1	IIe	487
*MsWRKY94*	Misin07G454800.1	Chr07	140,141,540	140,145,937	5.74	50,614.47	WRKYGQK	C2H2	1	IIe	487
*MsWRKY95*	Misin07G514500.1	Chr07	153,014,613	153,017,365	5.33	60,875.02	WRKYGQK	C2H2	1	IIb	575
*MsWRKY96*	Misin08G064100.1	Chr08	13,397,841	13,399,660	8.81	38,153.22	WRKYGQK	C2H2	1	IIa	357
*MsWRKY97*	Misin08G113400.1	Chr08	31,140,937	31,143,013	5.6	39,155.69	WRKYGQK	C2H2	1	IIe	368
*MsWRKY98*	Misin08G130600.1	Chr08	40,498,773	40,500,342	9.46	32,546.22	WRKYGQK	C2H2	1	IId	305
*MsWRKY99*	Misin08G223600.1	Chr08	74,637,099	74,641,364	8.47	26,138.66	WRKYGQK	C2H2	1	IIc	236
*MsWRKY100*	Misin08G295700.1	Chr08	89,997,647	90,000,423	5.41	64,800.38	WRKYGQK	C2H2	1	IIb	604
*MsWRKY101*	Misin09G094400.1	Chr09	31,825,786	31,831,560	6.09	142,969.7	WRKYGQK	C2H2	1	III	1269
*MsWRKY102*	Misin09G124800.1	Chr09	48,969,562	48,973,277	9.32	23,326.47	WRKYGQK	C2H2	1	IIc	212
*MsWRKY103*	Misin09G168300.1	Chr09	69,478,619	69,480,158	5.16	35,435.27	WRKYGEK	C2HC	1	III	315
*MsWRKY104*	Misin09G168400.1	Chr09	69,516,272	69,517,802	5.23	35,384.37	CRKYGEK	C2HC	1	III	315
*MsWRKY105*	Misin10G075100.1	Chr10	22,664,223	22,667,110	7.58	68,606.04	WRKYGR-	C2HC	1	III	602
*MsWRKY106*	Misin10G075200.1	Chr10	22,667,111	22,669,574	11.79	21,954.27	–––-	C2	0	III	197
*MsWRKY107*	Misin10G090500.1	Chr10	28,806,465	28,809,996	9.46	23,366.54	WRKYGQK	C2H2	1	IIc	211
*MsWRKY108*	Misin10G147700.1	Chr10	57,752,929	57,755,316	9.09	45,494.27	WNKYSQK	C2HC	1	III	402
*MsWRKY109*	Misin10G168900.1	Chr10	64,290,186	64,291,933	8.31	28,207.55	WRKYGEK	C2HC	1	III	251
*MsWRKY110*	Misin11G033500.1	Chr11	21,369,493	21,371,062	9.69	31,121.11	WRKYGQK	C2H2	1	IId	290
*MsWRKY111*	Misin11G109800.1	Chr11	50,960,040	50,966,140	6.99	76,641.46	WRKYGQK	C2H2	2	I	722
*MsWRKY112*	Misin11G161900.1	Chr11	61,907,380	61,911,291	8.87	26,425.98	WRKYGQK	C2H2	1	IIc	241
*MsWRKY113*	Misin11G172000.1	Chr11	63,970,651	63,972,274	10.11	32,836.5	WRKYGQK	C2H2	1	IId	311
*MsWRKY114*	Misin11G177600.1	Chr11	64,766,776	64,770,389	5.42	51,673.75	WRKYGQK	C2H2	1	IIe	490
*MsWRKY115*	Misin12G114700.1	Chr12	53,371,560	53,377,084	6.71	76,479.3	WRKYGQK	C2H2	2	I	721
*MsWRKY116*	Misin12G168300.1	Chr12	64,849,948	64,854,472	8.58	26,693.31	WRKYGQK	C2H2	1	IIc	246
*MsWRKY117*	Misin12G221100.1	Chr12	75,273,661	75,277,569	5.4	51,312.26	WRKYGQK	C2H2	1	IIe	490
*MsWRKY118*	Misin12G224200.1	Chr12	75,983,746	75,985,597	10.12	33,577.23	WRKYGQK	C2H2	1	IId	320
*MsWRKY119*	Misin13G053300.1	Chr13	15,284,932	15,287,835	9.19	23,715.16	WRKYGQK	C2H2	2	I	214
*MsWRKY120*	Misin13G053800.1	Chr13	15,379,156	15,382,102	8.1	24,999.43	WRKYGQK	C2H2	2	I	225
*MsWRKY121*	Misin13G065100.1	Chr13	19,010,992	19,011,900	9.97	31,219.41	WRKYGQK	C2H2	1	IId	302
*MsWRKY122*	Misin13G077200.1	Chr13	26,514,539	26,521,340	6.14	61,765.12	WRKYGQK	C2H2	2	I	568
*MsWRKY123*	Misin14G000600.1	Chr14	88,281	90,014	8.39	20,741.62	WRKYGQK	C2H2	2	I	187
*MsWRKY124*	Misin14G044100.1	Chr14	10,368,810	10,372,904	9.05	25,295.99	WRKYGEK	C2HC	1	III	225
*MsWRKY125*	Misin14G044300.1	Chr14	10,410,602	10,412,653	6.45	30,496.32	WRKYGQK	C2HC	1	III	269
*MsWRKY126*	Misin14G044400.1	Chr14	10,415,330	10,420,545	9.23	22,949.89	WRKYGQK	C2	1	III	197
*MsWRKY127*	Misin14G044700.1	Chr14	10,486,916	10,488,031	5.53	30,704.25	WRKYGQK	C2HC	1	III	271
*MsWRKY128*	Misin14G044900.1	Chr14	10,550,794	10,554,423	5.75	32,763.68	WRKYGQK	C2HC	1	III	316
*MsWRKY129*	Misin14G045000.1	Chr14	10,578,622	10,579,241	8.84	15,262.13	WRKYGQK	––	1	III	146
*MsWRKY130*	Misin14G045100.1	Chr14	10,615,079	10,617,387	5.35	39,100.04	WRKYGQK	C2HC	1	III	369
*MsWRKY131*	Misin14G055800.1	Chr14	13,292,467	13,294,425	5.81	33,108.79	WRKYGQK	C2HC	1	III	311
*MsWRKY132*	Misin14G094800.1	Chr14	36,988,970	36,997,185	7.26	51,967.19	WRKYGQK	C2H2	2	I	489
*MsWRKY133*	Misin14G145800.1	Chr14	53,975,422	53,978,412	10.11	39,116.59	WRKYGQK	C2H2	1	IId	367
*MsWRKY134*	Misin15G021700.1	Chr15	5,345,780	5,348,381	6.01	37,525.59	WRKYGQK	C2HC	1	III	354
*MsWRKY135*	Misin15G022300.1	Chr15	5,508,198	5,510,603	5.95	34,408.46	WRKYGQK	C2HC	1	III	301
*MsWRKY136*	Misin15G022400.1	Chr15	5,519,610	5,521,171	6.41	30,314.18	WRKYGQK	C2HC	1	III	269
*MsWRKY137*	Misin15G022600.1	Chr15	5,554,241	5,557,091	9.24	25,266.01	RRKYGEK	C2HC	1	III	224
*MsWRKY138*	Misin15G022700.1	Chr15	5,568,564	5,569,944	6.24	36,949.21	WRKYGEK	C2HC	1	III	349
*MsWRKY139*	Misin15G059000.1	Chr15	14,509,029	14,511,457	5.86	34,045.56	WRKYGQK	C2HC	1	III	321
*MsWRKY140*	Misin15G117000.1	Chr15	44,938,827	44,944,840	7.26	52,373.63	WRKYGQK	C2H2	2	I	494
*MsWRKY141*	Misin15G165100.1	Chr15	61,759,600	61,762,535	10.06	39,406.88	WRKYGQK	C2H2	1	IId	369
*MsWRKY142*	Misin15G207300.1	Chr15	72,991,988	72,993,980	5.68	33,773.54	WRKYGQK	C2HC	1	III	318
*MsWRKY143*	Misin16G028000.1	Chr16	6,831,166	6,841,544	6.1	42,064.15	WRKYGQK	C2H2	1	IIe	390
*MsWRKY144*	Misin16G048900.1	Chr16	10,992,577	10,999,877	6.07	55,955.5	WRKYGQK	C2H2	1	IIb	539
*MsWRKY145*	Misin16G081700.1	Chr16	22,116,722	22,118,179	8.38	19,270.16	WRKYGKK	C2H2	1	IIc	178
*MsWRKY146*	Misin16G101800.1	Chr16	37,889,291	37,892,295	6.76	44,475.77	WRKYGQK	C2HC	1	III	415
*MsWRKY147*	Misin16G105500.1	Chr16	40,038,943	40,041,203	8.11	52,702.08	WRKYGQK	C2H2	2	I	498
*MsWRKY148*	Misin16G172200.1	Chr16	63,685,681	63,688,656	6.24	59,899.74	WRKYGQK	C2H2	2	I	567
*MsWRKY149*	Misin16G174600.1	Chr16	64,449,520	64,453,297	9.67	16,747.88	WRKYGEK	––	1	III	149
*MsWRKY150*	Misin16G174700.1	Chr16	64,489,809	64,491,209	4.8	33,581.52	WRKYGQK	C2HC	1	III	305
*MsWRKY151*	Misin16G203800.1	Chr16	69,942,927	69,944,332	9.25	25,824.25	WRKYGQK	C2H2	1	IIc	245
*MsWRKY152*	Misin16G214500.1	Chr16	71,981,919	71,983,740	6.51	22,679.88	WRKYGKK	C2H2	1	IIc	220
*MsWRKY153*	Misin16G230900.1	Chr16	74,460,403	74,463,496	6.14	45,216.03	WRKYGQK	C2H2	1	IIc	427
*MsWRKY154*	Misin16G238000.1	Chr16	75,949,728	75,952,344	6.47	55,813.4	WRKYGQK	C2H2	1	IIb	533
*MsWRKY155*	Misin16G240400.1	Chr16	76,369,804	76,371,283	5.64	31,057.52	WRKYGQK	C2HC	1	III	292
*MsWRKY156*	Misin16G248100.1	Chr16	78,361,013	78,362,627	7.59	40,882.18	WRKYGQK	C2H2	1	IIe	383
*MsWRKY157*	Misin17G038700.1	Chr17	10,631,690	10,636,844	6.08	55,291.53	WRKYGQK	C2H2	1	IIb	532
*MsWRKY158*	Misin17G083500.1	Chr17	25,376,889	25,385,898	6.96	21,980.17	WRKYGKK	C2H2	1	IIc	204
*MsWRKY159*	Misin17G109300.1	Chr17	38,677,661	38,679,975	6.87	53,141.5	WRKYGQK	C2H2	2	I	507
*MsWRKY160*	Misin17G113800.1	Chr17	41,268,683	41,271,638	6.99	44,427.78	WRKYGQK	C2HC	1	III	414
*MsWRKY161*	Misin17G171600.1	Chr17	64,424,660	64,428,034	6.08	21,848.34	WTKYGEK	C2HC	1	III	191
*MsWRKY162*	Misin17G174100.1	Chr17	65,394,933	65,397,741	6.65	60,054.88	WRKYGQK	C2H2	2	I	563
*MsWRKY163*	Misin17G209900.1	Chr17	73,211,242	73,212,749	9.15	24,778.25	WRKYGQK	C2H2	1	IIc	234
*MsWRKY164*	Misin17G216300.1	Chr17	74,457,742	74,459,617	6.2	22,606.83	WRKYGKK	C2H2	1	IIc	218
*MsWRKY165*	Misin17G243200.1	Chr17	79,583,903	79,586,980	6.37	43,647.54	WRKYGQK	C2H2	1	IIc	414
*MsWRKY166*	Misin17G243900.1	Chr17	79,818,715	79,821,649	6.82	61,955.37	WRKYGQK	C2H2	1	IIb	589
*MsWRKY167*	Misin17G248100.1	Chr17	80,589,893	80,591,407	5.8	31,233.78	WRKYGQK	C2HC	1	III	294
*MsWRKY168*	Misin17G257600.1	Chr17	82,409,895	82,413,771	5.06	35,515.91	WRKYGQK	C2H2	1	IIc	336
*MsWRKY169*	Misin17G258100.1	Chr17	82,719,255	82,721,075	7.6	32,985.94	WRKYGQK	C2H2	1	IIe	314
*MsWRKY170*	Misin18G036700.1	Chr18	8,281,538	8,287,821	8.86	67,058.03	WRKYGQK	C2H2	1	IIb	638
*MsWRKY171*	Misin18G048700.1	Chr18	10,206,417	10,210,611	5.82	41,183.59	WRKYGQK	C2HC	1	III	385
*MsWRKY172*	Misin18G150100.1	Chr18	48,766,642	48,768,157	8.32	35,201.51	WRKYGQK	C2H2	1	IIe	321
*MsWRKY173*	Misin18G152000.1	Chr18	50,380,536	50,382,557	5.18	60,818.16	WRKYGEK	C2H2	1	IIe	569
*MsWRKY174*	Misin18G207000.1	Chr18	69,577,536	69,579,342	9.29	44,127.96	WRKYGQK	C2H2	1	IIa	411
*MsWRKY175*	Misin19G033600.1	Chr19	7,916,396	7,933,365	8.04	65,369.63	WRKYGQK	C2H2	1	IIb	624
*MsWRKY176*	Misin19G046600.1	Chr19	10,576,130	10,580,248	6.01	39,605.9	WRKYGQK	C2HC	1	III	371
*MsWRKY177*	Misin19G148800.1	Chr19	49,190,343	49,191,582	7.58	38,894.29	WRKYGQK	C2H2	1	IIe	359
*MsWRKY178*	Misin19G148900.1	Chr19	49,254,920	49,256,188	7.99	40,336.02	WRKYGQK	C2H2	1	IIe	377
*MsWRKY179*	Misin19G200400.1	Chr19	69,378,305	69,380,812	9.55	36,897.55	WRKYGQK	C2H2	1	IIa	343

These *MsWRKY*s have been named from *MsWRKY01* to *MsWRKY179* according to their distribution on the chromosome. The starting point was the upper arm of chromosome 1, moving down to the lower arm (Table 1). The properties of this set of *MsWRKY* proteins were investigated. The *MsWRKY* proteins ranged from 98 to 1620 amino acids. Their average length was 368 amino acids. Their predicted MW and pI values ranged from 11,246 to 180,044 and 4.8 to 11.79, respectively.

### Chromosome mapping and classification and phylogenetic analysis of *MsWRKY* genes

**Figure 1:**
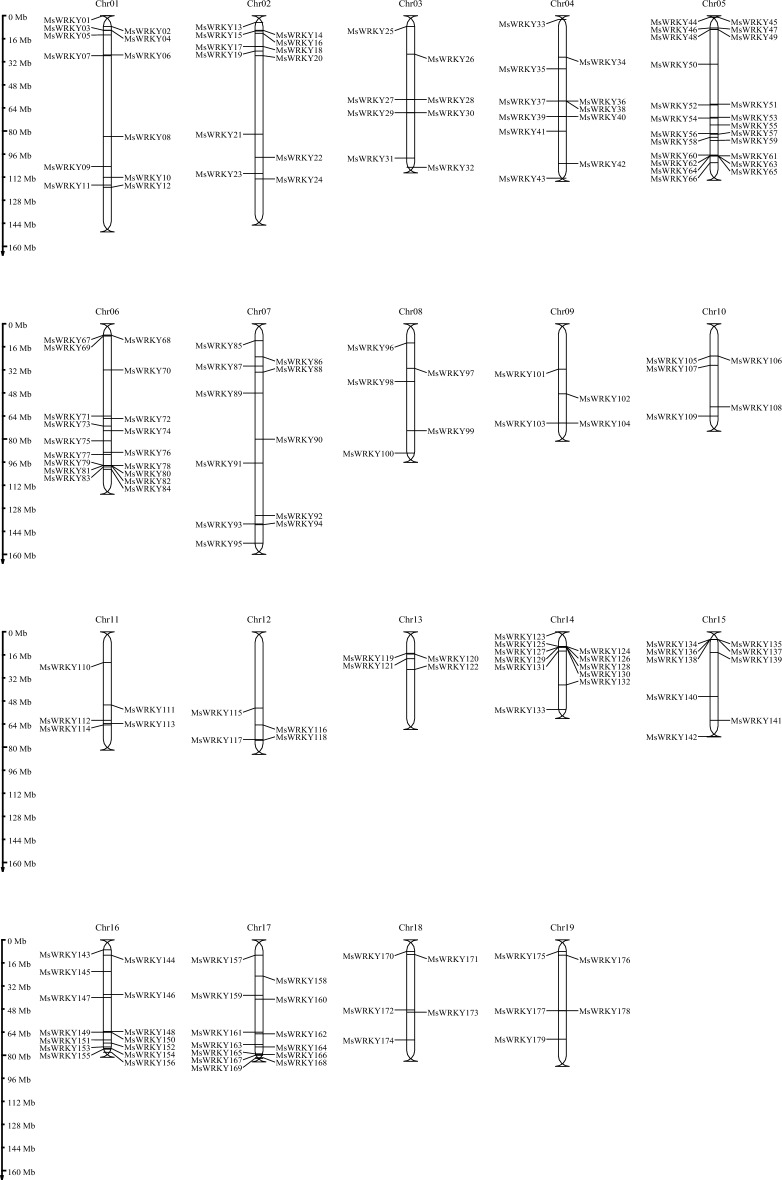
Distribution of 179 *Ms*WRKY genes on Miscanthus chromosomes.

The locations of the 179 *MsWRKY* genes were determined by MG2C v2.1 (Fig. 1). *MsWRKY* genes were distributed on all 19 Miscanthus chromosomes (Chr). Chr01-Chr19 was the chromosome (Chr) number indicating the names and positions of the *MsWRKY*. Chr 5 had the highest number of *MsWRKY*s, with 23, representing 12.9% of the total gene family. It was followed by 18 genes on Chr 6, 14 on Chr 16, and 13 on Chr 17. Chromosomes 9, 12, and 13 had only four *MsWRKY*s each, the fewest.

An unrooted phylogenetic tree to study the evolution of *MsWRKY* family members was constructed by the multiple sequence alignment and neighbor-joining method in MEGA7.0. The data was full-length protein sequences of 40 *SbWRKY*s and 179 *MsWRKY*s. *SbWRKY*s are used as the basis for grouping. They were from *Sorghum bicolor (L.) moench*, a Poaceae plant that is similar to *Miscanthus sinensis*.

**Figure 2:**
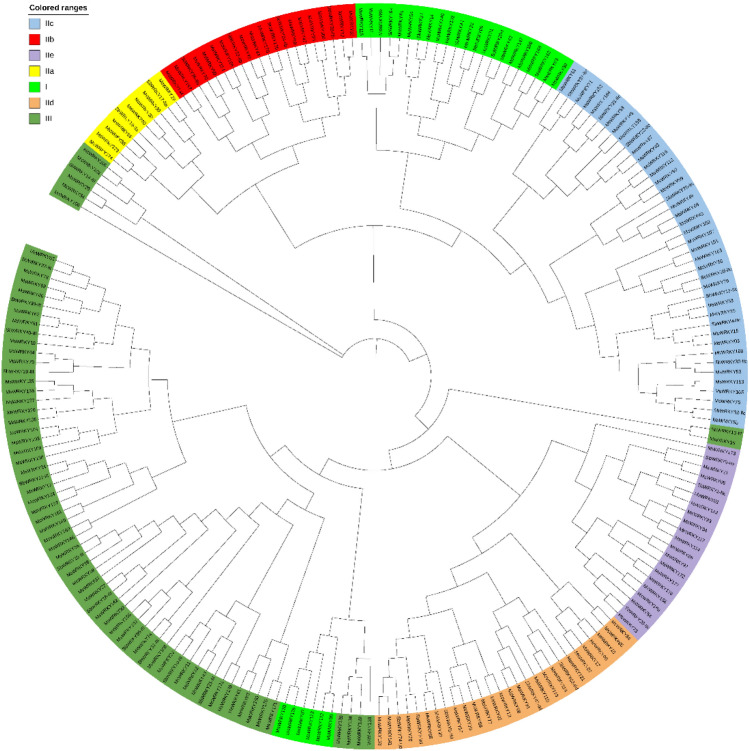
Phylogenetic tree of WRKY members in Miscanthus and Sorghum. All *MsWRKY*s genes were further divided into subgroups I, II, and III, and group II was further divided into subgroups IIa, IIb, IIc, IId, and IIe.

179 *MsWRKY*s could be divided into three major groups (I, II, and III) according to the constructed phylogenetic tree (Fig. 2). Of the 24 *MsWRKY*s in group I, all of them had two WRKYGQK motifs and 23 of them have two C2H2-type zinc finger motifs (C-X3-4-C-X22-23-H-X1-H), corresponding to two complete WRKY domains. Although the protein encoded by *MsWRKY09* had only one zinc finger motif, it belonged to group I on the phylogenetic tree.

Group II had a total of 97 protein sequences and was the largest group, accounting for 54.2% of all putative *MsWRKY*s. Similar reports can also be found in sorghum, cucumber and chickpeas. Most of these proteins had one WRKY domain and the C2H2-type zinc finger motif (C-X4-5-C-X23-H-X1-H). This group was further divided into five subgroups, IIa, IIb, IIc, IId, and IIe, with 8, 16, 32, 21, and 20 members, respectively. Fifty-eight proteins belong to Group III. The proteins in this group had one WRKY domain and the C2HC-type zinc finger motif (C-X7-C-X23-H-X1-C)^28^. In summary, the classification of *MsWRKY*s indicated the diversity of these proteins. An extremely wide range of functions could be performed by these proteins.

### Gene structure analysis and conserved motif distribution analysis of *MsWRKY* genes

**Figure 3:**
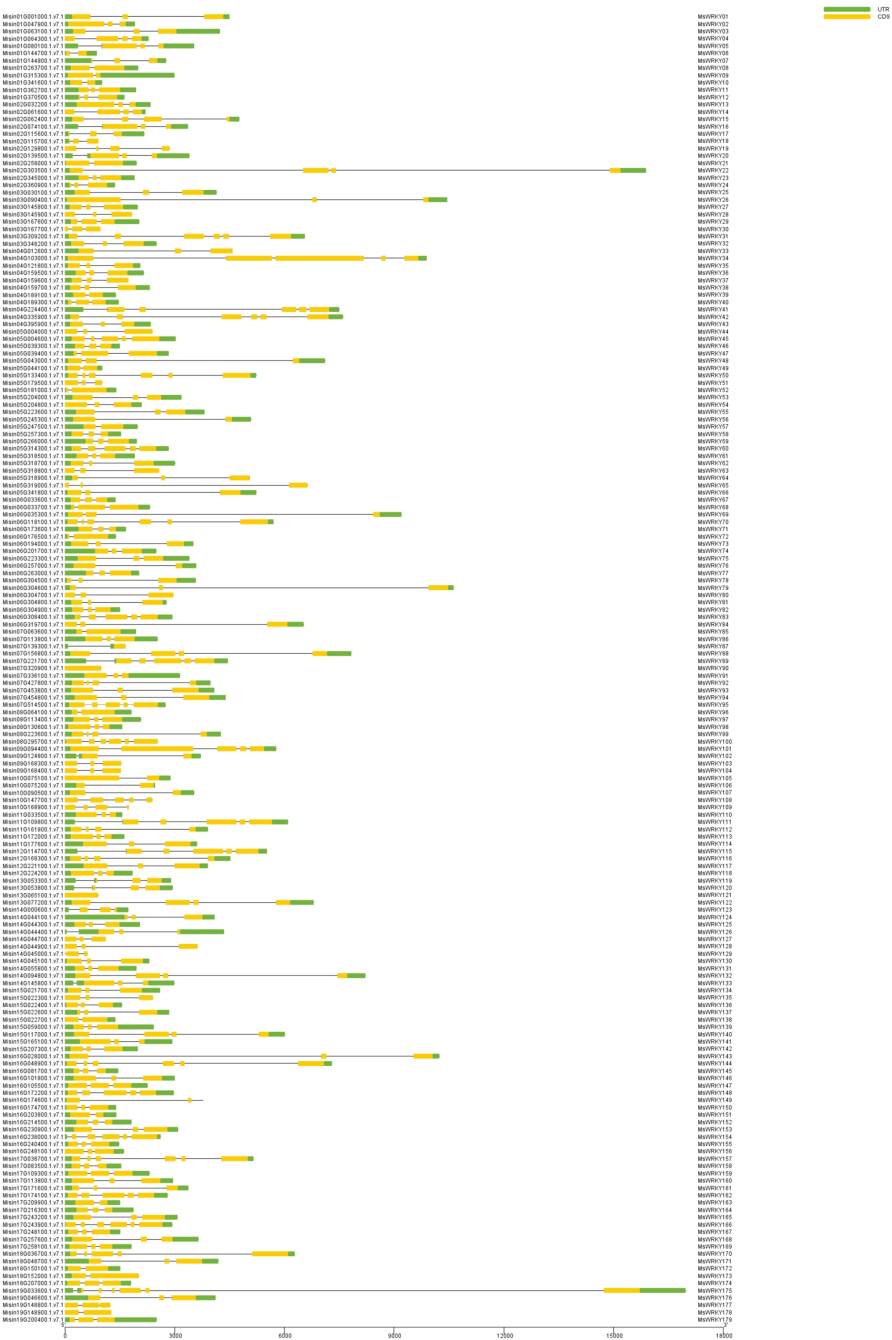
Exon–intron structures of *MsWRKY* genes. Exon–intron structures of *MsWRKY* genes were obtained after analysed with TBtools for gene structure. Green bars indicate upstream and downstream UTRs, yellow bars indicate coding sequences (CDS), and black lines indicate introns in the gene diagrams.

The exon–intron structures of *MsWRKY* family members can illustrate the evolution of *MsWRKY* family members. The introns number of *MsWRKY* genes ranged from zero to five, while their size varied. The data showed that genes within the same group had certain similarities in the exon–intron distribution patterns. These results suggest that the *MsWRKY* genes had important structural diversity. It represented the functional diversity among closely related members of *MsWRKY*s (Fig. 3).

**Figure 4:**
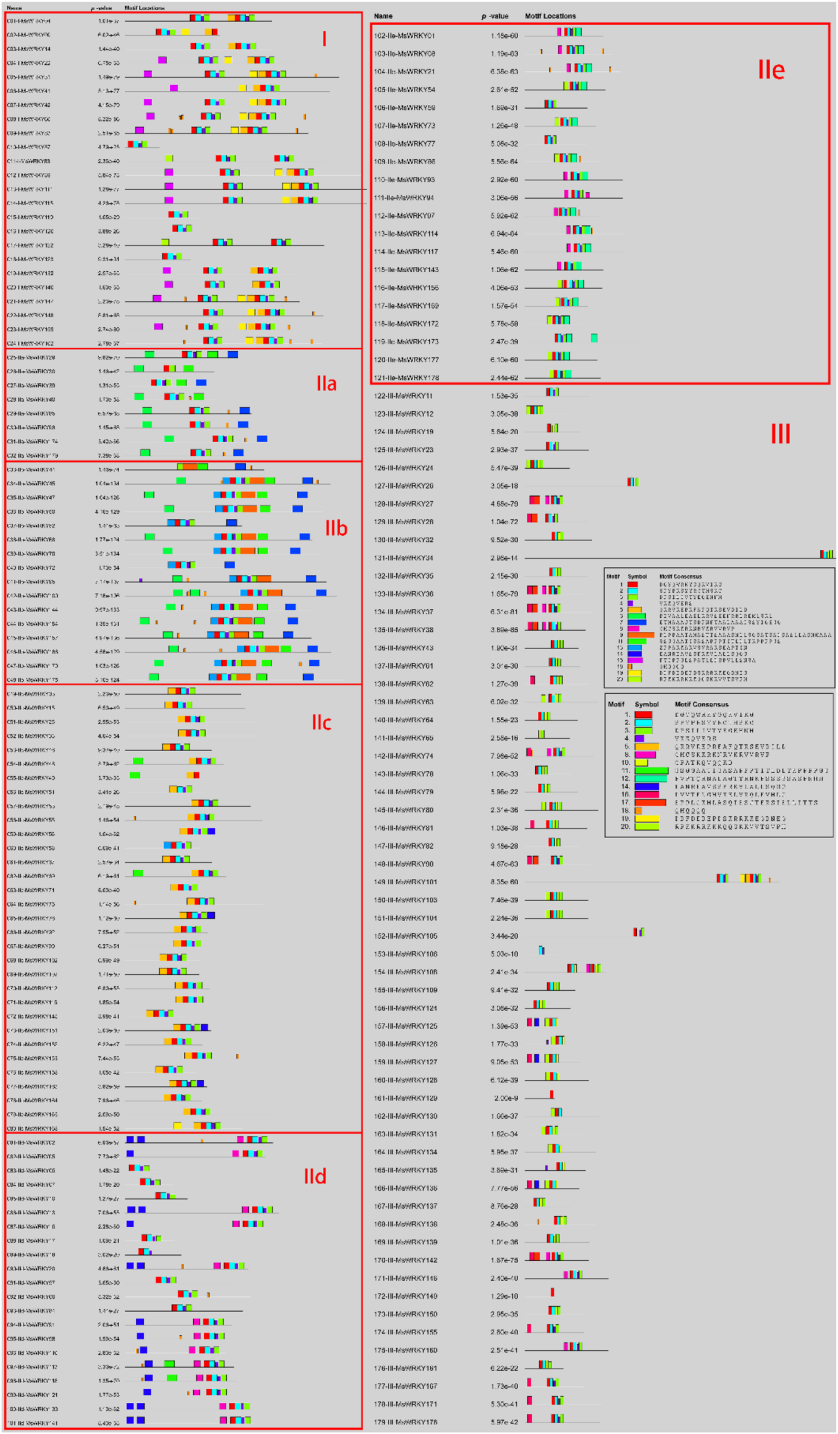
Motif analysis of *MsWRKY*s.

MEME (version 5.5.3), used to analyze the conserved motifs of all *MsWRKY* protein sequences, identified 20 distinct conserved motifs. The distribution of 20 conserved motifs identified by MEME in the different groups of *MsWRKY*s is shown in Fig. 4. Motifs 1 is the WRKY domain. Similar Motif structures can be found between the genes in the same group or subgroup through the results. Motifs 1, 2, 3, and 4 are found in almost all genes. Motifs 15 and 19 were unique to group I. Motifs 9 and 13 were unique to group IIb. Motifs 12 were unique to group IIe. Motifs 10, 16, and 18 were unique to group III. Some of the motifs shared by different groups. Motif 5 was shared by groups I and IIc, and motif 6 and 7 were shared by groups IIa and IIb.

### Gene ontology annotation and analysis of cis-acting elements of *MsWRKY* genes

**Figure 5:**
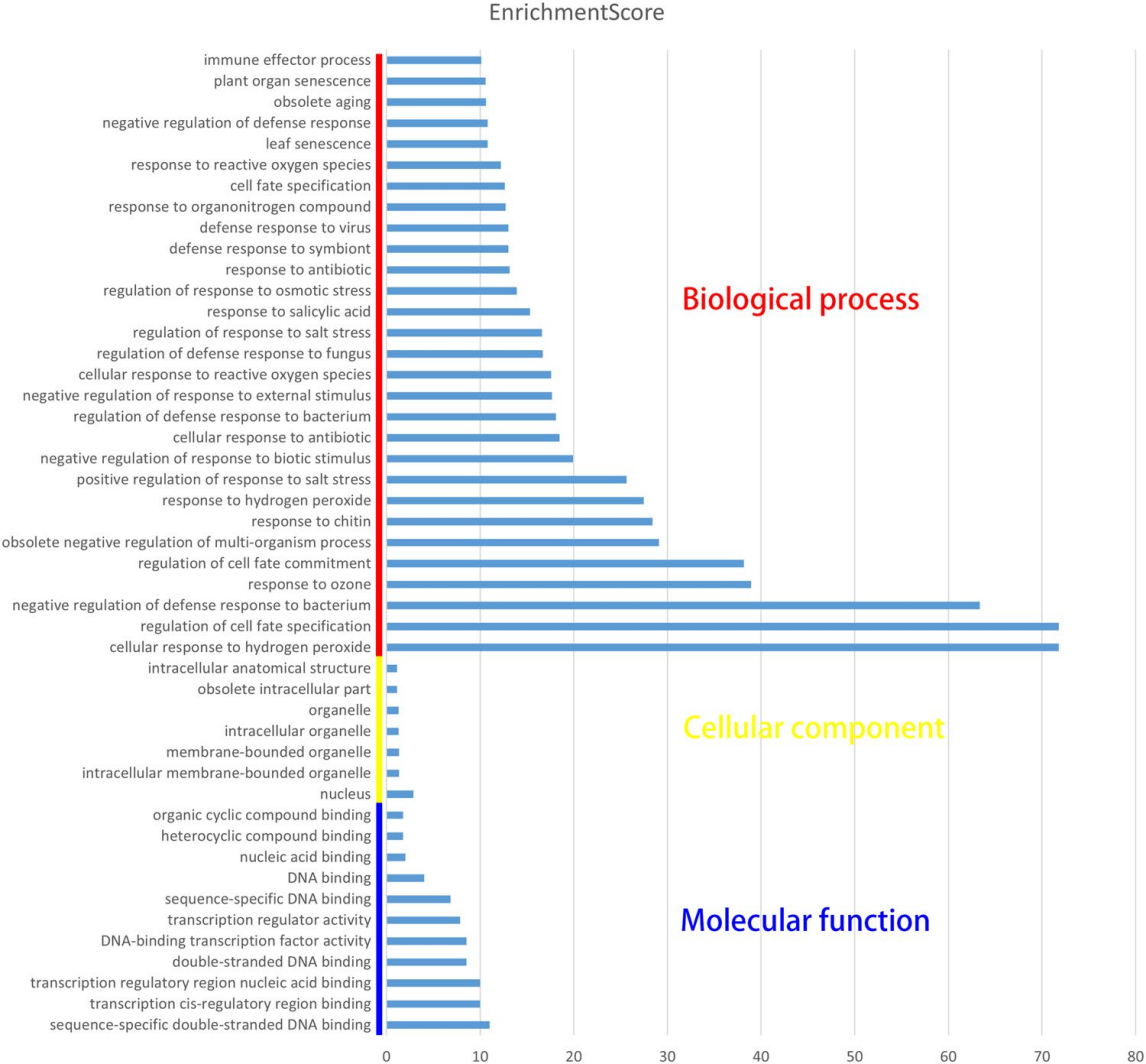
Gene ontology analysis of identified *MsWRKY*s.

The Blast2GO analyzed the Gene ontology (GO) annotations of 179 *MsWRKY* proteins. The *MsWRKY* target genes can be categorized into three main categories according to different functional groups. The biological processes, molecular functions, and cellular components together make up the Gene ontology (GO) annotations. Through the enrichment analysis, the involvement of *MsWRKY* in biological processes, molecular functions, and cellular components is in Fig. 5. Most *MsWRKY*s are involved in regulating cellular processes, biosynthetic processes, and different metabolic processes. Further analysis showed that most *MsWRKY*s were involved in the plant's stress to external adversity. Many *MsWRKY*s have been linked to bacterial infections and environmental stress^29^. The molecular functions of *MsWRKY*s are mainly a variety of DNA-binding and gene expression regulation. The cellular component of this protein family is mainly organelle and intracellular organelle. Most of the *MsWRKY* proteins are located in the cell nucleus.

Cis-acting elements are essential sequences in regulating gene expression by transcription factors. An online tool PlantCARE was used to analyze all *MsWRKY* cis-acting elements and extracted 2000 bp promoter regions upstream of all *MsWRKY* genes. The result shows that every *MsWRKY* have many cis-acting elements.

Firstly, many transcription-related cis-acting elements can be found including TATA-box, CAAT-box, A-box, HD-Zip, and W-box. The stress-responsive elements formed an important part in the cis-acting elements. This suggests that *MsWRKY* plays an important role in plants' resistance to external stress. These cis-acting elements include MBS (Anti-drought stress), LTR (anti-low temperature stress), and WUN-motif (wound-responsive elements). In addition, many of the cis-acting elements were regulated by phytohormones. ABA regulates the ABA-responsive element (ABRE). Methyl jasmonate (MeJA) responsive element (TGACG-motif and CGTCA-motif) is regulated by jasmonate phytohormones^30^. Finally, there are auxin-responsive elements (AuxRR-core and TGA-element), salicylic acid-responsive elements (TCA-element), etc. Finally, there are many light-responsive elements and other regulatory elements. At least one stress-responsive element is on all of the *MsWRKY* genes and reflects the potential functional variation of the *MsWRKY* gene.

### Synteny analysis of *MsWRKY* genes

**Figure 6:**
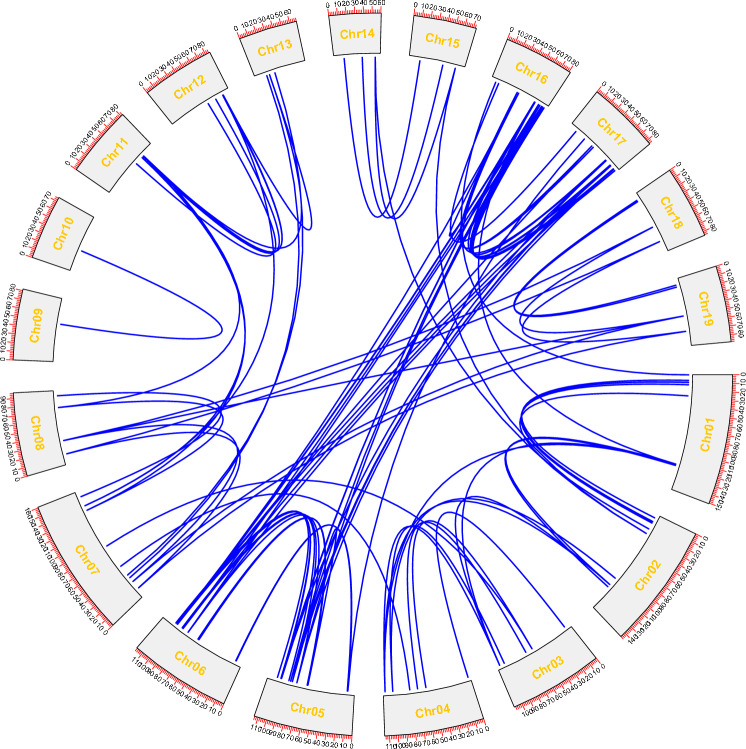
Schematic representations for the interchromosomal relationships of *MsWRKY*s. Blue lines show duplicated WRKY gene pairs in the Miscanthus genome.

The segmental duplication events occurring in the Miscanthus WRKY family were investigated by conducting a synteny analysis of the *MsWRKY* genes using BLASTP and MCScanX in TBtools. As shown in Fig. 6, 19 segmental duplication events involving 53 WRKY genes were observed. Tandem duplication events, which were defined by a chromosomal region within 200 kb containing two or more genes, were widely identified for Miscanthus WRKY genes. A very large tandem duplication event was observed in the chromosome 14. These results suggested that some *MsWRKY*s were possibly generated by segmental duplication events and that the evolution of *MsWRKY* genes may have been driven, at least in part, by segmental duplication events.

**Figure 7:**
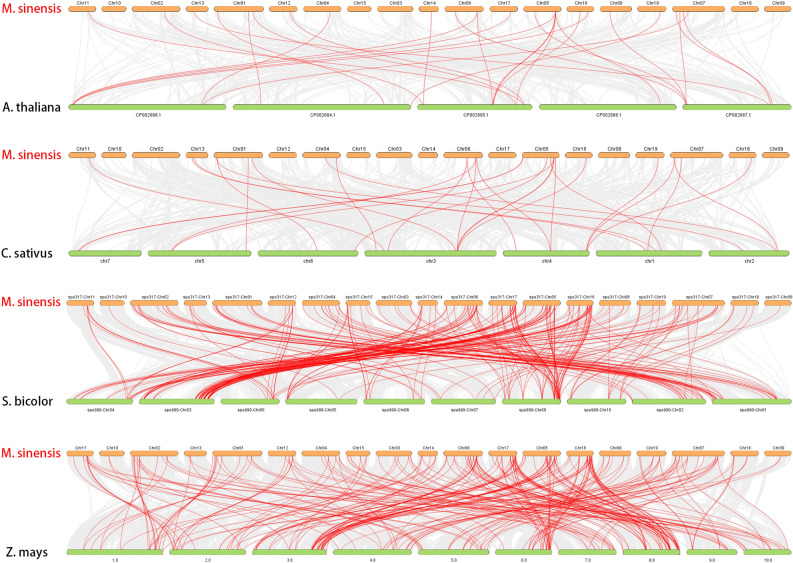
Synteny analysis of WRKYs between Miscanthus and other plant species. The collinear blocks are marked by gray lines, while the collinear gene pairs with WRKY genes are highlighted in the red lines. ‘*M. sinensis*’, ‘*A. thaliana*’, ‘*C. sativus*’, ‘*S. bicolor*’ and ‘*Z. mays*’ indicate *Miscanthus sinensis*, *Arabidopsis thaliana*, *Cucumis sativus*, *Sorghum bicolor*, and *Zea mays*, respectively.

The phylogenetic mechanisms of the Miscanthus WRKY family were further explored by constructing comparative syntenic maps of cucumber associated with four representative species, including two dicots (Arabidopsis and cucumber) and two monocots (sorghum and maize) (Fig. 7). 249, 231, 28, and 27 pairs of genes showed syntenic relationships between the other four species: cucumber, Arabidopsis, sorghum and maize, respectively. A total of 249 WRKY collinear gene pairs between Miscanthus and maize were identified, followed by Miscanthus and sorghum (231), Miscanthus and cucumber (28), and Miscanthus and Arabidopsis (27). Both Miscanthus and maize belong to the Poaceae family, and more than 75.4% of the *MsWRKY* genes showed a syntenic relationship with WRKYs in maize. But some of *MsWRKY* genes were associated with more than one syntenic gene pair, indicating that WRKY genes in Poaceae family have gone through multiple rounds of duplication events. This may be the reason why monocotyledonous plants have far more WRKY genes than dicotyledonous plants. Importantly, collinear *MsWRKY09/11/60/64/83/85/112/179* genes pairs were observed between Miscanthus and all of the other four species, suggesting that these orthologous pairs may have formed before the divergence of dicotyledonous and monocotyledonous plants.

### Digital expression analysis of *MsWRKY* genes at different seasons and in different tissues

**Figure 8:**
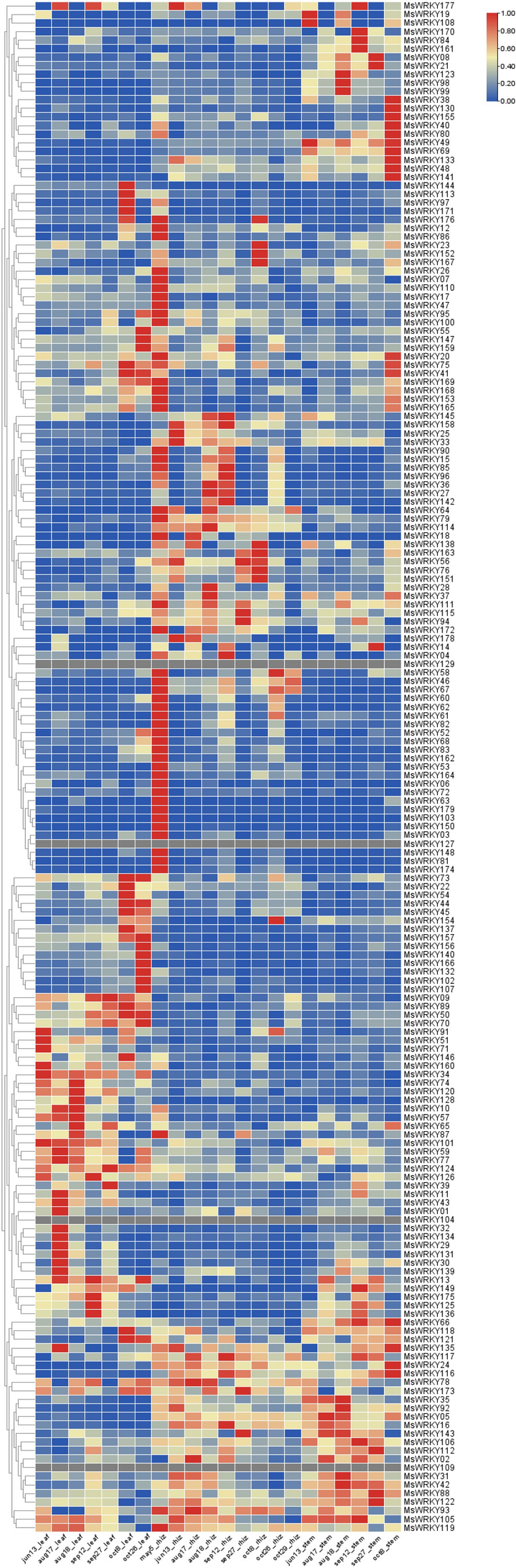
Heatmaps of *MsWRKY* gene expression. *MsWRKY* expression levels in different tissues and at different seasons.

The study of the temporal and spatial expression profiles of *MsWRKY* genes used the microarray data provided by the JGI database and presented the results as heatmaps by TBtools. the microarray datasets used gene expression data for *M. sinensis* and included a total of 22 samples. The samples were taken from leaf (7), rhizome (9) and stem (6). The samples were collected from plants at different times of the year and reflected the expression of the *MsWRKY* gene at different stages of plant growth. 175 of the 179 genes showed differential expression in plants. Most *MsWRKY* genes are highly expressed in rhizome. The expression patterns of *MsWRKY* in different growth and development stages were also analyzed. Firstly, the *MsWRKY* gene is first expressed in rhizome in large quantities during plant growth. Then, some *MsWRKY* genes were heavily expressed in the leaf. Eventually, some *MsWRKY* genes are over-expression in the stem when plants wither. The results showed that these genes may be involved in stress response at sensitive developmental stages to improve plant tolerance (Fig. 8).

By analyzing the gene expression heat map and cis-acting elements together, it can be found that genes activated in different periods have different characteristics. In the early stage of plant development, the expression of the *MsWRKY* gene is mostly controlled by plant hormones and light regulatory elements. In the middle stage of plant development, the *MsWRKY* gene expressed in leaves is more regulated by infection and injury. At the end of plant development, *MsWRKY* genes expressed were mostly regulated by ABA and jasmonic acid, and some were stressed by environmental conditions such as drought. This suggests that the *MsWRKY* gene plays an important role in the growth of the perennial plant *Miscanthus sinensis*.

## Discussion

WRKY transcription factors (TFs) are widely distributed in the plant kingdom and play a crucial role in stress tolerance. The WRKY gene family comprises 66 genes in Arabidopsis, 119 in maize, 94 in sorghum, 79 in potatoes, 70 in chickpeas, and 61 in cucumbers. By analyzing the genomic assembly of *Miscanthus sinensis*, 179 WRKY genes were identified. As the *Miscanthus sinensis* is a paleotetraploid^31^, the amount of the WRKY genes is much higher than that of normal plants, but this has not received much attention in previous studies. The *MsWRKY* gene is distributed across all 19 chromosomes of Miscanthus, and we have identified *MsWRKY92*, located on chromosome 7, as a gene that promotes flowering^32^. Conserved WRKY domains bind to the W-box motif in the promoter of WRKY target genes, which is the most important feature of the WRKY family^33,34^. A phylogenetic analysis of all the obtained *MsWRKY* genes has been performed. We performed a phylogenetic analysis of all the obtained *MsWRKY* genes and classified them into groups I, II, and III based on the number of WRKY domains and the type of zinc finger motif. group II is further subdivided into five subgroups: IIa, IIb, IIc, IId, and IIe. Group I had 24 *MsWRKY* genes, group II had 97, and group III had 58. In group II, group IIc had the most *MsWRKY* genes, with 32. The proportions of these genes are similar to those found in other plants^35–37^.

Most *MsWRKY* genes have a very conserved WRKYGQK motif. However, other similar sequences have been found in many genes. (*MsWRKY07 MsWRKY12 MsWRKY17 MsWRKY24 MsWRKY26 MsWRKY34 MsWRKY40 MsWRKY46 MsWRKY51 MsWRKY58 MsWRKY67 MsWRKY71 MsWRKY103 MsWRKY104 MsWRKY105 MsWRKY108 MsWRKY109 MsWRKY124 MsWRKY137 MsWRKY138 MsWRKY145 MsWRKY149 MsWRKY152 MsWRKY158 MsWRKY161 MsWRKY164 MsWRKY173*) Furthermore, there are some genes that are clearly WRKY genes but are missing this sequence (*MsWRKY44 MsWRKY65 MsWRKY106*). These differences can seriously affect the ability of *MsWRKY* proteins to bind to W-box elements, which in turn affects the biological function of these proteins. Similar heptapeptide motif variations have been found in other plants, such as sorghum^4^. In soybeans, for example, two WRKY genes with WRKYGKK motif do not bind to normal W-box elements and do not work^33^. Therefore, further studies are necessary to confirm the biological function of these WRKY motif aberrant genes.

Studying the exon–intron structure of *MsWRKY* genes can reveal population-specific patterns. Similar *MsWRKY* genes on the evolutionary tree tend to have similar exon–intron patterns. The number of introns in the *MsWRKY* gene ranges from 0 to 5, and some *MsWRKY* genes do not contain introns, indicating that some WRKY genes have experienced intron loss events^38^. Intron-free genes have been found in other organisms. There are three main mechanisms by which intron-free genes are produced: reverse transcription (the integration of sequences from RNA into the genome), duplication of existing intron-free genes, and horizontal gene transfer^39^. Differences in the intron size of *MsWRKY* genes may result from gene duplication, inversion, and/or fusion events^40^. In conclusion, the diverse exon–intron structure of *MsWRKY* genes reflects the evolutionary diversity of the *MsWRKY* gene family.

Motif structural studies of the *MsWRKY* gene family reveal both structural conservatism and diversity. Motifs 1, 2, 3, and 4 correspond to WRKY domains and zinc finger domains, and they are found in most *MsWRKY* genes. Although most motifs' functions are unclear, their distribution also has certain rules. Motifs 15 and 19 were unique to group I. Motifs 9 and 13 were unique to group IIb. Motifs 12 were unique to group IIe. Motifs 10, 16, and 18 were unique to group III. Some of the motifs shared by different groups included motif 5, shared by groups I and IIc, and motifs 6 and 7, shared by groups IIa and IIb. Motif 8 is a nuclear localization signal (NLS), mainly in groups IId, IIe, and III^41^. In conclusion, motif analysis clearly demonstrates the structural differences of genes in different groups in *MsWRKY*. These motifs may reflect that these genes participate in specific biological processes and play similar biological functions.

Studying the cis-acting elements of *MsWRKY* can obtain more information about the gene expression of the *MsWRKY* gene family. Firstly, many transcription-related cis-acting elements^42^ including TATA-box, CAAT-box, A-box and HD-Zip are essential for gene expression, and most are involved in constructing transcription complexes. In addition, there is also a batch of cis-acting elements regulated by phytohormones. ABA regulates the ABA-responsive element (ABRE). Methyl jasmonate (MeJA) responsive element (TGACG-motif and CGTCA-motif) is regulated by jasmonate phytohormones. In addition, there are auxin-responsive elements (AuxRR-core and TGA-element), salicylic acid-responsive elements (TCA-element), and so on. A variety of biological and abiotic stresses also regulate these genes. These cis-acting elements include MBS (Anti-drought stress), LTR (anti-low temperature stress), and WUN-motif (wound-responsive elements). At the same time, W-box was also found in the promoter region of many *MsWRKY* genes. This suggests that there is also mutual regulation between WRKY genes^43,44^. Studies on cis-acting elements of *MsWRKY* reflect the diversity of the *MsWRKY* gene in gene expression regulation.

Existing transcriptome data of *Miscanthus sinensis* can be used to analyze the expression of *MsWRKY* gene in different stages of Miscanthus development and expression patterns in different tissues. Future studies should investigate the function of the *MsWRKY* gene by studying homologous genes in other model plants (such as Arabidopsis) and related plants (other grasses). Future research should focus on the effect of *MsWRKY* on plant growth and development and against adverse winter environments. Additionally, exploring the role of the *MsWRKY* gene in plant stress response would be valuable. Our research results provide a foundation for future studies in this field.

## Conclusion

In this study, 179 WRKY genes were identified from *Miscanthus sinensis*. The identification, chromosome mapping, classification, phylogenetic analysis, gene structure analysis, conserved motif distribution analysis, gene ontology annotation, analysis of cis-acting elements, and digital expression pattern analysis have been performed. Through digital expression pattern analysis, the specific expression of the *MsWRKY* gene in different developmental stages and different parts of plants was found. At the same time, some *MsWRKY* genes may play an important role in plant stress resistance. In conclusion, this study is conducive to further research on the important functions of the WRKY gene in response to abiotic and biological stresses.

### Supplementary Information

Supplementary Information.

## Data Availability

All custom scripts used for parsing and analyzing transposable elements, gene families, and gene expression, as described in Supplementary Notes, are available at JGI and NCBI database [https://data.jgi.doe.gov/refine-download/phytozome?organism=Msinensis&expanded=497&_gl=1*13m4cmx*_ga*MTQwODM0NDIwMy4xNjk4MDQwMzEw*_ga_YBLMHYR3C2*MTY5ODA0MDMwOS4xLjEuMTY5ODA0MDgwMS4wLjAuMA..] [https://www.ncbi.nlm.nih.gov/bioproject/PRJNA575573] [https://www.ncbi.nlm.nih.gov/bioproject/PRJNA346689].
