# Supplementary material for: Genome-wide identification of WRKY transcription factor family members in Miscanthus sinensis (Miscanthus sinensis Anderss)
Source: Sci Rep. 2024 Mar 6;14:5522. doi: 10.1038/s41598-024-55849-1 (PMC10918066; doi:10.1038/s41598-024-55849-1)
Supplement: Supplementary file 1 — Supplementary Information. [file 41598_2024_55849_MOESM1_ESM.zip › Genome-wide Identification of WRKY Transcription Factor Family Members in Miscanthus sinensis (Miscanthus sinensis Anderss).docx]

**Genome-wide Identification of WRKY Transcription Factor Family Members in *Miscanthus sinensis* (*Miscanthus sinensis Anderss*)**

Yongkang Yan^[[1]](#footnote-1),*^, Zhanyou Yan^2^, Guofang Zhao^3^

^1^ Faculty of Science, the University of Hong Kong, Hong Kong, China

^2^ Shijiazhuang Tiedao University, Shijiazhuang, China

^3^ Hebei Vocational University of Industry and Technology, Shijiazhuang, China

**Abstract:** Miscanthus is an emerging sustainable bioenergy crop whose growing environment has many abiotic and biological stresses. WRKY transcription factors play an important role in stress response and growth of biotic and abiotic. To clarify the distribution and expression of the WRKY genes in Miscanthus, it is necessary to classify and phylogenetically analyze the WRKY genes in Miscanthus. The assembly of the genome v7.1 of Miscanthus was analyzed by establishing an evolutionary tree. In Miscanthus, there are 179 WRKY genes have been identified. The 179 *MsWRKY*s were divided into three groups with conserved gene structure and motif composition. The tissue expression profile of the WRKY genes showed that *MsWRKY* genes played an essential role in all growth stages of plants. In the early stage of plant development, the *MsWRKY* gene is mainly expressed in the rhizome of plants. During the middle stage, it is mainly expressed in the leaf. At the end stage, mainly in the stem. It was shown that biological or abiotic stresses, light, and plant hormones regulate *MsWRKY*. The study's results contribute to further understanding of the role of *MsWRKY* genes in Miscanthus's abiotic and biological stress resistance.

**Keywords:** *Miscanthus sinensis*, WRKY, phylogenetic analysis, Biotic stress, Expression profiling

**Introduction**

WRKY transcription factors (TFs) exist widely in plants. It was initially found in sweet potato (Ipomoea batatas) [1]. With genome-wide analyses of different species, WRKY genes have been identified in more species. This includes 66 WRKY genes in Arabidopsis [2], 119 WRKY genes in maize [3], 94 WRKY genes in sorghum [4], 79 WRKY genes in potatoes [5], 70 WRKY genes in chickpeas [6], and 61 WRKY genes in cucumbers [7]. The WRKY protein contains a conserved WRKYGQK motif at its N-terminal and a 60-amino acid-long zinc finger motif at its C-terminal [8]. Zinc finger motifs can be classified as C2H2 or C2HC. WRKY proteins can be classified into three categories (I, II, III) based on the number of WRKY domains and the type of zinc finger motif [9]. Group I members have two WRKY domains and zinc fingers of type C2H2. Group II members have only one WRKY domain and one C2H2 zinc finger motif, and Group III members have one WRKY domain and one C2HC zinc finger. Group II can be further divided into five subgroups: IIa, IIb, IIc, IId, and IIe [10, 11].

Studies have shown that WRKY TFs are involved in response to biological stress [12], such as many abiotic stresses and physiological processes in plant growth and reproduction [13]. WRKY TFs play an important role in regulating biological processes and gene expression in plants. The first is the role of WRKY TFs on plant stress tolerance. For example, overexpression of *OsWRKY45* in rice enhances disease resistance and drought resistance [14]. The *AtWRKY25* and *AtWRKY33* in Arabidopsis thaliana enhance its salt tolerance [15]. Some *AcWRKY* TFs of kiwifruit were up-regulated under salt stress [16]. In addition, WRKY TFs are also regulated by plant hormones. For example, WRKY TF expression is induced by hormone treatments such as ethylene, jasmonic acid, salicylic acid, gibberellin, and abscisic acid [17]. This suggests that WRKY TFs are also involved in the cascade of plant hormone signaling. In addition, some WRKY TFs are also negative regulators of gene expression. Given the important role of WRKY TFs in plant stress resistance, studying these genes is very important for agricultural production. The overexpression of *TaWRKY2* in wheat enhanced the tolerance to drought stress and increased the yield [18]. *Vitis amurensis* *VaWRKY12* gene enhanced cold tolerance of transgenic grape callus [19].

Most of the studies are aimed at the genes of annual grasses, but only a few studies are on the genes of perennial grasses. Miscanthus is a perennial weed historically cultivated as a papermaking material and an ornamental plant. In recent years, Miscanthus has played a role in the direction of ecological restoration and sustainable bioenergy crops [20]. Therefore, the study has a great significance for the genetic improvement of Miscanthus. These improvements can increase productivity and ensure that these crops remain robust in the face of persistent biological and abiotic stresses. As an essential stress resistance gene, WRKY TFs have high research value. The availability of the whole genome assembly of *Miscanthus sinensis* made identifying *MsWRKY* possible. The identification and study of *MsWRKY* are helpful in understanding the mechanism of plant stress resistance. At least four species of Miscanthus (*M. sacchariflorus*, *M. sinensis*, *M. lutarioriparius*, and *M. floridulus*) have had their genomes sequenced. *M. sinensis* was used as the object of this study. M. sacchariflorus has strong stress resistance and large biomass. But the plant's growth cycle is too long for commercial biomass production. M. lutarioriparius and M. floridulus have a narrow distribution range and are only found in parts of East Asia. Their biomass yield is low, the fiber content is too high. So they are not suitable for the development of bioenergy. The function of the Miscanthus WRKY family was identified and characterized by various methods.

**Material and methods**

**Identification of WRKY family genes in Miscanthus**

The genomic data for Miscanthus was obtained from Phytozome 13 (https://phytozome-next.jgi.doe.gov/). From this data, the putative *MsWRKY* genes can be identified. The database contains the amino acid sequences of Miscanthus WRKY proteins. The Genome Institute (JGI) (https://phytozome.jgi.doe.gov/pz/portal.html#) and the WRKY domain ID were used to identify the potential WRKY (PF03106) proteins of Miscanthus. The data used was *Miscanthus sinensis* v7.1. To ensure the quality of the data, the CD-HIT suite (https://github.com/weizhongli/cdhit) and Simple Modular Architecture the Research Tool (SMART) (http://smart.embl-heidelberg.de/#) were used to process the resulting sequence [21]. The duplicate sequences and incomplete sequences have been removed.

At the same time, the ExPASy proteomic server (http://web.expasy.org/protparam) was used to predict the physical and chemical properties of the proteins. Their isoelectric point (pI) and molecular weight (MW) were obtained.

**Chromosome mapping and classification and phylogenetic analysis of *MsWRKY* genes**

The chromosomal locations of all identified *MsWRKY* genes were obtained from the Phytozome BioMart tool (https://phytozome.jgi.doe.gov/biomart/martview/). And the MG2C v2.1 (mg2c.iask.in/mg2c_v2.1/) was used to make Chromosome mapping of *MsWRKY* genes.

In constructing a phylogenetic tree to classify *MsWRKY* genes, we need to use *Sorghum bicolor* WRKY amino acid sequences. The data came from the Arabidopsis Information Resource (TAIR) (https://www.arabidopsis.org/). They will be used with our *MsWRKY* sequences. The MEGA v7.0 (https://www.megasoftware.net/) for constructing a phylogenetic tree used multiple sequence alignments with ClustalW to process *SbWRKY* and *MsWRKY* protein sequences. In the process, the Neighbor-Joining method and the p-distance model were used, and the pairwise deletion and 1,000 bootstrap replicates were selected [22]. Eventually, the phylogenetic tree of *SbWRKY* and *MsWRKY* sequences was obtained. Thus, the unknown *MsWRKY* genes can be divided into different groups and subgroups. By using sequence alignment data and the phylogenetic tree, the putative Miscanthus WRKY orthologs in Arabidopsis can be identified [23].

**Gene structure analysis and conserved motif distribution analysis of *MsWRKY* genes**

The genomic sequence and coding sequence (CDS) of each *MsWRKY* gene can be used to predict the gene structure of the *MsWRKY* gene. The exon-intron structure of *MsWRKY*s was analyzed by TBtools [24].

The Multiple Em for Motif Elicitation (MEME) (v.5.5.3; https://meme-suite.org/meme/tools/meme) used the parameters which are: maximum motif number: 20; site distributions: any number of repetitions; minimum and maximum width: 6 and 50, respectively to distinguish *MsWRKY* proteins of conserved motif. [25].

**Gene ontology annotation and analysis of cis-acting elements of *MsWRKY* genes**

The gene ontology (GO) annotation analysis of the obtained *MsWRKY* proteins used the eggNOG-mapper 2.1.12 (<http://eggnog-mapper.embl.de/>) [26]. Then, the TBtools was used to map and annotate the obtained data. Ultimately, these analyses obtained these proteins' biological processes, molecular functions, and cellular components.

The online website PlantCARE (http://bioinformatics.psb.ugent.be/webtools/plantcare/html/) analyzed 2,000 bp of the upstream region for all *MsWRKY* genes to analyze the cis-acting elements of *MsWRKY* genes. Thus, the cis-acting elements of *MsWRKY* genes are obtained.

**Synteny analysis of *MsWRKY* genes**

The Multiple Collinearity Scan toolkit (MCScanX) was used to examine the gene duplication events, with the default parameters. The TBtools was the platform to analysis the data. The Evalue of the blastP is 15. To explore the syntenic relationships of the WRKY genes obtained from Miscanthus and other selected species, syntenic analysis maps were constructed using MCScanX.

**Digital expression pattern analysis of *MsWRKY* genes**

The TBtools analyzed the Miscanthus transcriptomic array data to make heatmaps of the *MsWRKY* expression profiles to survey *MsWRKY* expression profiles. In the meantime, the Miscanthus transcriptomic array data was obtained from the JGI database (https://phytozome-next.jgi.doe.gov/).

**Results**

**Identification of WRKY family members in Miscanthus**

To identify *MsWRKY* family members, the WRKY domain consensus sequence (PF03106) and the keyword WRKY were used to search in the database. The most complete genome assembly for Miscanthus (*Miscanthus sinensis* v7.1) in the JGI database was selected. In the *MsWRKY* family, a conserved domain exists as the basic criterion for the inclusion of genes. Using the conserved domain called the WRKYGQK or WRKYGQK-like conserved domain, 203 genes were identified in the JGI database. In these genes, the duplicates and incomplete were removed by the multiple sequence alignments of MEGA 7.0. At the same time, we removed the severely incomplete gene, identified the WRKY domain's location, and kept some of the incomplete genes because they clearly belonged to the *MsWRKY* family. The SMART database was used to do this. In the end, there are a total of 179 non-redundant *MsWRKY* sequences. The sequences and protein sequences of these genes are summarized in Table 1.

These *MsWRKY*s have been named from *MsWRKY01* to *MsWRKY179* according to their distribution on the chromosome. The starting point is the upper arm of chromosome 1, moving down to the lower arm (Table 1). The properties of this set of *MsWRKY* proteins were investigated. *MsWRKY* proteins ranged from 98 to 1,620 amino acids. Their average length is 368 amino acids. And their predicted MW and pI values ranged from 11246 to 180044 and from 4.8 to 11.79, respectively.

Table 1. Characteristics of the identified *MsWRKY* genes

| Gene Name | Gene Locus ID | Chromosome Location | Gene Start | Gene End | pI | MW | Conserved Heptapeptide | Zinc Finger Type | Domain Number | Group | Protein Length (aa) |
| --- | --- | --- | --- | --- | --- | --- | --- | --- | --- | --- | --- |
| *MsWRKY01* | Misin01G001000.1 | Chr01 | 256399 | 260899 | 5.96 | 42019.13 | WRKYGQK | C2H2 | 1 | IIe | 388 |
| *MsWRKY02* | Misin01G047900.1 | Chr01 | 7447384 | 7449297 | 9.28 | 45084.5 | WRKYGQK | C2H2 | 1 | IId | 423 |
| *MsWRKY03* | Misin01G063100.1 | Chr01 | 9967017 | 9972013 | 6.41 | 34538.04 | WRKYGQK | C2H2 | 1 | IIc | 331 |
| *MsWRKY04* | Misin01G064300.1 | Chr01 | 10185437 | 10187725 | 6.27 | 44986.89 | WRKYGQK | C2H2 | 2 | I | 420 |
| *MsWRKY05* | Misin01G080100.1 | Chr01 | 13181193 | 13184725 | 10.03 | 43273.5 | WRKYGQK | C2H2 | 1 | IId | 402 |
| *MsWRKY06* | Misin01G144700.1 | Chr01 | 27617080 | 27617952 | 9.51 | 11896.32 | WRKYGQK | C2H2 | 1 | IId | 108 |
| *MsWRKY07* | Misin01G144800.1 | Chr01 | 27658926 | 27661691 | 8.79 | 14941.48 | WKKYGQK | C2H2 | 1 | IId | 138 |
| *MsWRKY08* | Misin01G263700.1 | Chr01 | 84350262 | 84352266 | 6.35 | 47584.95 | WRKYGQK | C2H2 | 1 | IIe | 441 |
| *MsWRKY09* | Misin01G315300.1 | Chr01 | 105230385 | 105233381 | 6.67 | 29695.24 | WRKYGQK | C2H2 | 2 | I | 270 |
| *MsWRKY10* | Misin01G341600.1 | Chr01 | 112898915 | 112899930 | 10.23 | 18701.01 | WRKYGQK | C2 | 1 | IId | 179 |
| *MsWRKY11* | Misin01G362700.1 | Chr01 | 118028855 | 118030800 | 5.97 | 33081.46 | WRKYGQK | C2HC | 1 | III | 319 |
| *MsWRKY12* | Misin01G370500.1 | Chr01 | 119779719 | 119781349 | 8.71 | 23777.31 | WRKYGEK | C2HC | 1 | III | 219 |
| *MsWRKY13* | Misin02G032200.1 | Chr02 | 4903340 | 4905683 | 9.42 | 46713.58 | WRKYGQK | C2H2 | 1 | IId | 439 |
| *MsWRKY14* | Misin02G061600.1 | Chr02 | 10345970 | 10348174 | 6.33 | 45153.2 | WRKYGQK | C2H2 | 2 | I | 419 |
| *MsWRKY15* | Misin02G062400.1 | Chr02 | 10531789 | 10536585 | 8.28 | 36115.94 | WRKYGQK | C2H2 | 1 | IIc | 342 |
| *MsWRKY16* | Misin02G074100.1 | Chr02 | 12502555 | 12505918 | 10.03 | 43237.35 | WRKYGQK | C2H2 | 1 | IId | 403 |
| *MsWRKY17* | Misin02G115600.1 | Chr02 | 21231516 | 21233686 | 8.81 | 15261.74 | WKKYGQK | C2H2 | 1 | IId | 142 |
| *MsWRKY18* | Misin02G115700.1 | Chr02 | 21277852 | 21278769 | 9.3 | 17306.11 | WRKYGQK | C2H2 | 1 | IId | 161 |
| *MsWRKY19* | Misin02G129800.1 | Chr02 | 24888414 | 24891276 | 5.13 | 29550.9 | WRKYGQK | C2HC | 1 | III | 275 |
| *MsWRKY20* | Misin02G139500.1 | Chr02 | 27977090 | 27980493 | 9.78 | 37940.88 | WRKYGQK | C2H2 | 1 | IId | 351 |
| *MsWRKY21* | Misin02G258000.1 | Chr02 | 82777287 | 82779248 | 6.21 | 51394.67 | WRKYGQK | C2H2 | 1 | IIe | 477 |
| *MsWRKY22* | Misin02G303500.1 | Chr02 | 98992567 | 99008454 | 7.04 | 51566.58 | WRKYGQK | C2H2 | 2 | I | 487 |
| *MsWRKY23* | Misin02G345000.1 | Chr02 | 109902400 | 109904305 | 5.39 | 32934.03 | WRKYGQK | C2HC | 1 | III | 316 |
| *MsWRKY24* | Misin02G360900.1 | Chr02 | 113665512 | 113666881 | 8.5 | 23918.39 | WRKYGEK | C2HC | 1 | III | 221 |
| *MsWRKY25* | Misin03G030100.1 | Chr03 | 7315277 | 7319424 | 5.91 | 40003.58 | WRKYGQK | C2H2 | 1 | IIc | 385 |
| *MsWRKY26* | Misin03G090400.1 | Chr03 | 26714328 | 26724781 | 6.53 | 65823.66 | WKIYHEK | C2H2 | 1 | III | 577 |
| *MsWRKY27* | Misin03G145800.1 | Chr03 | 58356338 | 58358329 | 5.9 | 34210.05 | WRKYGQK | C2HC | 1 | III | 323 |
| *MsWRKY28* | Misin03G145900.1 | Chr03 | 58463020 | 58464851 | 5.15 | 32889.18 | WRKYGQK | C2HC | 1 | III | 308 |
| *MsWRKY29* | Misin03G167600.1 | Chr03 | 67670898 | 67672930 | 7.5 | 34390.44 | WRKYGQK | C2H2 | 1 | IIa | 322 |
| *MsWRKY30* | Misin03G167700.1 | Chr03 | 67713031 | 67714003 | 5.67 | 27360.52 | WRKYGQK | C2H2 | 1 | IIa | 255 |
| *MsWRKY31* | Misin03G309200.1 | Chr03 | 99305845 | 99312404 | 6.22 | 65765.89 | WRKYGQK | C2H2 | 2 | I | 612 |
| *MsWRKY32* | Misin03G348200.1 | Chr03 | 105876921 | 105879425 | 6.52 | 34512.02 | WRKYGQK | C2HC | 1 | III | 334 |
| *MsWRKY33* | Misin04G012600.1 | Chr04 | 2811844 | 2816422 | 5.71 | 40011.36 | WRKYGQK | C2H2 | 1 | IIc | 388 |
| *MsWRKY34* | Misin04G103000.1 | Chr04 | 28871792 | 28881760 | 6.64 | 180043.9 | WEKFGEK | C2H2 | 1 | III | 1620 |
| *MsWRKY35* | Misin04G121800.1 | Chr04 | 37165681 | 37167742 | 8.1 | 32376.41 | WRKYGQK | C2HC | 1 | III | 310 |
| *MsWRKY36* | Misin04G159500.1 | Chr04 | 59513396 | 59515552 | 5.8 | 33477.21 | WRKYGQK | C2HC | 1 | III | 314 |
| *MsWRKY37* | Misin04G159600.1 | Chr04 | 59590103 | 59591840 | 6.27 | 35403.26 | WRKYGQK | C2HC | 1 | III | 333 |
| *MsWRKY38* | Misin04G159700.1 | Chr04 | 59628053 | 59630374 | 6.41 | 32262.04 | WRKYGQK | C2HC | 1 | III | 300 |
| *MsWRKY39* | Misin04G189100.1 | Chr04 | 70520338 | 70521730 | 8.61 | 24998.4 | WRKYGQK | C2H2 | 1 | IIa | 232 |
| *MsWRKY40* | Misin04G189300.1 | Chr04 | 70613566 | 70615037 | 6.45 | 29149.56 | WSKYGQK | C2H2 | 1 | IIa | 272 |
| *MsWRKY41* | Misin04G224400.1 | Chr04 | 80781032 | 80788531 | 6.07 | 62448.14 | WRKYGQK | C2H2 | 2 | I | 584 |
| *MsWRKY42* | Misin04G335900.1 | Chr04 | 103115716 | 103123318 | 6.2 | 65841.98 | WRKYGQK | C2H2 | 2 | I | 613 |
| *MsWRKY43* | Misin04G395900.1 | Chr04 | 113184557 | 113186904 | 9.49 | 28237.28 | WRKYGQK | C2HC | 1 | III | 266 |
| *MsWRKY44* | Misin05G004000.1 | Chr05 | 1062340 | 1065068 | 6.95 | 40831.11 | ------- | C2H2 | 0 | IIb | 398 |
| *MsWRKY45* | Misin05G004600.1 | Chr05 | 1127680 | 1130708 | 9.02 | 61031.03 | WRKYGQK | C2H2 | 1 | IIb | 586 |
| *MsWRKY46* | Misin05G039300.1 | Chr05 | 8714579 | 8716081 | 6.51 | 25862.72 | WRKYGKK | C2H2 | 1 | IIc | 247 |
| *MsWRKY47* | Misin05G039400.1 | Chr05 | 8724881 | 8727718 | 6.74 | 54286.43 | WRKYGQK | C2H2 | 1 | IIb | 529 |
| *MsWRKY48* | Misin05G043000.1 | Chr05 | 9436264 | 9443373 | 9.66 | 29836.26 | WRKYGQK | C2H2 | 1 | IIc | 282 |
| *MsWRKY49* | Misin05G044100.1 | Chr05 | 9661358 | 9662378 | 10.01 | 25167.29 | WRKYGQK | ---- | 1 | IIc | 243 |
| *MsWRKY50* | Misin05G133400.1 | Chr05 | 33763626 | 33768855 | 6.92 | 58669.54 | WRKYGQK | C2H2 | 1 | IIb | 565 |
| *MsWRKY51* | Misin05G179500.1 | Chr05 | 61537333 | 61538346 | 5.02 | 23472.75 | WRK---- | C2H2 | 1 | IIc | 218 |
| *MsWRKY52* | Misin05G181000.1 | Chr05 | 62453032 | 62454436 | 9.45 | 34432.88 | WRKYGQK | C2H2 | 1 | IIb | 334 |
| *MsWRKY53* | Misin05G204000.1 | Chr05 | 71047834 | 71051020 | 6.39 | 38748.61 | WRKYGQK | C2H2 | 1 | IIc | 358 |
| *MsWRKY54* | Misin05G204800.1 | Chr05 | 71261560 | 71263661 | 8.16 | 42492.78 | WRKYGQK | C2H2 | 1 | IIe | 399 |
| *MsWRKY55* | Misin05G223600.1 | Chr05 | 76270790 | 76274606 | 7.75 | 40925.8 | WRKYGQK | C2H2 | 1 | IIc | 392 |
| *MsWRKY56* | Misin05G245300.1 | Chr05 | 82376407 | 82381492 | 6.65 | 27467.87 | WRKYGQK | C2H2 | 1 | IIc | 257 |
| *MsWRKY57* | Misin05G247500.1 | Chr05 | 82585497 | 82587483 | 5.09 | 31767.99 | WRKYGQK | C2H2 | 1 | IId | 309 |
| *MsWRKY58* | Misin05G257300.1 | Chr05 | 84706704 | 84708239 | 8.87 | 23279.05 | WRKYGKK | C2H2 | 1 | IIc | 217 |
| *MsWRKY59* | Misin05G266000.1 | Chr05 | 87076200 | 87078166 | 4.84 | 33807.07 | WRKYGQK | C2H2 | 1 | IIe | 311 |
| *MsWRKY60* | Misin05G314300.1 | Chr05 | 97084382 | 97087219 | 6.07 | 58493.75 | WRKYGQK | C2H2 | 2 | I | 547 |
| *MsWRKY61* | Misin05G318500.1 | Chr05 | 97853542 | 97855451 | 6.13 | 28871.07 | WRKYGQK | C2HC | 1 | III | 273 |
| *MsWRKY62* | Misin05G318700.1 | Chr05 | 97894342 | 97897352 | 5.55 | 35408.38 | WRKYGQK | C2HC | 1 | III | 321 |
| *MsWRKY63* | Misin05G318800.1 | Chr05 | 97904621 | 97907196 | 5.88 | 39828.44 | WRKYGQK | C2HC | 1 | III | 365 |
| *MsWRKY64* | Misin05G318900.1 | Chr05 | 97921736 | 97926790 | 5.56 | 29563.32 | WRKYGQK | C2HC | 1 | III | 263 |
| *MsWRKY65* | Misin05G319000.1 | Chr05 | 97947986 | 97954617 | 5.15 | 24871.51 | ------- | C2HC | 0 | III | 222 |
| *MsWRKY66* | Misin05G341800.1 | Chr05 | 102614804 | 102620037 | 6.21 | 37871.09 | WRKYGQK | C2H2 | 1 | IId | 360 |
| *MsWRKY67* | Misin06G033600.1 | Chr06 | 8287503 | 8288890 | 6.88 | 25986.1 | WRKYGKK | C2H2 | 1 | IIc | 248 |
| *MsWRKY68* | Misin06G033700.1 | Chr06 | 8316137 | 8318460 | 6.6 | 54945.21 | WRKYGQK | C2H2 | 1 | IIb | 535 |
| *MsWRKY69* | Misin06G035300.1 | Chr06 | 8827872 | 8837074 | 9.84 | 30409.98 | WRKYGQK | C2H2 | 1 | IIc | 288 |
| *MsWRKY70* | Misin06G118100.1 | Chr06 | 32022401 | 32028104 | 6.68 | 58207.58 | WRKYGQK | C2H2 | 1 | IIb | 559 |
| *MsWRKY71* | Misin06G173600.1 | Chr06 | 64362468 | 64364136 | 5.41 | 25009.44 | WRKYGKK | C2H2 | 1 | IIc | 230 |
| *MsWRKY72* | Misin06G176500.1 | Chr06 | 65837433 | 65838824 | 9.46 | 34595.22 | WRKYGQK | C2H2 | 1 | IIb | 330 |
| *MsWRKY73* | Misin06G194000.1 | Chr06 | 71485939 | 71489452 | 6.64 | 37778.45 | WRKYGQK | C2H2 | 1 | IIe | 352 |
| *MsWRKY74* | Misin06G201700.1 | Chr06 | 74689039 | 74691538 | 6.65 | 35420.7 | WRKYGQK | C2HC | 1 | III | 341 |
| *MsWRKY75* | Misin06G223300.1 | Chr06 | 81494458 | 81497861 | 6.95 | 40981.74 | WRKYGQK | C2H2 | 1 | IIc | 394 |
| *MsWRKY76* | Misin06G257000.1 | Chr06 | 89646615 | 89650205 | 6.29 | 27463.97 | WRKYGQK | C2H2 | 1 | IIc | 256 |
| *MsWRKY77* | Misin06G263000.1 | Chr06 | 91159450 | 91161480 | 4.82 | 33231.57 | WRKYGQK | C2H2 | 1 | IIe | 308 |
| *MsWRKY78* | Misin06G304500.1 | Chr06 | 98736612 | 98740191 | 5.98 | 26953.02 | WRKYGQK | C2HC | 1 | III | 238 |
| *MsWRKY79* | Misin06G304600.1 | Chr06 | 98786343 | 98796970 | 5.61 | 29373.08 | WRKYGQK | C2HC | 1 | III | 262 |
| *MsWRKY80* | Misin06G304700.1 | Chr06 | 98801362 | 98804326 | 7.08 | 39644.48 | WRKYGQK | C2HC | 1 | III | 365 |
| *MsWRKY81* | Misin06G304800.1 | Chr06 | 98822340 | 98825116 | 6.14 | 33304.99 | WRKYGQK | C2HC | 1 | III | 308 |
| *MsWRKY82* | Misin06G304900.1 | Chr06 | 98833287 | 98834794 | 5.98 | 29436.72 | WRKYGQK | C2HC | 1 | III | 276 |
| *MsWRKY83* | Misin06G308400.1 | Chr06 | 99650347 | 99653285 | 6.65 | 56227.46 | WRKYGQK | C2H2 | 2 | I | 524 |
| *MsWRKY84* | Misin06G319700.1 | Chr06 | 101544841 | 101551366 | 7.72 | 35540.77 | WRKYGQK | C2H2 | 1 | IId | 337 |
| *MsWRKY85* | Misin07G063600.1 | Chr07 | 11867503 | 11869447 | 8.81 | 38782.96 | WRKYGQK | C2H2 | 1 | IIa | 361 |
| *MsWRKY86* | Misin07G113800.1 | Chr07 | 22968339 | 22970872 | 5.77 | 40000.76 | WRKYGQK | C2H2 | 1 | IIe | 375 |
| *MsWRKY87* | Misin07G139300.1 | Chr07 | 29364149 | 29365802 | 9.23 | 11245.87 | WRKYGQK | C2H2 | 2 | I | 98 |
| *MsWRKY88* | Misin07G156800.1 | Chr07 | 33883167 | 33890997 | 6.45 | 62499.9 | WRKYGQK | C2H2 | 2 | I | 577 |
| *MsWRKY89* | Misin07G221700.1 | Chr07 | 48363328 | 48367783 | 6.18 | 73536.66 | WRKYGQK | C2H2 | 2 | I | 680 |
| *MsWRKY90* | Misin07G320900.1 | Chr07 | 80414542 | 80415531 | 6.49 | 35113.11 | WRKYGQK | C2HC | 1 | III | 329 |
| *MsWRKY91* | Misin07G336100.1 | Chr07 | 97182907 | 97186054 | 9.53 | 32283.89 | WRKYGQK | C2H2 | 1 | IId | 304 |
| *MsWRKY92* | Misin07G427800.1 | Chr07 | 133666880 | 133670859 | 8.74 | 26212.78 | WRKYGQK | C2H2 | 1 | IIc | 237 |
| *MsWRKY93* | Misin07G453800.1 | Chr07 | 139859854 | 139863939 | 5.97 | 50932.84 | WRKYGQK | C2H2 | 1 | IIe | 487 |
| *MsWRKY94* | Misin07G454800.1 | Chr07 | 140141540 | 140145937 | 5.74 | 50614.47 | WRKYGQK | C2H2 | 1 | IIe | 487 |
| *MsWRKY95* | Misin07G514500.1 | Chr07 | 153014613 | 153017365 | 5.33 | 60875.02 | WRKYGQK | C2H2 | 1 | IIb | 575 |
| *MsWRKY96* | Misin08G064100.1 | Chr08 | 13397841 | 13399660 | 8.81 | 38153.22 | WRKYGQK | C2H2 | 1 | IIa | 357 |
| *MsWRKY97* | Misin08G113400.1 | Chr08 | 31140937 | 31143013 | 5.6 | 39155.69 | WRKYGQK | C2H2 | 1 | IIe | 368 |
| *MsWRKY98* | Misin08G130600.1 | Chr08 | 40498773 | 40500342 | 9.46 | 32546.22 | WRKYGQK | C2H2 | 1 | IId | 305 |
| *MsWRKY99* | Misin08G223600.1 | Chr08 | 74637099 | 74641364 | 8.47 | 26138.66 | WRKYGQK | C2H2 | 1 | IIc | 236 |
| *MsWRKY100* | Misin08G295700.1 | Chr08 | 89997647 | 90000423 | 5.41 | 64800.38 | WRKYGQK | C2H2 | 1 | IIb | 604 |
| *MsWRKY101* | Misin09G094400.1 | Chr09 | 31825786 | 31831560 | 6.09 | 142969.7 | WRKYGQK | C2H2 | 1 | III | 1269 |
| *MsWRKY102* | Misin09G124800.1 | Chr09 | 48969562 | 48973277 | 9.32 | 23326.47 | WRKYGQK | C2H2 | 1 | IIc | 212 |
| *MsWRKY103* | Misin09G168300.1 | Chr09 | 69478619 | 69480158 | 5.16 | 35435.27 | WRKYGEK | C2HC | 1 | III | 315 |
| *MsWRKY104* | Misin09G168400.1 | Chr09 | 69516272 | 69517802 | 5.23 | 35384.37 | CRKYGEK | C2HC | 1 | III | 315 |
| *MsWRKY105* | Misin10G075100.1 | Chr10 | 22664223 | 22667110 | 7.58 | 68606.04 | WRKYGR- | C2HC | 1 | III | 602 |
| *MsWRKY106* | Misin10G075200.1 | Chr10 | 22667111 | 22669574 | 11.79 | 21954.27 | ------- | C2 | 0 | III | 197 |
| *MsWRKY107* | Misin10G090500.1 | Chr10 | 28806465 | 28809996 | 9.46 | 23366.54 | WRKYGQK | C2H2 | 1 | IIc | 211 |
| *MsWRKY108* | Misin10G147700.1 | Chr10 | 57752929 | 57755316 | 9.09 | 45494.27 | WNKYSQK | C2HC | 1 | III | 402 |
| *MsWRKY109* | Misin10G168900.1 | Chr10 | 64290186 | 64291933 | 8.31 | 28207.55 | WRKYGEK | C2HC | 1 | III | 251 |
| *MsWRKY110* | Misin11G033500.1 | Chr11 | 21369493 | 21371062 | 9.69 | 31121.11 | WRKYGQK | C2H2 | 1 | IId | 290 |
| *MsWRKY111* | Misin11G109800.1 | Chr11 | 50960040 | 50966140 | 6.99 | 76641.46 | WRKYGQK | C2H2 | 2 | I | 722 |
| *MsWRKY112* | Misin11G161900.1 | Chr11 | 61907380 | 61911291 | 8.87 | 26425.98 | WRKYGQK | C2H2 | 1 | IIc | 241 |
| *MsWRKY113* | Misin11G172000.1 | Chr11 | 63970651 | 63972274 | 10.11 | 32836.5 | WRKYGQK | C2H2 | 1 | IId | 311 |
| *MsWRKY114* | Misin11G177600.1 | Chr11 | 64766776 | 64770389 | 5.42 | 51673.75 | WRKYGQK | C2H2 | 1 | IIe | 490 |
| *MsWRKY115* | Misin12G114700.1 | Chr12 | 53371560 | 53377084 | 6.71 | 76479.3 | WRKYGQK | C2H2 | 2 | I | 721 |
| *MsWRKY116* | Misin12G168300.1 | Chr12 | 64849948 | 64854472 | 8.58 | 26693.31 | WRKYGQK | C2H2 | 1 | IIc | 246 |
| *MsWRKY117* | Misin12G221100.1 | Chr12 | 75273661 | 75277569 | 5.4 | 51312.26 | WRKYGQK | C2H2 | 1 | IIe | 490 |
| *MsWRKY118* | Misin12G224200.1 | Chr12 | 75983746 | 75985597 | 10.12 | 33577.23 | WRKYGQK | C2H2 | 1 | IId | 320 |
| *MsWRKY119* | Misin13G053300.1 | Chr13 | 15284932 | 15287835 | 9.19 | 23715.16 | WRKYGQK | C2H2 | 2 | I | 214 |
| *MsWRKY120* | Misin13G053800.1 | Chr13 | 15379156 | 15382102 | 8.1 | 24999.43 | WRKYGQK | C2H2 | 2 | I | 225 |
| *MsWRKY121* | Misin13G065100.1 | Chr13 | 19010992 | 19011900 | 9.97 | 31219.41 | WRKYGQK | C2H2 | 1 | IId | 302 |
| *MsWRKY122* | Misin13G077200.1 | Chr13 | 26514539 | 26521340 | 6.14 | 61765.12 | WRKYGQK | C2H2 | 2 | I | 568 |
| *MsWRKY123* | Misin14G000600.1 | Chr14 | 88281 | 90014 | 8.39 | 20741.62 | WRKYGQK | C2H2 | 2 | I | 187 |
| *MsWRKY124* | Misin14G044100.1 | Chr14 | 10368810 | 10372904 | 9.05 | 25295.99 | WRKYGEK | C2HC | 1 | III | 225 |
| *MsWRKY125* | Misin14G044300.1 | Chr14 | 10410602 | 10412653 | 6.45 | 30496.32 | WRKYGQK | C2HC | 1 | III | 269 |
| *MsWRKY126* | Misin14G044400.1 | Chr14 | 10415330 | 10420545 | 9.23 | 22949.89 | WRKYGQK | C2 | 1 | III | 197 |
| *MsWRKY127* | Misin14G044700.1 | Chr14 | 10486916 | 10488031 | 5.53 | 30704.25 | WRKYGQK | C2HC | 1 | III | 271 |
| *MsWRKY128* | Misin14G044900.1 | Chr14 | 10550794 | 10554423 | 5.75 | 32763.68 | WRKYGQK | C2HC | 1 | III | 316 |
| *MsWRKY129* | Misin14G045000.1 | Chr14 | 10578622 | 10579241 | 8.84 | 15262.13 | WRKYGQK | ---- | 1 | III | 146 |
| *MsWRKY130* | Misin14G045100.1 | Chr14 | 10615079 | 10617387 | 5.35 | 39100.04 | WRKYGQK | C2HC | 1 | III | 369 |
| *MsWRKY131* | Misin14G055800.1 | Chr14 | 13292467 | 13294425 | 5.81 | 33108.79 | WRKYGQK | C2HC | 1 | III | 311 |
| *MsWRKY132* | Misin14G094800.1 | Chr14 | 36988970 | 36997185 | 7.26 | 51967.19 | WRKYGQK | C2H2 | 2 | I | 489 |
| *MsWRKY133* | Misin14G145800.1 | Chr14 | 53975422 | 53978412 | 10.11 | 39116.59 | WRKYGQK | C2H2 | 1 | IId | 367 |
| *MsWRKY134* | Misin15G021700.1 | Chr15 | 5345780 | 5348381 | 6.01 | 37525.59 | WRKYGQK | C2HC | 1 | III | 354 |
| *MsWRKY135* | Misin15G022300.1 | Chr15 | 5508198 | 5510603 | 5.95 | 34408.46 | WRKYGQK | C2HC | 1 | III | 301 |
| *MsWRKY136* | Misin15G022400.1 | Chr15 | 5519610 | 5521171 | 6.41 | 30314.18 | WRKYGQK | C2HC | 1 | III | 269 |
| *MsWRKY137* | Misin15G022600.1 | Chr15 | 5554241 | 5557091 | 9.24 | 25266.01 | RRKYGEK | C2HC | 1 | III | 224 |
| *MsWRKY138* | Misin15G022700.1 | Chr15 | 5568564 | 5569944 | 6.24 | 36949.21 | WRKYGEK | C2HC | 1 | III | 349 |
| *MsWRKY139* | Misin15G059000.1 | Chr15 | 14509029 | 14511457 | 5.86 | 34045.56 | WRKYGQK | C2HC | 1 | III | 321 |
| *MsWRKY140* | Misin15G117000.1 | Chr15 | 44938827 | 44944840 | 7.26 | 52373.63 | WRKYGQK | C2H2 | 2 | I | 494 |
| *MsWRKY141* | Misin15G165100.1 | Chr15 | 61759600 | 61762535 | 10.06 | 39406.88 | WRKYGQK | C2H2 | 1 | IId | 369 |
| *MsWRKY142* | Misin15G207300.1 | Chr15 | 72991988 | 72993980 | 5.68 | 33773.54 | WRKYGQK | C2HC | 1 | III | 318 |
| *MsWRKY143* | Misin16G028000.1 | Chr16 | 6831166 | 6841544 | 6.1 | 42064.15 | WRKYGQK | C2H2 | 1 | IIe | 390 |
| *MsWRKY144* | Misin16G048900.1 | Chr16 | 10992577 | 10999877 | 6.07 | 55955.5 | WRKYGQK | C2H2 | 1 | IIb | 539 |
| *MsWRKY145* | Misin16G081700.1 | Chr16 | 22116722 | 22118179 | 8.38 | 19270.16 | WRKYGKK | C2H2 | 1 | IIc | 178 |
| *MsWRKY146* | Misin16G101800.1 | Chr16 | 37889291 | 37892295 | 6.76 | 44475.77 | WRKYGQK | C2HC | 1 | III | 415 |
| *MsWRKY147* | Misin16G105500.1 | Chr16 | 40038943 | 40041203 | 8.11 | 52702.08 | WRKYGQK | C2H2 | 2 | I | 498 |
| *MsWRKY148* | Misin16G172200.1 | Chr16 | 63685681 | 63688656 | 6.24 | 59899.74 | WRKYGQK | C2H2 | 2 | I | 567 |
| *MsWRKY149* | Misin16G174600.1 | Chr16 | 64449520 | 64453297 | 9.67 | 16747.88 | WRKYGEK | ---- | 1 | III | 149 |
| *MsWRKY150* | Misin16G174700.1 | Chr16 | 64489809 | 64491209 | 4.8 | 33581.52 | WRKYGQK | C2HC | 1 | III | 305 |
| *MsWRKY151* | Misin16G203800.1 | Chr16 | 69942927 | 69944332 | 9.25 | 25824.25 | WRKYGQK | C2H2 | 1 | IIc | 245 |
| *MsWRKY152* | Misin16G214500.1 | Chr16 | 71981919 | 71983740 | 6.51 | 22679.88 | WRKYGKK | C2H2 | 1 | IIc | 220 |
| *MsWRKY153* | Misin16G230900.1 | Chr16 | 74460403 | 74463496 | 6.14 | 45216.03 | WRKYGQK | C2H2 | 1 | IIc | 427 |
| *MsWRKY154* | Misin16G238000.1 | Chr16 | 75949728 | 75952344 | 6.47 | 55813.4 | WRKYGQK | C2H2 | 1 | IIb | 533 |
| *MsWRKY155* | Misin16G240400.1 | Chr16 | 76369804 | 76371283 | 5.64 | 31057.52 | WRKYGQK | C2HC | 1 | III | 292 |
| *MsWRKY156* | Misin16G248100.1 | Chr16 | 78361013 | 78362627 | 7.59 | 40882.18 | WRKYGQK | C2H2 | 1 | IIe | 383 |
| *MsWRKY157* | Misin17G038700.1 | Chr17 | 10631690 | 10636844 | 6.08 | 55291.53 | WRKYGQK | C2H2 | 1 | IIb | 532 |
| *MsWRKY158* | Misin17G083500.1 | Chr17 | 25376889 | 25385898 | 6.96 | 21980.17 | WRKYGKK | C2H2 | 1 | IIc | 204 |
| *MsWRKY159* | Misin17G109300.1 | Chr17 | 38677661 | 38679975 | 6.87 | 53141.5 | WRKYGQK | C2H2 | 2 | I | 507 |
| *MsWRKY160* | Misin17G113800.1 | Chr17 | 41268683 | 41271638 | 6.99 | 44427.78 | WRKYGQK | C2HC | 1 | III | 414 |
| *MsWRKY161* | Misin17G171600.1 | Chr17 | 64424660 | 64428034 | 6.08 | 21848.34 | WTKYGEK | C2HC | 1 | III | 191 |
| *MsWRKY162* | Misin17G174100.1 | Chr17 | 65394933 | 65397741 | 6.65 | 60054.88 | WRKYGQK | C2H2 | 2 | I | 563 |
| *MsWRKY163* | Misin17G209900.1 | Chr17 | 73211242 | 73212749 | 9.15 | 24778.25 | WRKYGQK | C2H2 | 1 | IIc | 234 |
| *MsWRKY164* | Misin17G216300.1 | Chr17 | 74457742 | 74459617 | 6.2 | 22606.83 | WRKYGKK | C2H2 | 1 | IIc | 218 |
| *MsWRKY165* | Misin17G243200.1 | Chr17 | 79583903 | 79586980 | 6.37 | 43647.54 | WRKYGQK | C2H2 | 1 | IIc | 414 |
| *MsWRKY166* | Misin17G243900.1 | Chr17 | 79818715 | 79821649 | 6.82 | 61955.37 | WRKYGQK | C2H2 | 1 | IIb | 589 |
| *MsWRKY167* | Misin17G248100.1 | Chr17 | 80589893 | 80591407 | 5.8 | 31233.78 | WRKYGQK | C2HC | 1 | III | 294 |
| *MsWRKY168* | Misin17G257600.1 | Chr17 | 82409895 | 82413771 | 5.06 | 35515.91 | WRKYGQK | C2H2 | 1 | IIc | 336 |
| *MsWRKY169* | Misin17G258100.1 | Chr17 | 82719255 | 82721075 | 7.6 | 32985.94 | WRKYGQK | C2H2 | 1 | IIe | 314 |
| *MsWRKY170* | Misin18G036700.1 | Chr18 | 8281538 | 8287821 | 8.86 | 67058.03 | WRKYGQK | C2H2 | 1 | IIb | 638 |
| *MsWRKY171* | Misin18G048700.1 | Chr18 | 10206417 | 10210611 | 5.82 | 41183.59 | WRKYGQK | C2HC | 1 | III | 385 |
| *MsWRKY172* | Misin18G150100.1 | Chr18 | 48766642 | 48768157 | 8.32 | 35201.51 | WRKYGQK | C2H2 | 1 | IIe | 321 |
| *MsWRKY173* | Misin18G152000.1 | Chr18 | 50380536 | 50382557 | 5.18 | 60818.16 | WRKYGEK | C2H2 | 1 | IIe | 569 |
| *MsWRKY174* | Misin18G207000.1 | Chr18 | 69577536 | 69579342 | 9.29 | 44127.96 | WRKYGQK | C2H2 | 1 | IIa | 411 |
| *MsWRKY175* | Misin19G033600.1 | Chr19 | 7916396 | 7933365 | 8.04 | 65369.63 | WRKYGQK | C2H2 | 1 | IIb | 624 |
| *MsWRKY176* | Misin19G046600.1 | Chr19 | 10576130 | 10580248 | 6.01 | 39605.9 | WRKYGQK | C2HC | 1 | III | 371 |
| *MsWRKY177* | Misin19G148800.1 | Chr19 | 49190343 | 49191582 | 7.58 | 38894.29 | WRKYGQK | C2H2 | 1 | IIe | 359 |
| *MsWRKY178* | Misin19G148900.1 | Chr19 | 49254920 | 49256188 | 7.99 | 40336.02 | WRKYGQK | C2H2 | 1 | IIe | 377 |
| *MsWRKY179* | Misin19G200400.1 | Chr19 | 69378305 | 69380812 | 9.55 | 36897.55 | WRKYGQK | C2H2 | 1 | IIa | 343 |

**Chromosome mapping and classification and phylogenetic analysis of *MsWRKY* genes**

The locations of the 179 *MsWRKY* genes were determined by MG2C v2.1 (Fig. 1). *MsWRKY* genes are distributed on all 19 Miscanthus chromosomes (Chr). Chr01-Chr19 is the chromosome (Chr) number, which shows the names and positions of the *MsWRKY*. Chr 5 had the highest number of *MsWRKY*s, with 23, representing 12.9% of the entire gene family. And the following are 18 genes on Chr 6, 14 on Chr 16, and 13 on Chr 17. Chromosomes 9, 12, and 13 had only four *MsWRKY*s each, the fewest.


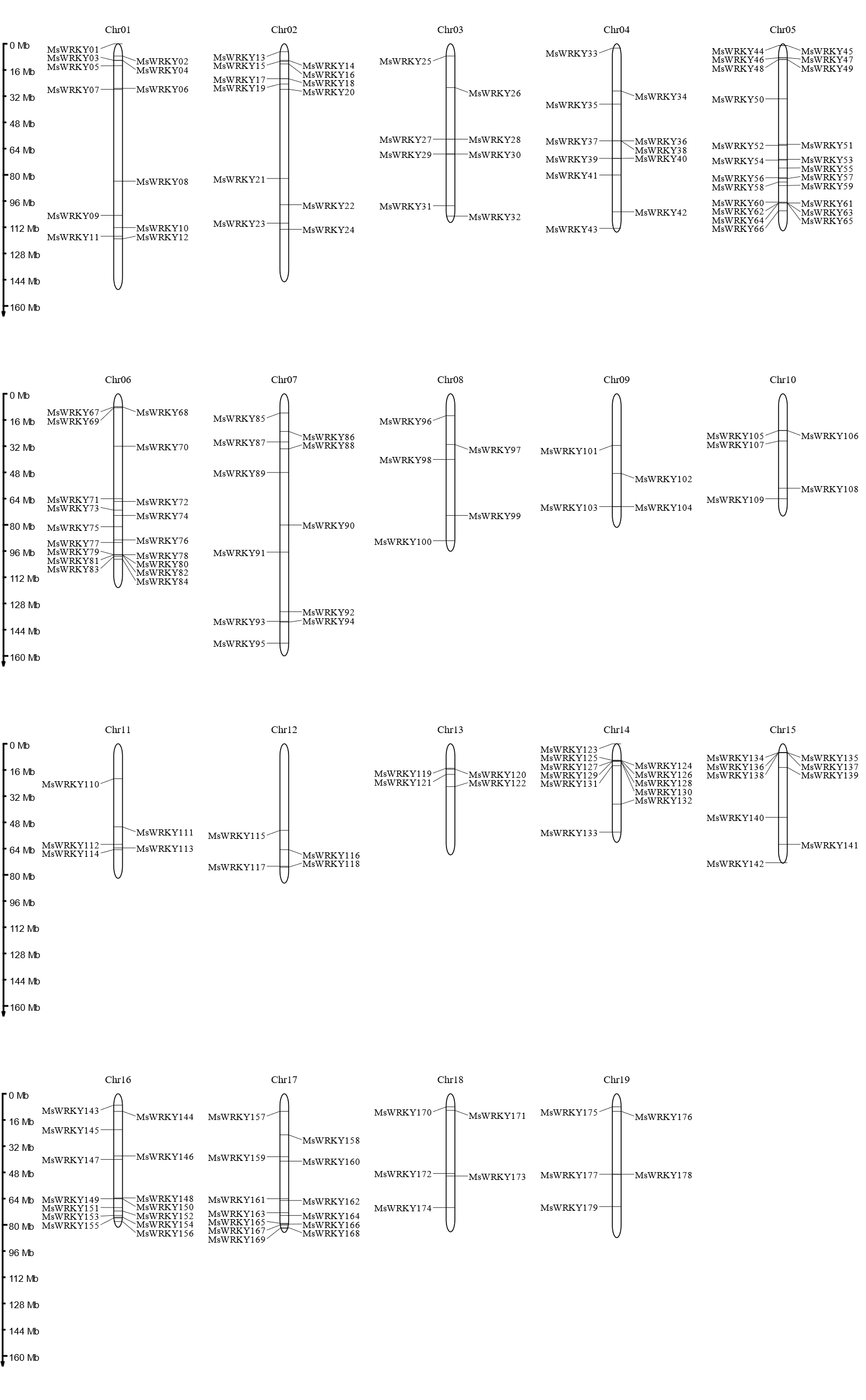


Fig 1. Distribution of 179 *Ms*WRKY genes on Miscanthus chromosomes

An unrooted phylogenetic tree to study the evolution of *MsWRKY* family members was built by the multiple sequence alignment and neighbor-joining method in MEGA7.0. The data is full-length protein sequences of 40 *SbWRKY*s and 179 *MsWRKY*s. *SbWRKY*s are used as a basis for grouping. They come from *Sorghum bicolor (L.) moench*, a Poaceae plant same as *Miscanthus sinensis*.

179 *MsWRKY*s can be divided into three major groups (I, II, and III) according to the constructed phylogenetic tree (Fig. 2). Of the 24 *MsWRKY*s in group I, all of them have two WRKYGQK motifs and 23 of them have two C2H2-type zinc finger motifs (C-X3-4-C-X22-23-H-X1-H), corresponding to two full WRKY domains. Although the protein encoded by *MsWRKY09* had only one zinc finger motif, it belonged to group I on the phylogenetic tree.

Group II has 97 protein sequences at all and was the largest group accounted for 54.2% of all putative *MsWRKY*s. Similar reports can also be found in sorghum, cucumbers, and chickpeas. Most of these proteins have one WRKY domain and the C2H2-type zinc finger motif (C-X4-5-C-X23-H-X1-H). This group was further divided into five subgroups, IIa, IIb, IIc, IId, and IIe, with 8, 16, 32, 21, and 20 members, respectively. Fifty-eight proteins belong to Group III. The proteins in this group have one WRKY domain and the C2HC-type zinc finger motif (C-X7-C-X23-H-X1-C) [43]. In summary, the classification of *MsWRKY*s indicates the diversity of these proteins. An extremely wide range of functions can be performed by these proteins.


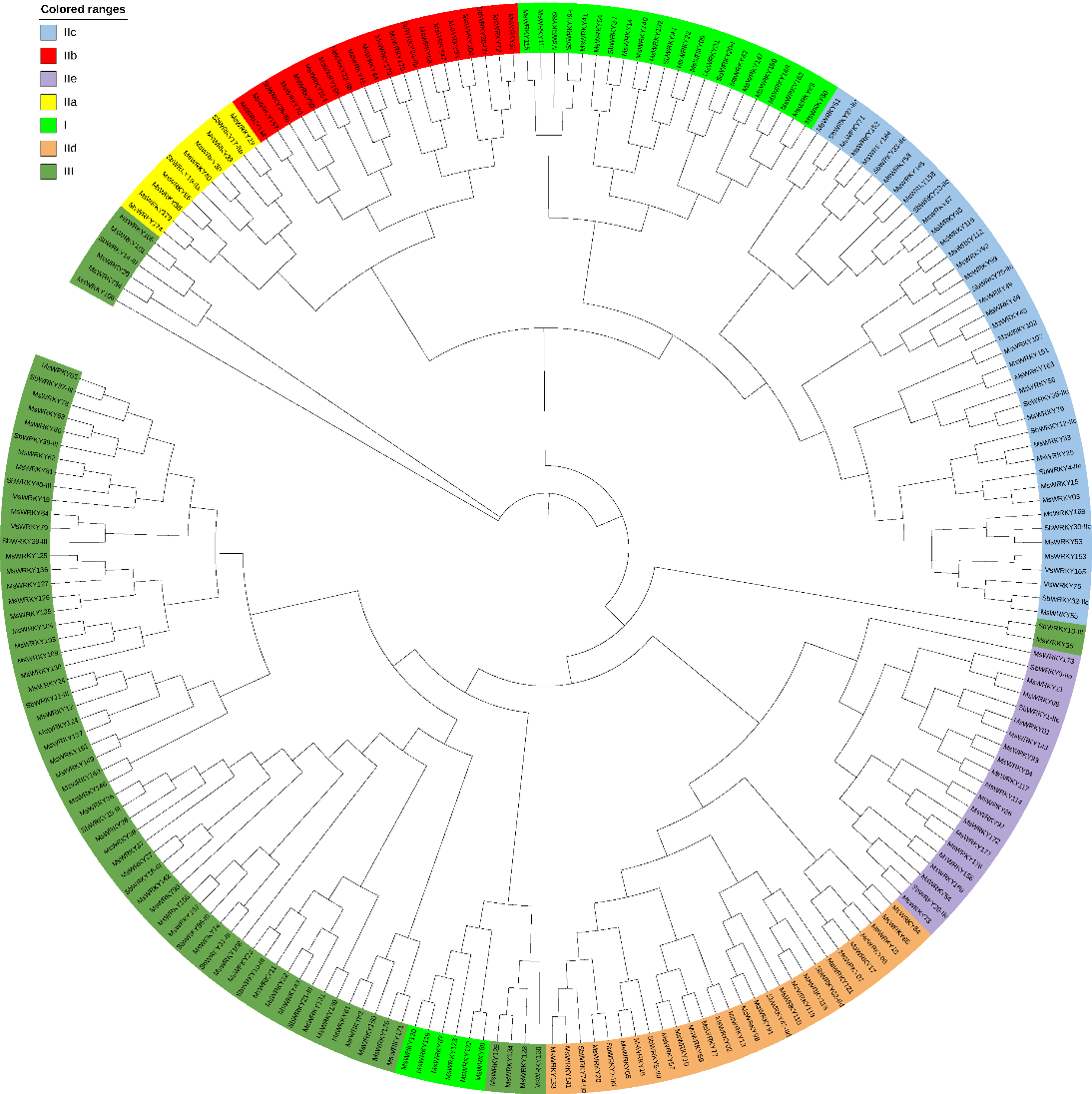


Fig 2. Phylogenetic tree of WRKY members in Miscanthus and Sorghum. All *MsWRKY*s genes were further divided into subgroups I, II, and III, and group II was further divided into subgroups IIa, IIb, IIc, IId, and IIe

**Gene structure analysis and conserved motif distribution analysis of *MsWRKY* genes**

The exon-intron structures of *MsWRKY* family members can illustrate the evolution of *MsWRKY* family members. The intron’s number of *MsWRKY* genes ranged from zero to five, whereas their size varied. The data showed that genes within the same group had certain similarities in the exon-intron distribution patterns. The *MsWRKY* genes can be indicated an important structural diversity through these results. This may represent the functional diversity between closely related members of *MsWRKY*s (Fig 3).


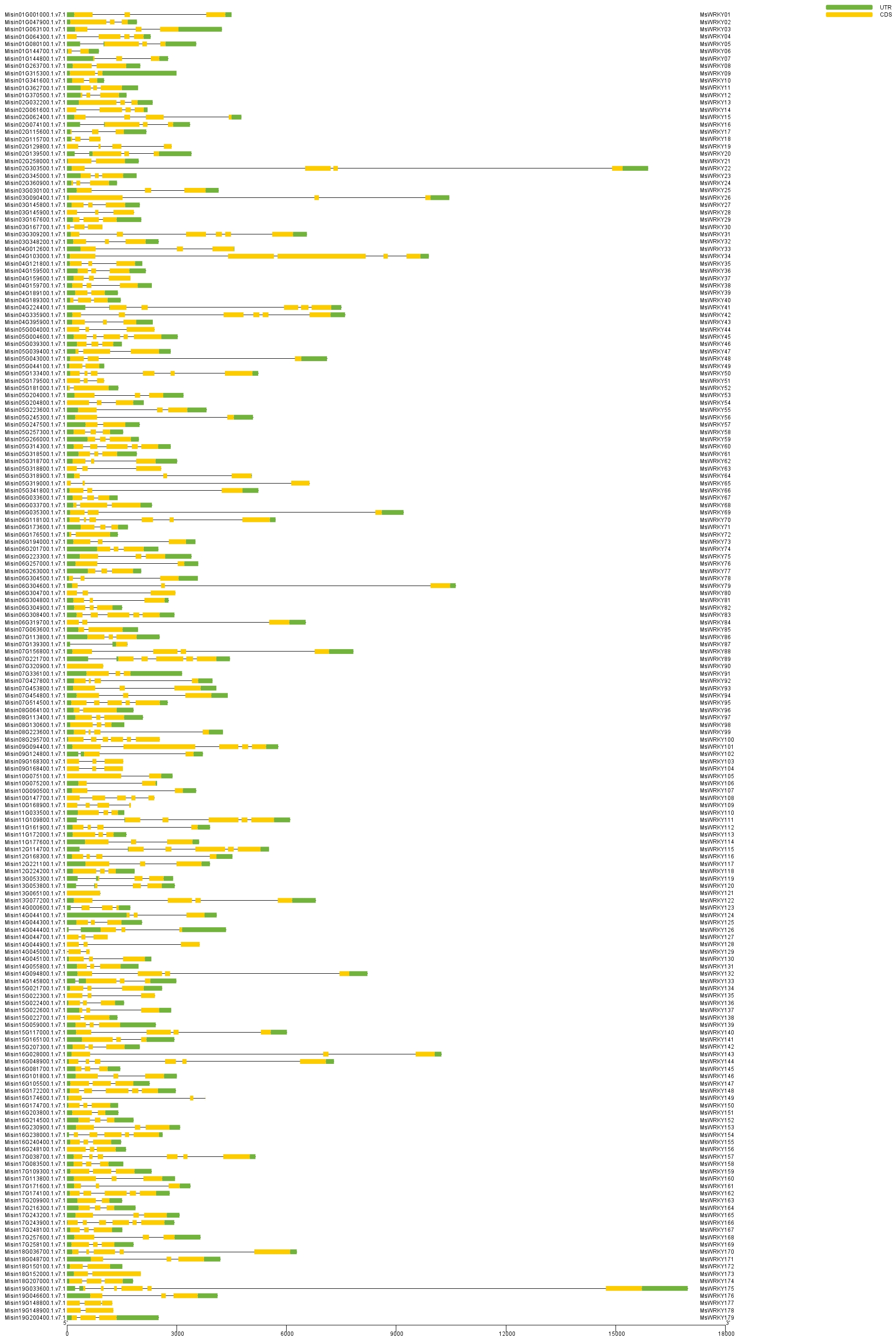


Fig 3. Exon–intron structures of *MsWRKY* genes. Exon–intron structures of *MsWRKY* genes were obtained after analysed with TBtools for gene structure. Green bars indicate upstream and downstream UTRs, yellow bars indicate coding sequences (CDS), and black lines indicate introns in the gene diagrams.

MEME (version 5.5.3), used to analyze the conserved motifs of all *MsWRKY* protein sequences, identified 20 distinct conserved motifs. The distribution of 20 conserved motifs identified by MEME in the different groups of *MsWRKY*s is shown in Fig. 4. Motifs 1 is the WRKY domain. Similar Motif structures can be found between the genes in the same group or subgroup through the results. Motifs 1, 2, 3, and 4 are found in almost all genes. Motifs 15 and 19 were unique to group I. Motifs 9 and 13 were unique to group IIb. Motifs 12 were unique to group IIe. Motifs 10, 16, and 18 were unique to group III. Some of the motifs shared by different groups. Motif 5 was shared by groups I and IIc, and motif 6 and 7 were shared by groups IIa and IIb.


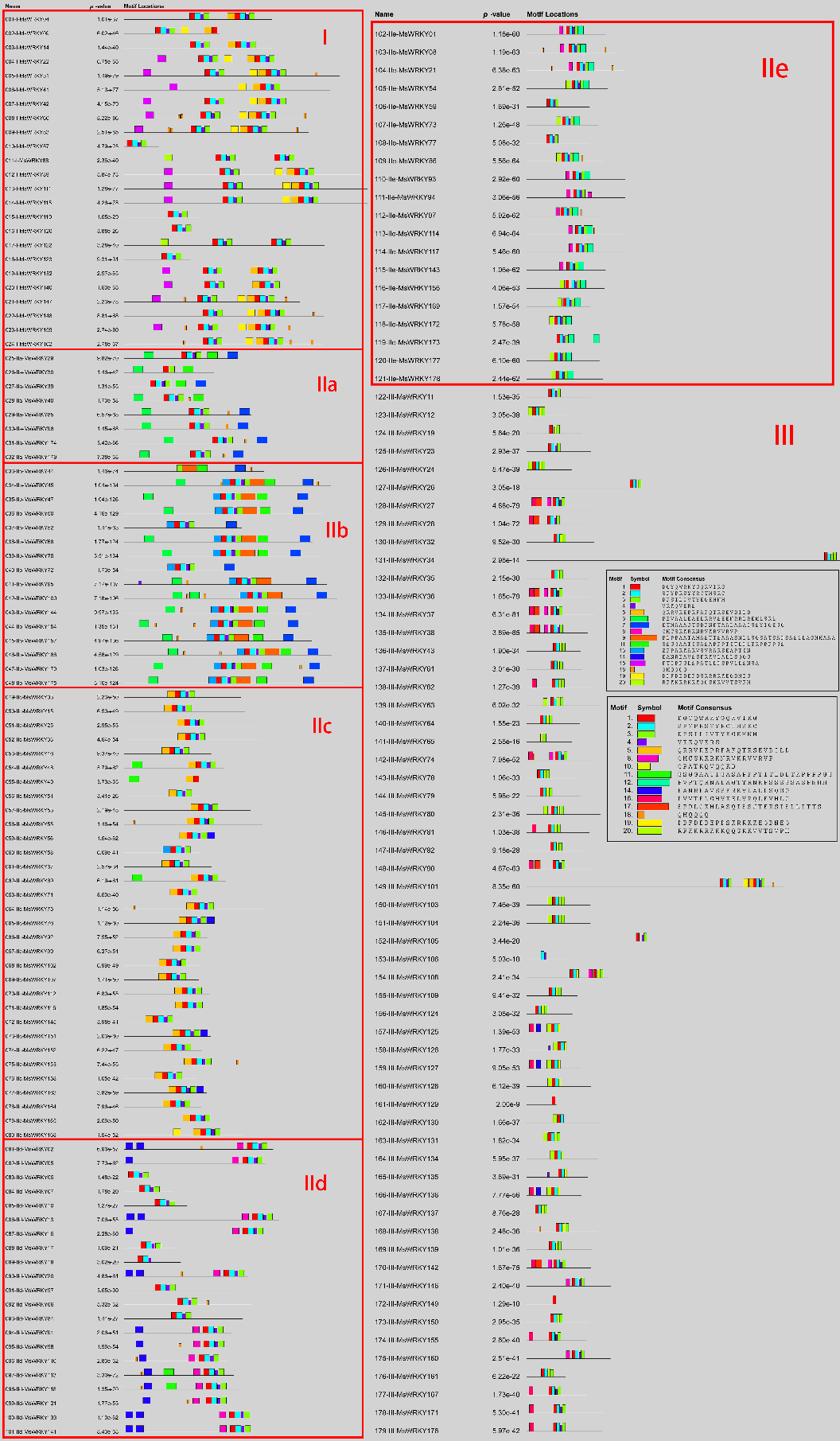


Fig 4. Motif analysis of *MsWRKY*s

**Gene ontology annotation and analysis of cis-acting elements of *MsWRKY* genes**

The Blast2GO analyzed the Gene ontology (GO) annotations of 179 *MsWRKY* proteins. The *MsWRKY* target genes can be categorized into three main categories according to different functional groups. The biological processes, molecular functions, and cellular components together make up the Gene ontology (GO) annotations. Through the enrichment analysis, the involvement of *MsWRKY* in biological processes, molecular functions, and cellular components is in Fig 5. Most *MsWRKY*s are involved in regulating cellular processes, biosynthetic processes, and different metabolic processes. Further analysis showed that most *MsWRKY*s were involved in the plant's stress to external adversity. Many *MsWRKY*s have been linked to bacterial infections and environmental stress [41]. The molecular functions of *MsWRKY*s are mainly a variety of DNA-binding and gene expression regulation. The cellular component of this protein family is mainly organelle and intracellular organelle. Most of the *MsWRKY* proteins are located in the cell nucleus.


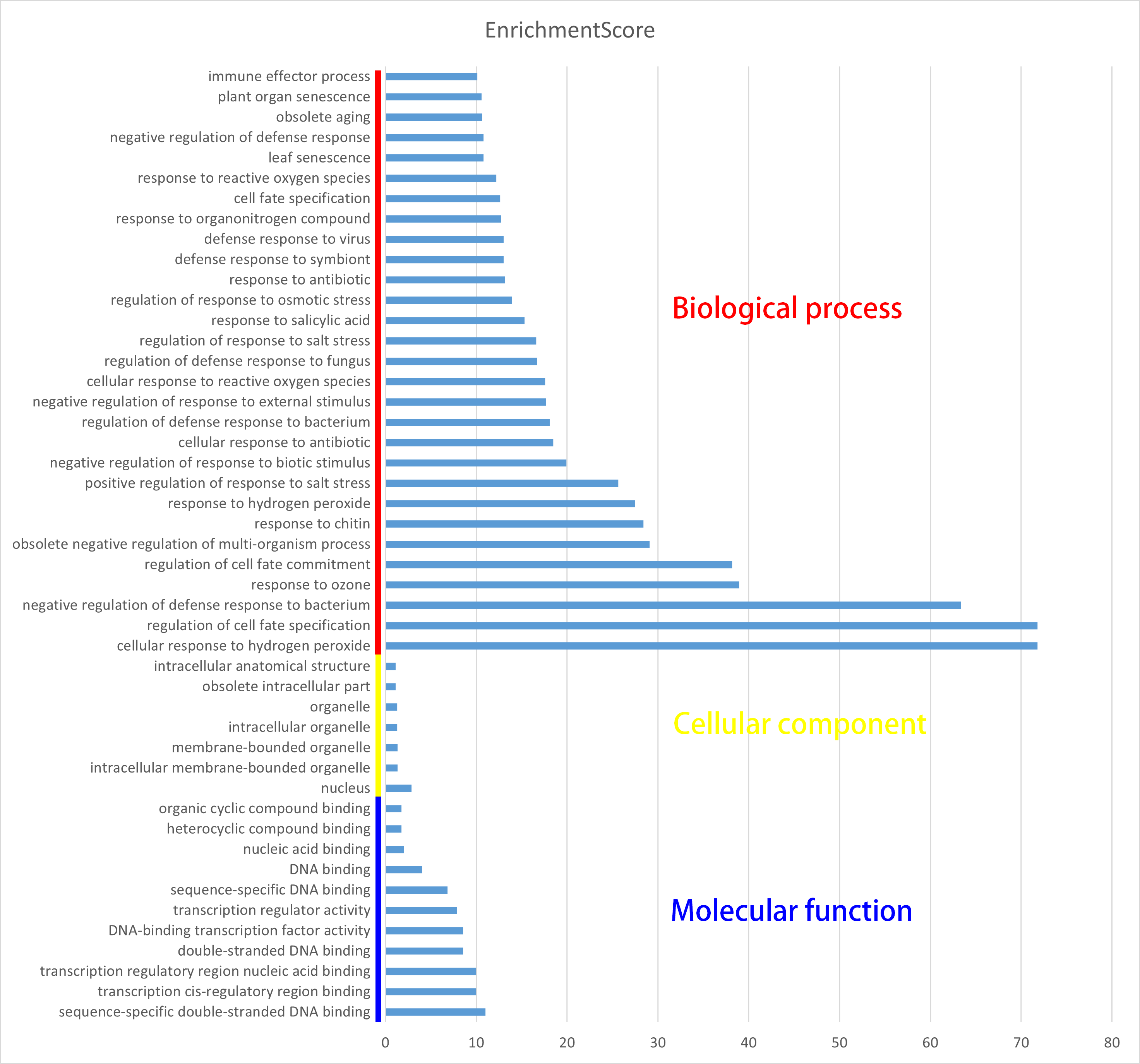


Fig 5. Gene ontology analysis of identified *MsWRKY*s

Cis-acting elements are essential sequences in regulating gene expression by transcription factors. An online tool PlantCARE was used to analyze all *MsWRKY* cis-acting elements and extracted 2000bp promoter regions upstream of all *MsWRKY* genes. The result shows that every *MsWRKY* have many cis-acting elements.

Firstly, many transcription-related cis-acting elements can be found including TATA-box, CAAT-box, A-box, HD-Zip, and W-box. The stress-responsive elements formed an important part in the cis-acting elements. This suggests that *MsWRKY* plays an important role in plants' resistance to external stress. These cis-acting elements include MBS (Anti-drought stress), LTR (anti-low temperature stress), and WUN-motif (wound-responsive elements). In addition, many of the cis-acting elements were regulated by phytohormones. ABA regulates the ABA-responsive element (ABRE). Methyl jasmonate (MeJA) responsive element (TGACG-motif and CGTCA-motif) is regulated by jasmonate phytohormones [42]. Finally, there are auxin-responsive elements (AuxRR-core and TGA-element), salicylic acid-responsive elements (TCA-element), etc. Finally, there are many light-responsive elements and other regulatory elements. At least one stress-responsive element is on all of the *MsWRKY* genes and reflects the potential functional variation of the *MsWRKY* gene.

**Synteny analysis of *MsWRKY* genes**

The segmental duplication events occurring in the Miscanthus WRKY family were investigated by conducting a synteny analysis of the *MsWRKY* genes using BLASTP and MCScanX in TBtools. As shown in Fig. 6, 19 segmental duplication events involving 53 WRKY genes were observed. Tandem duplication events, which were defined by a chromosomal region within 200 kb containing two or more genes, were widely identified for Miscanthus WRKY genes. A very large tandem duplication event was observed in the chromosome 14. These results suggested that some *MsWRKY*s were possibly generated by segmental duplication events and that the evolution of *MsWRKY* genes may have been driven, at least in part, by segmental duplication events.


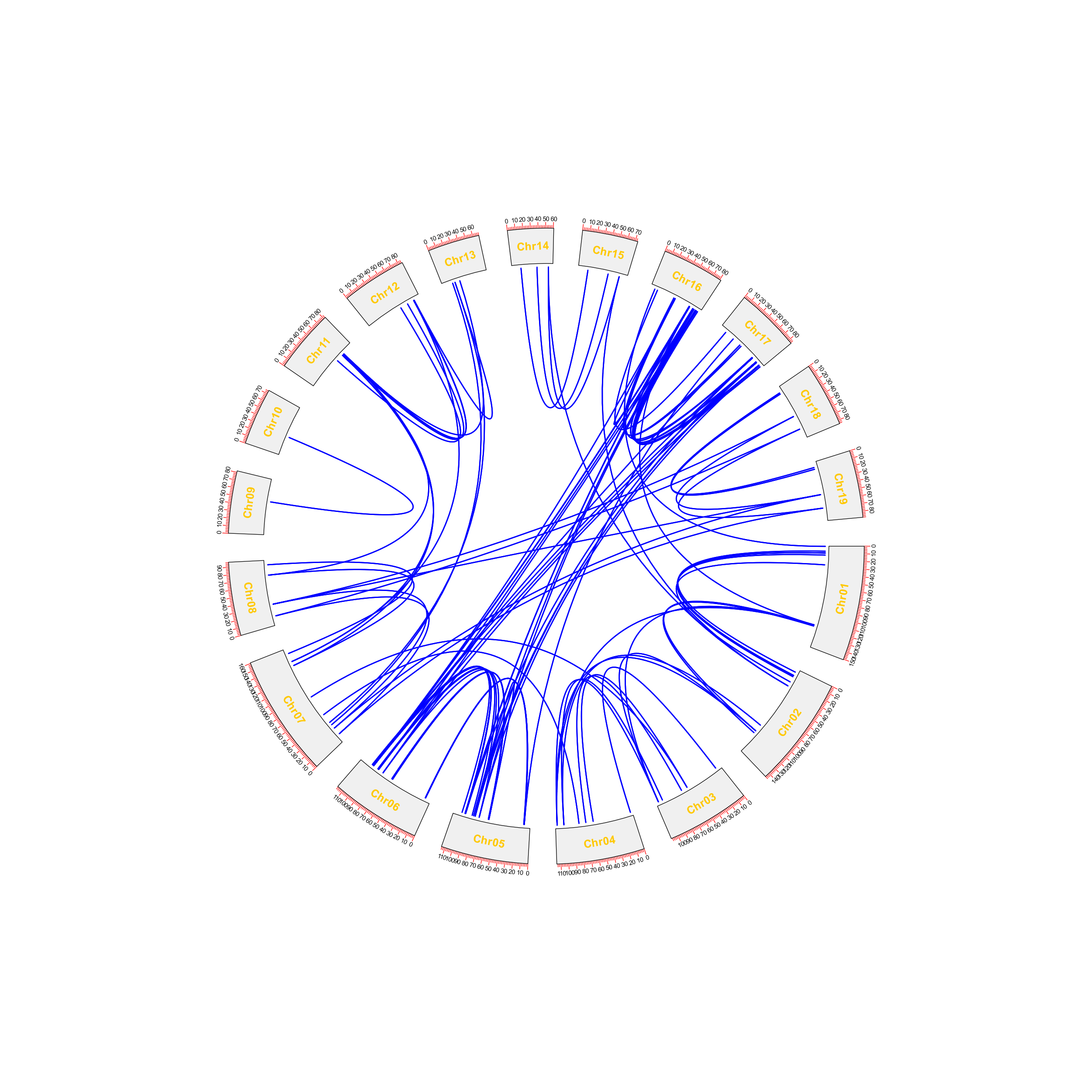


Fig 6. Schematic representations for the interchromosomal relationships of *MsWRKY*s. Blue lines show duplicated WRKY gene pairs in the Miscanthus genome

The phylogenetic mechanisms of the Miscanthus WRKY family were further explored by constructing comparative syntenic maps of cucumber associated with four representative species, including two dicots (Arabidopsis and cucumber) and two monocots (sorghum and maize) (Fig. 7). 249, 231, 28, and 27 pairs of genes showed syntenic relationships between the other four species: cucumber, Arabidopsis, sorghum and maize, respectively. A total of 249 WRKY collinear gene pairs between Miscanthus and maize were identified, followed by Miscanthus and sorghum (231), Miscanthus and cucumber (28), and Miscanthus and Arabidopsis (27). Both Miscanthus and maize belong to the Poaceae family, and more than 75.4% of the *MsWRKY* genes showed a syntenic relationship with WRKYs in maize. But some of *MsWRKY* genes were associated with more than one syntenic gene pair, indicating that WRKY genes in Poaceae family have gone through multiple rounds of duplication events. This may be the reason why monocotyledonous plants have far more WRKY genes than dicotyledonous plants. Importantly, collinear *MsWRKY09/11/60/64/83/85/112/179* genes pairs were observed between Miscanthus and all of the other four species, suggesting that these orthologous pairs may have formed before the divergence of dicotyledonous and monocotyledonous plants.


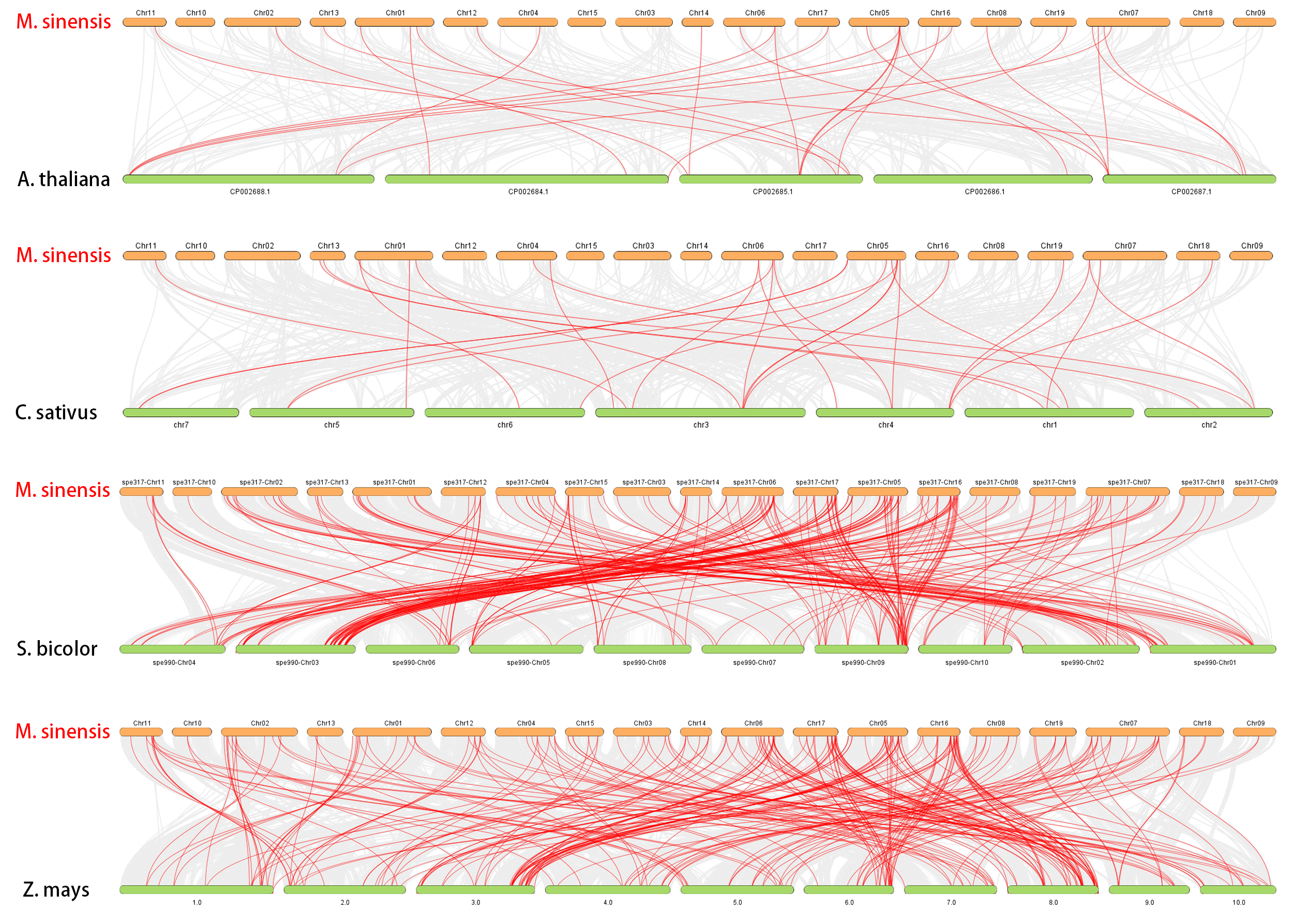


Fig 7. Synteny analysis of WRKYs between Miscanthus and other plant species. The collinear blocks are marked by gray lines, while the collinear gene pairs with WRKY genes are highlighted in the red lines. ‘*M. sinensis*’, ‘*A. thaliana*’, ‘*C. sativus*’, ‘*S. bicolor*’ and ‘*Z. mays*’ indicate *Miscanthus sinensis*, *Arabidopsis thaliana*, *Cucumis sativus*, *Sorghum bicolor*, and *Zea mays*, respectively

**Digital expression analysis of *MsWRKY* genes at different seasons and in different tissues**

The study of the temporal and spatial expression profiles of *MsWRKY* genes used the microarray data provided by the JGI database and presented the results as heatmaps by TBtools. the microarray datasets used gene expression data for *M. sinensis* and included a total of 22 samples. The samples were taken from leaf (7), rhizome (9) and stem (6). The samples were collected from plants at different times of the year and reflected the expression of the *MsWRKY* gene at different stages of plant growth. 175 of the 179 genes showed differential expression in plants. Most *MsWRKY* genes are highly expressed in rhizome. The expression patterns of *MsWRKY* in different growth and development stages were also analyzed. Firstly, the *MsWRKY* gene is first expressed in rhizome in large quantities during plant growth. Then, some *MsWRKY* genes were heavily expressed in the leaf. Eventually, some *MsWRKY* genes are over-expression in the stem when plants wither. The results showed that these genes may be involved in stress response at sensitive developmental stages to improve plant tolerance (Fig. 6).

By analyzing the gene expression heat map and cis-acting elements together, it can be found that genes activated in different periods have different characteristics. In the early stage of plant development, the expression of the *MsWRKY* gene is mostly controlled by plant hormones and light regulatory elements. In the middle stage of plant development, the *MsWRKY* gene expressed in leaves is more regulated by infection and injury. At the end of plant development, *MsWRKY* genes expressed were mostly regulated by ABA and jasmonic acid, and some were stressed by environmental conditions such as drought. This suggests that the *MsWRKY* gene plays an important role in the growth of the perennial plant *Miscanthus sinensis*.


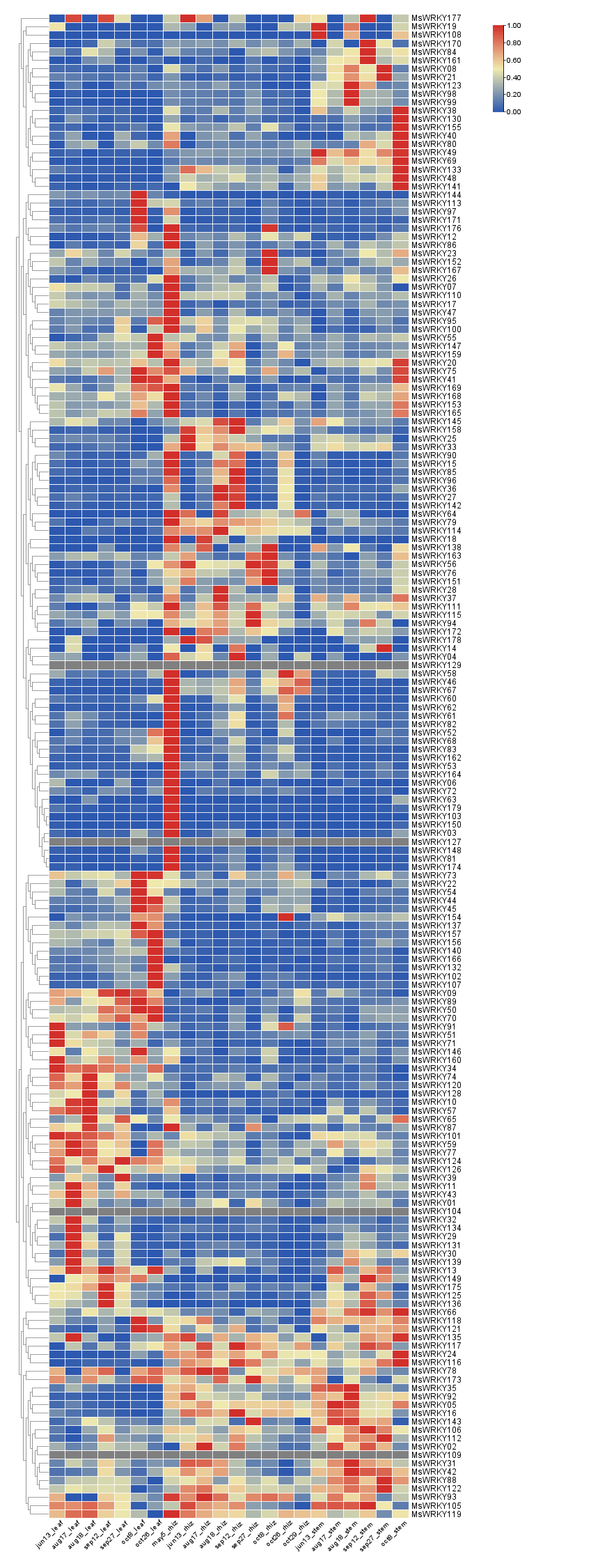


Fig 6. Heatmaps of *MsWRKY* gene expression. *MsWRKY* expression levels in different tissues and at different seasons

**Discussion**

WRKY transcription factors (TFs) are widespread in the plant kingdom and play an essential role in stress tolerance of plants. WRKY genes. This includes 66 WRKY genes in Arabidopsis, 119 WRKY genes in maize, 94 WRKY genes in sorghum, 79 WRKY genes in potatoes, 70 WRKY genes in chickpeas, and 61 WRKY genes in cucumbers. By analyzing the genomic assembly of *Miscanthus sinensis*, 179 WRKY genes were identified. As the *Miscanthus sinensis* is a paleotetraploid [27], the amount of the WRKY genes is much higher than that of normal plants but had not get much attention in previous studies. The *MsWRKY* gene is distributed on all 19 chromosomes of Miscanthus. WRKY has previously been identified as a gene that promotes flowering which has been identified as *MsWRKY92* located on chromosome 7 [28]. Conserved WRKY domains bind to the W-box motif in the promoter of WRKY target genes, which is the most important feature of the WRKY family [29, 30]. A phylogenetic analysis of all the obtained *MsWRKY* genes has been performed. By analyzing the evolutionary tree, the *MsWRKY* genes were classified. These genes were classified into groups I, II, and III according to the number of WRKY domains and the type of zinc finger motif. group II is further subdivided into five subgroups: IIa, IIb, IIc, IId, and IIe. Group I had 24 *MsWRKY* genes, group II had 97, and group III had 58. In group II, group IIc had the most *MsWRKY* genes, with 32. The proportions of these genes are similar to those found in other plants [31, 32, 33].

Most *MsWRKY* genes have a very conserved WRKYGQK motif. However, other similar sequences have been found in many genes. (*MsWRKY07 MsWRKY12 MsWRKY17 MsWRKY24 MsWRKY26 MsWRKY34 MsWRKY40 MsWRKY46 MsWRKY51 MsWRKY58 MsWRKY67 MsWRKY71 MsWRKY103 MsWRKY104 MsWRKY105 MsWRKY108 MsWRKY109 MsWRKY124 MsWRKY137 MsWRKY138 MsWRKY145 MsWRKY149 MsWRKY152 MsWRKY158 MsWRKY161 MsWRKY164 MsWRKY173*) There are even some genes that are clearly WRKY gene that are missing this sequence (*MsWRKY44 MsWRKY65 MsWRKY106*). These differences can seriously affect the ability of *MsWRKY* proteins to bind to W-box elements, which in turn affects the biological function of these proteins. Similar heptapeptide motif variations have been found in other plants, such as sorghum [4]. In soybeans, for example, two WRKY genes with WRKYGKK motif do not bind to normal W-box elements and do not work [29]. Therefore, further studies are necessary to confirm the biological function of these WRKY motif aberrant genes.

Some population-specific patterns can be found by studying the exon-intron structure of their genes. Similar *MsWRKY* genes on the evolutionary tree tend to have similar exon-intron patterns. The number of introns in the *MsWRKY* gene ranges from 0 to 5. Some *MsWRKY* genes do not contain introns, which indicates that some WRKY genes have intron loss events [40]. Intron-free genes have been found in other organisms. There are three main mechanisms by which intron-free genes are produced: reverse transcription (the integration of sequences from RNA into the genome), duplication of existing intron-free genes, and horizontal gene transfer [34]. Differences in the intron size of *MsWRKY* genes may result from gene duplication, inversion, and/or fusion events [35]. In conclusion, the diverse exon-intron structure of *MsWRKY* genes reflects the evolutionary diversity of the *MsWRKY* gene family.

Motif structural studies on the *MsWRKY* gene reflect this gene family's structural conservatism and diversity. Motif 1, 2, 3, and 4 corresponds to WRKY domains and zinc finger domains that are contained in most *MsWRKY* genes. In addition, most motifs' functions are unclear, but their distribution also has certain rules. Motifs 15 and 19 were unique to group I. Motifs 9 and 13 were unique to group IIb. Motifs 12 were unique to group IIe. Motifs 10, 16, and 18 were unique to group III. Some of the motifs shared by different groups included motif 5, shared by groups I and IIc, and motifs 6 and 7, shared by groups IIa and IIb. Motif 8 is a nuclear localization signal (NLS), mainly in groups IId, IIe, and III [39]. In conclusion, the structural differences of genes in different groups in *MsWRKY* can be clearly demonstrated in motif analysis. These motifs may reflect that these genes participate in specific biological processes and play similar biological functions.

Studying the cis-acting elements of *MsWRKY* can obtain more information about the gene expression of the *MsWRKY* gene family. Firstly, many transcription-related cis-acting elements [38] including TATA-box, CAAT-box, A-box and HD-Zip are essential for gene expression, and most are involved in constructing transcription complexes. In addition, there is also a batch of cis-acting elements regulated by phytohormones. ABA regulates the ABA-responsive element (ABRE). Methyl jasmonate (MeJA) responsive element (TGACG-motif and CGTCA-motif) is regulated by jasmonate phytohormones. In addition, there are auxin-responsive elements (AuxRR-core and TGA-element), salicylic acid-responsive elements (TCA-element), and so on. A variety of biological and abiotic stresses also regulate these genes. These cis-acting elements include MBS (Anti-drought stress), LTR (anti-low temperature stress), and WUN-motif (wound-responsive elements). At the same time, W-box was also found in the promoter region of many *MsWRKY* genes. This suggests that there is also mutual regulation between WRKY genes [36, 37]. Studies on cis-acting elements of *MsWRKY* reflect the diversity of the *MsWRKY* gene in gene expression regulation.

Comparative syntenic maps can also be used to identify potential resistance genes. The essential roles of WRKY TFs in plant growth, development, and stress tolerance are supported by WRKY gene expression data from several species. From extensive studies in the model plant Arabidopsis, many *AtWRKY*s have been functionally characterized. Therefore, identifying the closest Arabidopsis homologue(s) of individual *MsWRKY*s may provide a hint as to their potential functions. For example, *MsWRKY131* and *MsWRKY139* gene are most closely related to the Arabidopsis *AtWRKY70* and *AtWRKY54*, which have been reported to modulate osmotic stress tolerance by regulating stomatal aperture [44]. The Miscanthus genes *MsWRKY85* *MsWRKY96* and *MsWRKY179* is the putative ortholog of *AtWRKY18*, *AtWRKY40*, and *AtWRKY60*, which are involved in abscisic acid signaling and abiotic stress [45]. Additionally, they are the putative ortholog of maize *ZmWRKY40*, which confers drought resistance when expressed in transgenic Arabidopsis [46]. A putative ortholog of *MsWRKY21* is *AtWRKY57*, which can improve drought tolerance through elevated abscisic acid levels [47].

Existing transcriptome data of *Miscanthus sinensis* can be used to analyze the expression of *MsWRKY* gene in different stages of Miscanthus development and expression patterns in different tissues. Future studies on the function of the *MsWRKY* gene are needed to study the homologous genes of other model plants (Arabidopsis) and related plants (other grasses). Future research should focus on the effect of *MsWRKY* on plant growth and development and against adverse winter environments. In addition, the role of the *MsWRKY* gene in combating environmental stress in plants is also worth studying. Our research results are helpful in providing a research basis for subsequent researchers.

**Conclusion**

In this study, 179 WRKY genes were identified from *Miscanthus sinensis*. The identification, chromosome mapping, classification, phylogenetic analysis, gene structure analysis, conserved motif distribution analysis, gene ontology annotation, analysis of cis-acting elements, and digital expression pattern analysis have been performed. Through digital expression pattern analysis, the specific expression of the *MsWRKY* gene in different developmental stages and different parts of plants was found. At the same time, some *MsWRKY* genes may play an important role in plant stress resistance. In conclusion, this study is conducive to further research on the important functions of the WRKY gene in response to abiotic and biological stresses.

**Declaration of Interest Statement**

We declare that we have no financial or personal relationship with others or organizations that can inappropriately influence our work. No professional or other personal interest of any nature or kind in any product, service, or company could be construed as influencing the position presented in, or the review of, the manuscript entitled.

**Data availability**

All custom scripts used for parsing and analyzing transposable elements, gene families, and gene expression, as described in Supplementary Notes, are available at JGI and NCBI database [https://data.jgi.doe.gov/refine-download/phytozome?organism=Msinensis&expanded=497&_gl=1*13m4cmx*_ga*MTQwODM0NDIwMy4xNjk4MDQwMzEw*_ga_YBLMHYR3C2*MTY5ODA0MDMwOS4xLjEuMTY5ODA0MDgwMS4wLjAuMA..] [https://www.ncbi.nlm.nih.gov/bioproject/PRJNA575573] [https://www.ncbi.nlm.nih.gov/bioproject/PRJNA346689]

**References**

[1] Ishiguro, S., & Nakamura, K. (1994). Characterization of a cDNA encoding a novel DNA-binding protein, SPF1, that recognizes SP8 sequences in the 5′ upstream regions of genes coding for sporamin and β-amylase from sweet potato. *Molecular and General Genetics MGG, 244*, 563-571.

[2] Wang, Q., Wang, M., Zhang, X., Hao, B., Kaushik, S. K., & Pan, Y. (2011). WRKY gene family evolution in Arabidopsis thaliana. *Genetica, 139*, 973-983.

[3] Wei, K. F., Chen, J., Chen, Y. F., Wu, L. J., & Xie, D. X. (2012). Molecular phylogenetic and expression analysis of the complete WRKY transcription factor family in maize. DNA research, *19*(2), 153-164.

[4] Baillo, E. H., Hanif, M. S., Guo, Y., Zhang, Z., Xu, P., & Algam, S. A. (2020). Genome-wide Identification of WRKY transcription factor family members in sorghum (Sorghum bicolor (L.) moench). *PloS one, 15*(8), e0236651.

[5] Zhang, C., Wang, D., Yang, C., Kong, N., Shi, Z., Zhao, P., & Chen, Q. (2017). Genome-wide identification of the potato WRKY transcription factor family. *PloS one, 12*(7), e0181573.

[6] Waqas, M., Azhar, M. T., Rana, I. A., Azeem, F., Ali, M. A., Nawaz, M. A., & Atif, R. M. (2019). Genome-wide identification and expression analyses of WRKY transcription factor family members from chickpea (Cicer arietinum L.) reveal their role in abiotic stress-responses. *Genes & genomics, 41*, 467-481.

[7] Chen, C., Chen, X., Han, J., Lu, W., & Ren, Z. (2020). Genome-wide analysis of the WRKY gene family in the cucumber genome and transcriptome-wide identification of WRKY transcription factors that respond to biotic and abiotic stresses. *BMC Plant Biology, 20*, 1-19.

[8] Eulgem, T., Rushton, P. J., Robatzek, S., & Somssich, I. E. (2000). The WRKY superfamily of plant transcription factors. *Trends in plant science, 5*(5), 199-206.

[9] Rushton, P. J., Somssich, I. E., Ringler, P., & Shen, Q. J. (2010). WRKY transcription factors. *Trends in plant science, 15*(5), 247-258.

[10] Jin, W., & Wu, F. (2015). Characterization of miRNAs associated with Botrytis cinerea infection of tomato leaves. *BMC Plant Biology, 15*, 1-14.

[11] Bakshi, M., & Oelmüller, R. (2014). WRKY transcription factors: Jack of many trades in plants. *Plant signaling & behavior, 9*(2), e27700.

[12] Pandey, S. P., & Somssich, I. E. (2009). The role of WRKY transcription factors in plant immunity. *Plant physiology, 150*(4), 1648-1655.

[13] Finatto, T., Viana, V. E., Woyann, L. G., Busanello, C., Maia, L. C. D., & Oliveira, A. C. D. (2018). Can WRKY transcription factors help plants to overcome environmental challenges? *Genetics and molecular biology, 41*, 533-544.

[14] Wu, X., Shiroto, Y., Kishitani, S., Ito, Y., & Toriyama, K. (2009). Enhanced heat and drought tolerance in transgenic rice seedlings overexpressing OsWRKY11 under the control of HSP101 promoter. *Plant Cell Reports, 28*, 21-30.

[15] Fu, Q. T., & Yu, D. Q. (2010). Expression profiles of AtWRKY25, AtWRKY26 and AtWRKY33 under abiotic stresses. *Yi chuan Hereditas, 32*(8), 848-856.

[16] Jing, Z., & Liu, Z. (2018). Genome-wide identification of WRKY transcription factors in kiwifruit (Actinidia spp.) and analysis of WRKY expression in responses to biotic and abiotic stresses. *Genes & genomics, 40*, 429-446.

[17] Rushton, D. L., Tripathi, P., Rabara, R. C., Lin, J., Ringler, P., Boken, A. K., & Rushton, P. J. (2012). WRKY transcription factors: key components in abscisic acid signalling. *Plant biotechnology journal, 10*(1), 2-11.

[18] Gao, H., Wang, Y., Xu, P., & Zhang, Z. (2018). Overexpression of a WRKY transcription factor TaWRKY2 enhances drought stress tolerance in transgenic wheat. *Frontiers in plant science, 9*, 997.

[19] Zhang, L., Zhao, T., Sun, X., Wang, Y., Du, C., Zhu, Z., & Xin, H. (2019). Overexpression of VaWRKY12, a transcription factor from Vitis amurensis with increased nuclear localization under low temperature, enhances cold tolerance of plants. *Plant molecular biology, 100*, 95-110.

[20] Mitros, T., Session, A. M., James, B. T., Wu, G. A., Belaffif, M. B., Clark, L. V., & Rokhsar, D. S. (2020). Genome biology of the paleotetraploid perennial biomass crop Miscanthus. *Nature Communications, 11*(1), 5442.

[21] Letunic, I., & Bork, P. (2018). 20 years of the SMART protein domain annotation resource. *Nucleic acids research, 46*(D1), D493-D496.

[22] Kumar, S., Stecher, G., & Tamura, K. (2016). MEGA7: molecular evolutionary genetics analysis version 7.0 for bigger datasets. *Molecular biology and evolution, 33*(7), 1870-1874.

[23] Zhang, C., Wang, D., Yang, C., Kong, N., Shi, Z., Zhao, P., & Chen, Q. (2017). Genome-wide identification of the potato WRKY transcription factor family. *PloS one, 12*(7), e0181573.

[24] Chen, C., Chen, H., Zhang, Y., Thomas, H. R., Frank, M. H., He, Y., & Xia, R. (2020). TBtools: an integrative toolkit developed for interactive analyses of big biological data. *Molecular plant, 13*(8), 1194-1202.

[25] Zou, Z., Yang, L., Wang, D., Huang, Q., Mo, Y., & Xie, G. (2016). Gene structures, evolution and transcriptional profiling of the WRKY gene family in castor bean (Ricinus communis L.). *PLoS One, 11*(2), e0148243.

[26] Götz, S., García-Gómez, J. M., Terol, J., Williams, T. D., Nagaraj, S. H., Nueda, M. J., & Conesa, A. (2008). High-throughput functional annotation and data mining with the Blast2GO suite. *Nucleic acids research, 36*(10), 3420-3435.

[27] Baillo, E. H., Kimotho, R. N., Zhang, Z., & Xu, P. (2019). Transcription factors associated with abiotic and biotic stress tolerance and their potential for crops improvement. *Genes, 10*(10), 771.

[28] Yu, Y., Hu, R., Wang, H., Cao, Y., He, G., Fu, C., & Zhou, G. (2013). MlWRKY12, a novel Miscanthus transcription factor, participates in pith secondary cell wall formation and promotes flowering. *Plant science, 212*, 1-9.

[29] Bi, C., Xu, Y., Ye, Q., Yin, T., & Ye, N. (2016). Genome-wide identification and characterization of WRKY gene family in Salix suchowensis. *PeerJ, 4*, e2437.

[30] Ding, M., Chen, J., Jiang, Y., Lin, L., Cao, Y., Wang, M., & Ye, W. (2015). Genome-wide investigation and transcriptome analysis of the WRKY gene family in Gossypium. *Molecular Genetics and Genomics, 290*, 151-171.

[31] Li, D., Liu, P., Yu, J., Wang, L., Dossa, K., Zhang, Y., & Zhang, X. (2017). Genome-wide analysis of WRKY gene family in the sesame genome and identification of the WRKY genes involved in responses to abiotic stresses. *BMC plant biology, 17*(1), 1-19.

[32] Ross, C. A., Liu, Y., & Shen, Q. J. (2007). The WRKY gene family in rice (Oryza sativa). *Journal of Integrative Plant Biology, 49*(6), 827-842.

[33] Luo, X., Bai, X., Sun, X., Zhu, D., Liu, B., Ji, W., & Zhu, Y. (2013). Expression of wild soybean WRKY20 in Arabidopsis enhances drought tolerance and regulates ABA signalling. *Journal of experimental botany, 64*(8), 2155-2169.

[34] Zou, M., Guo, B., & He, S. (2011). The roles and evolutionary patterns of intronless genes in deuterostomes. *International Journal of Genomics*, 2011.

[35] Li, M. Y., Xu, Z. S., Tian, C., Huang, Y., Wang, F., & Xiong, A. S. (2016). Genomic identification of WRKY transcription factors in carrot (Daucus carota) and analysis of evolution and homologous groups for plants. *Scientific reports, 6*(1), 23101.

[36] Phukan, U. J., Jeena, G. S., & Shukla, R. K. (2016). WRKY transcription factors: molecular regulation and stress responses in plants. *Frontiers in plant science, 7*, 760.

[37] Tao, Z., Kou, Y., Liu, H., Li, X., Xiao, J., & Wang, S. (2011). OsWRKY45 alleles play different roles in abscisic acid signalling and salt stress tolerance but similar roles in drought and cold tolerance in rice. *Journal of experimental botany, 62*(14), 4863-4874.

[38] Hernandez-Garcia, C. M., & Finer, J. J. (2014). Identification and validation of promoters and cis-acting regulatory elements. *Plant Science, 217*, 109-119.

[39] Lin, J. R., & Hu, J. (2013). SeqNLS: nuclear localization signal prediction based on frequent pattern mining and linear motif scoring. *PloS one, 8*(10), e76864.

[40] Yenerall, P., & Zhou, L. (2012). Identifying the mechanisms of intron gain: progress and trends. *Biology direct, 7*(1), 1-10.

[41] Tao, Z., Liu, H., Qiu, D., Zhou, Y., Li, X., Xu, C., & Wang, S. (2009). A pair of allelic WRKY genes play opposite roles in rice-bacteria interactions. *Plant physiology, 151*(2), 936-948.

[42] Jin, Y., Ding, X., Li, J., & Guo, Z. (2022). Isolation and characterization of wheat ice recrystallisation inhibition gene promoter involved in low temperature and methyl jasmonate responses. *Physiology and Molecular Biology of Plants, 28*(11-12), 1969-1979.

[43] Lovering, R., Hanson, I. M., Borden, K. L., Martin, S., O'Reilly, N. J., Evan, G. I., & Freemont, P. S. (1993). Identification and preliminary characterization of a protein motif related to the zinc finger. *Proceedings of the National Academy of Sciences, 90*(6), 2112-2116.

[44] Li, J., Besseau, S., Törönen, P., Sipari, N., Kollist, H., Holm, L., & Palva, E. T. (2013). Defense‐related transcription factors WRKY 70 and WRKY 54 modulate osmotic stress tolerance by regulating stomatal aperture in A rabidopsis. *New Phytologist*, 200(2), 457-472.

[45] Jiang, Y., Liang, G., & Yu, D. (2012). Activated expression of WRKY57 confers drought tolerance in Arabidopsis. *Molecular plant*, 5(6), 1375-1388.

[46] Wang, C. T., Ru, J. N., Liu, Y. W., Yang, J. F., Li, M., Xu, Z. S., & Fu, J. D. (2018). The maize WRKY transcription factor ZmWRKY40 confers drought resistance in transgenic Arabidopsis. *International journal of molecular sciences*, 19(9), 2580.

[47] Srivastava, R., Kumar, S., Kobayashi, Y., Kusunoki, K., Tripathi, P., Kobayashi, Y., ... & Sahoo, L. (2018). Comparative genome-wide analysis of WRKY transcription factors in two Asian legume crops: Adzuki bean and Mung bean. *Scientific reports*, 8(1), 16971.

1. Corresponding Author: Yongkang Yan，2898859469@qq.com

   Fund projects: Overseas Scholar Program in the Hebei Province (C20190514), Science and Technology Project of Hebei Province (15457605D, 144576106D), National Natural Science Foundation of China (12072205) [↑](#footnote-ref-1)
