# Supplementary figures and images for: Genome-wide identification of WRKY transcription factor family members in Miscanthus sinensis (Miscanthus sinensis Anderss)
Source: Sci Rep. 2024 Mar 6;14:5522. doi: 10.1038/s41598-024-55849-1 (PMC10918066; doi:10.1038/s41598-024-55849-1)

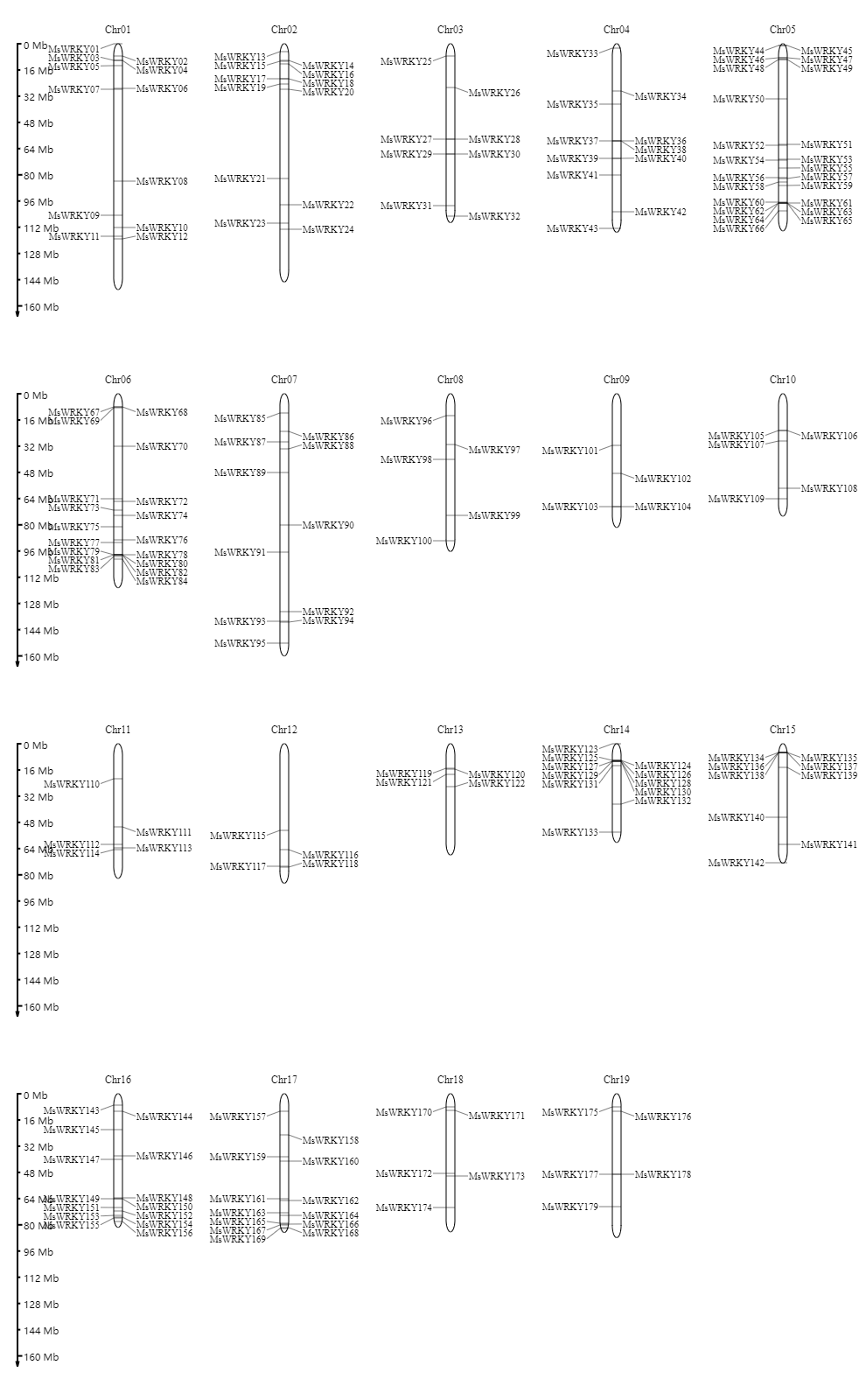

Supplement: Supplementary file 1 — Supplementary Information. [file 41598_2024_55849_MOESM1_ESM.zip › Chromosome mapping/染色体定位图.jpg]

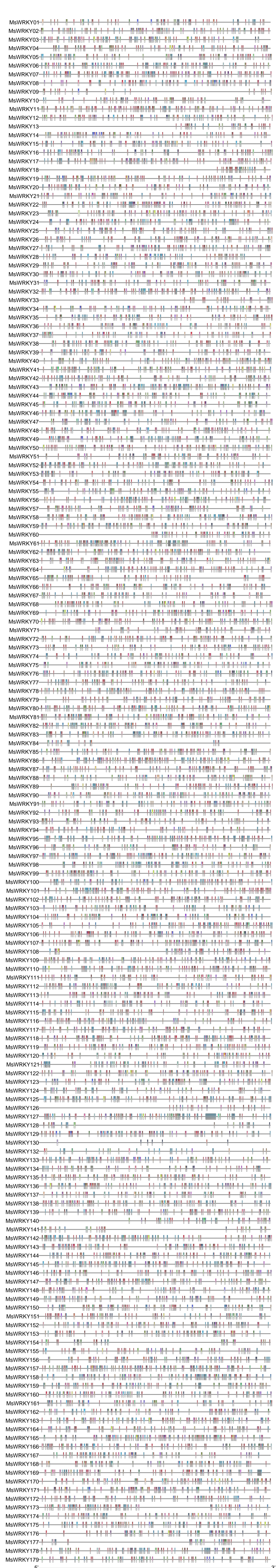

Supplement: Supplementary file 1 — Supplementary Information. [file 41598_2024_55849_MOESM1_ESM.zip › Cis-acting elements/顺式作用元件出图.pdf]

### Colored ranges

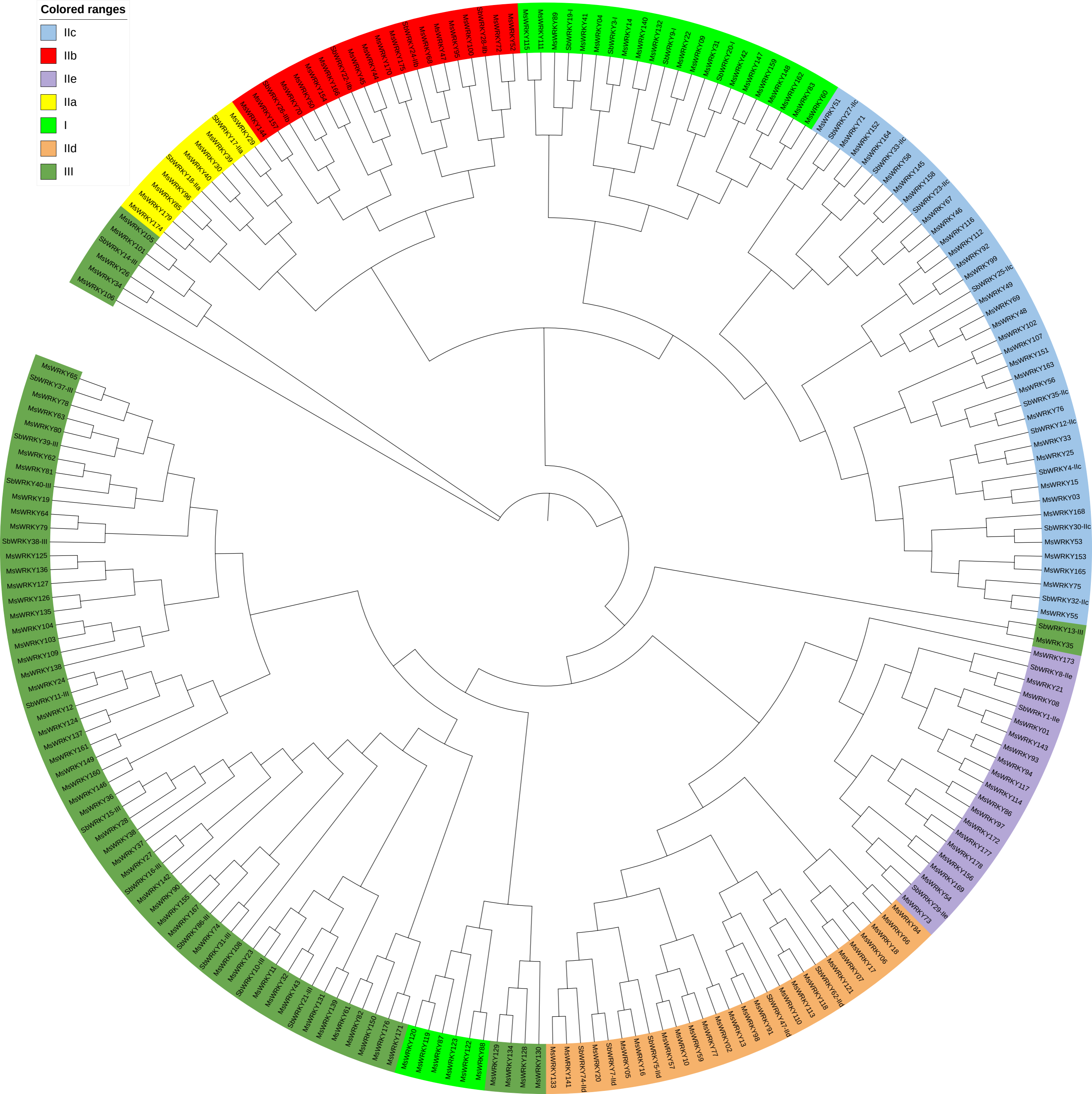

Supplement: Supplementary file 1 — Supplementary Information. [file 41598_2024_55849_MOESM1_ESM.zip › evolutionary tree/进化树出图.pdf]

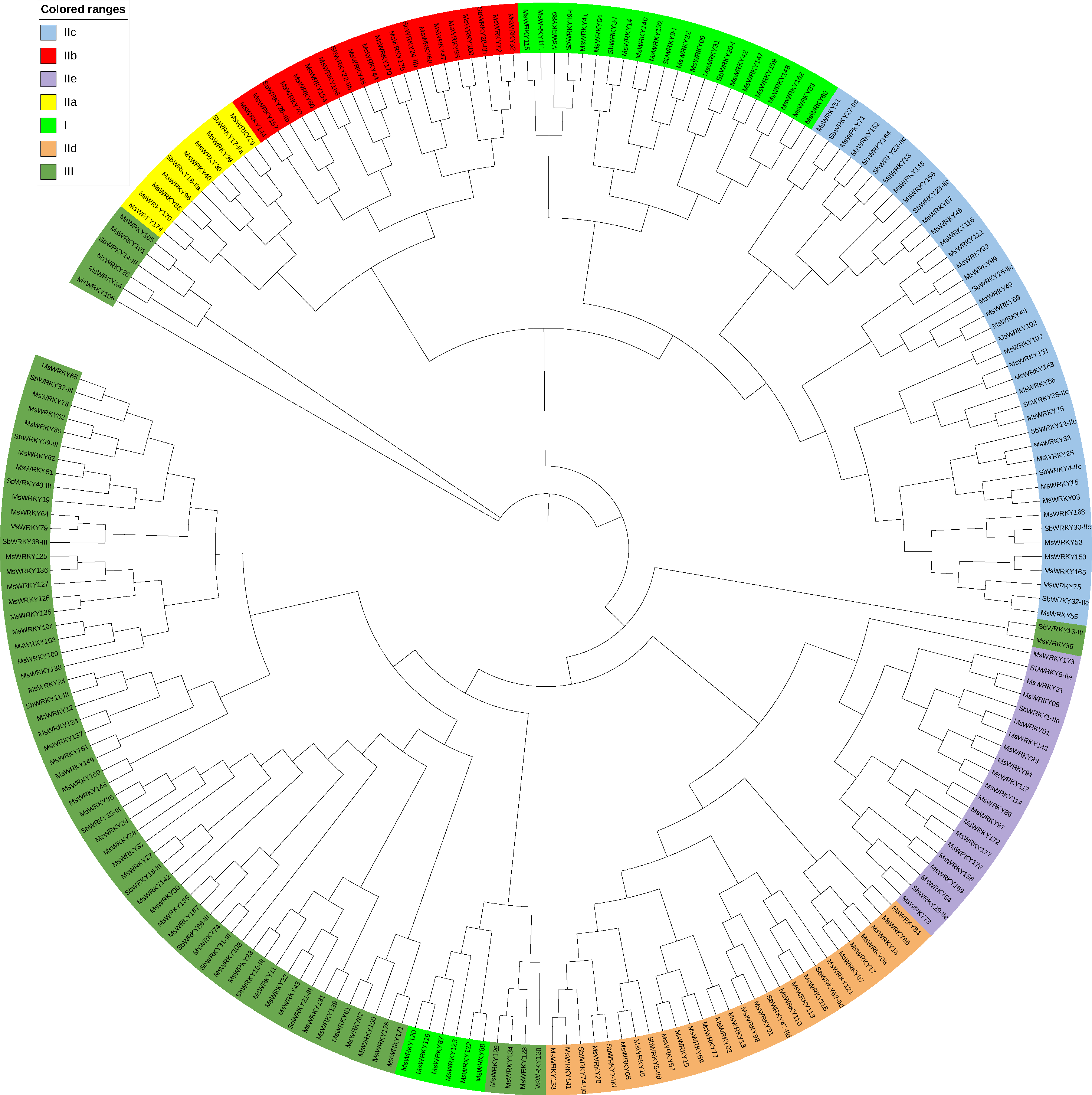

Supplement: Supplementary file 1 — Supplementary Information. [file 41598_2024_55849_MOESM1_ESM.zip › evolutionary tree/进化树出图.png]

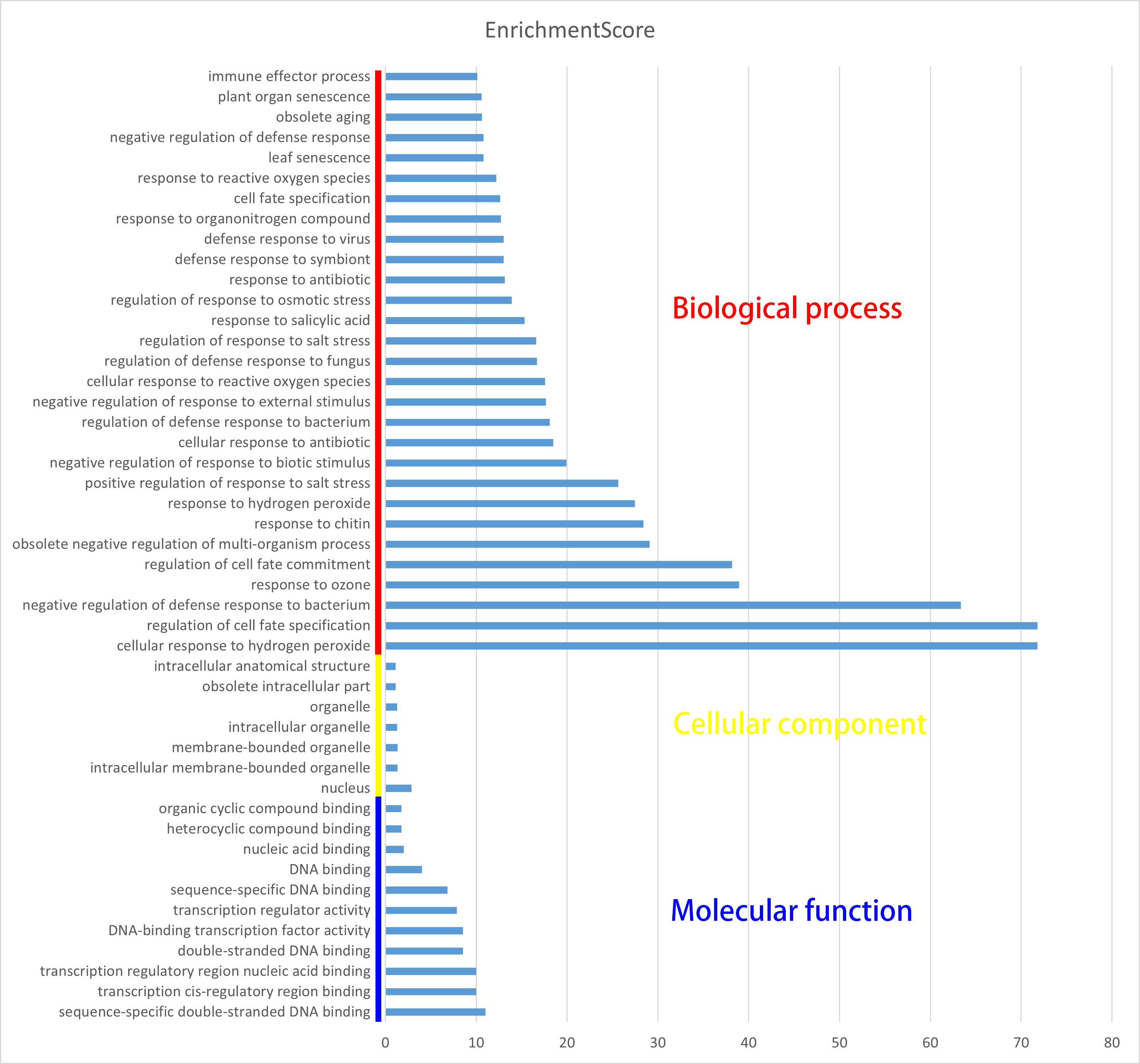

Supplement: Supplementary file 1 — Supplementary Information. [file 41598_2024_55849_MOESM1_ESM.zip › Gene ontology annotation/GO富集出图.png]

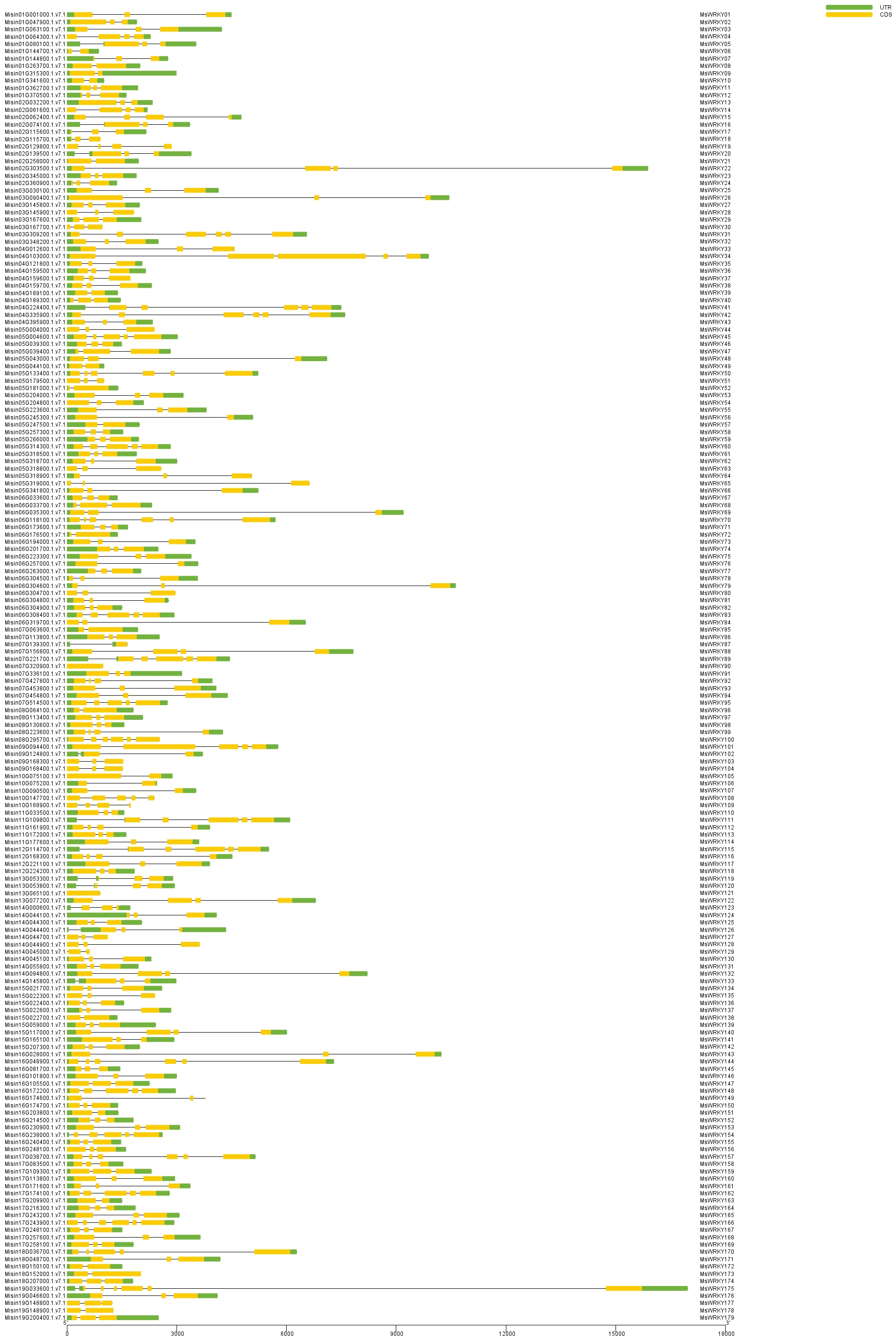

Supplement: Supplementary file 1 — Supplementary Information. [file 41598_2024_55849_MOESM1_ESM.zip › Gene structure analysis/基因结构分析.jpg]

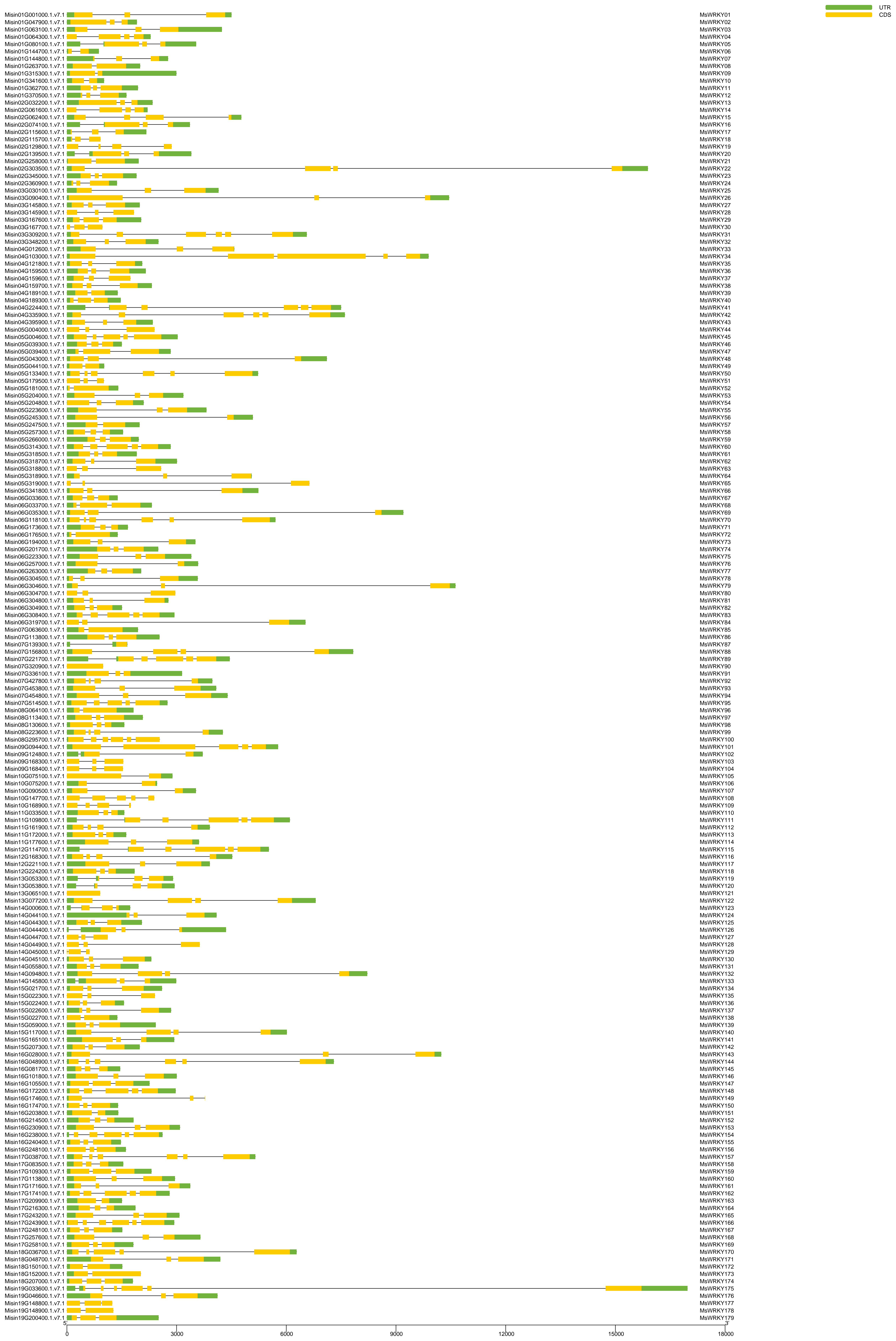

Supplement: Supplementary file 1 — Supplementary Information. [file 41598_2024_55849_MOESM1_ESM.zip › Gene structure analysis/基因结构分析.pdf]

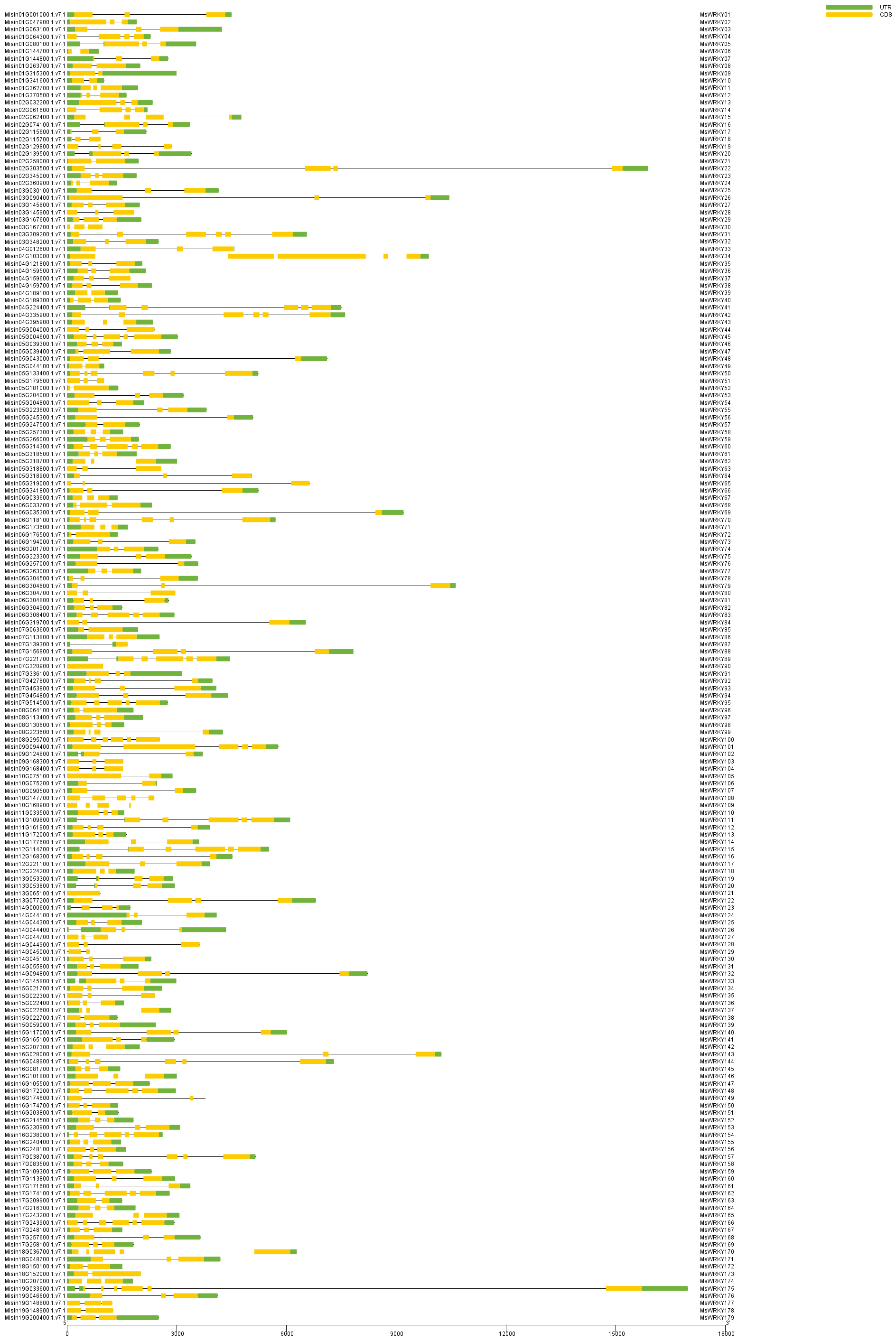

Supplement: Supplementary file 1 — Supplementary Information. [file 41598_2024_55849_MOESM1_ESM.zip › Gene structure analysis/基因结构分析.png]

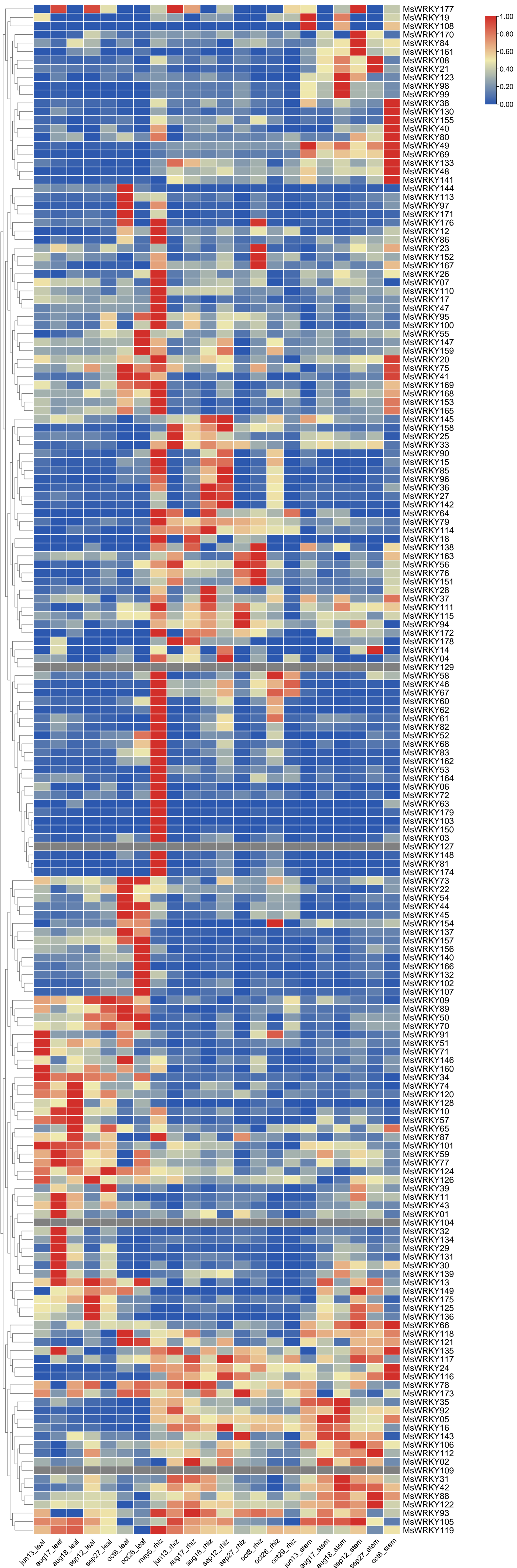

Supplement: Supplementary file 1 — Supplementary Information. [file 41598_2024_55849_MOESM1_ESM.zip › heatmap of the genes/热图出图.pdf]

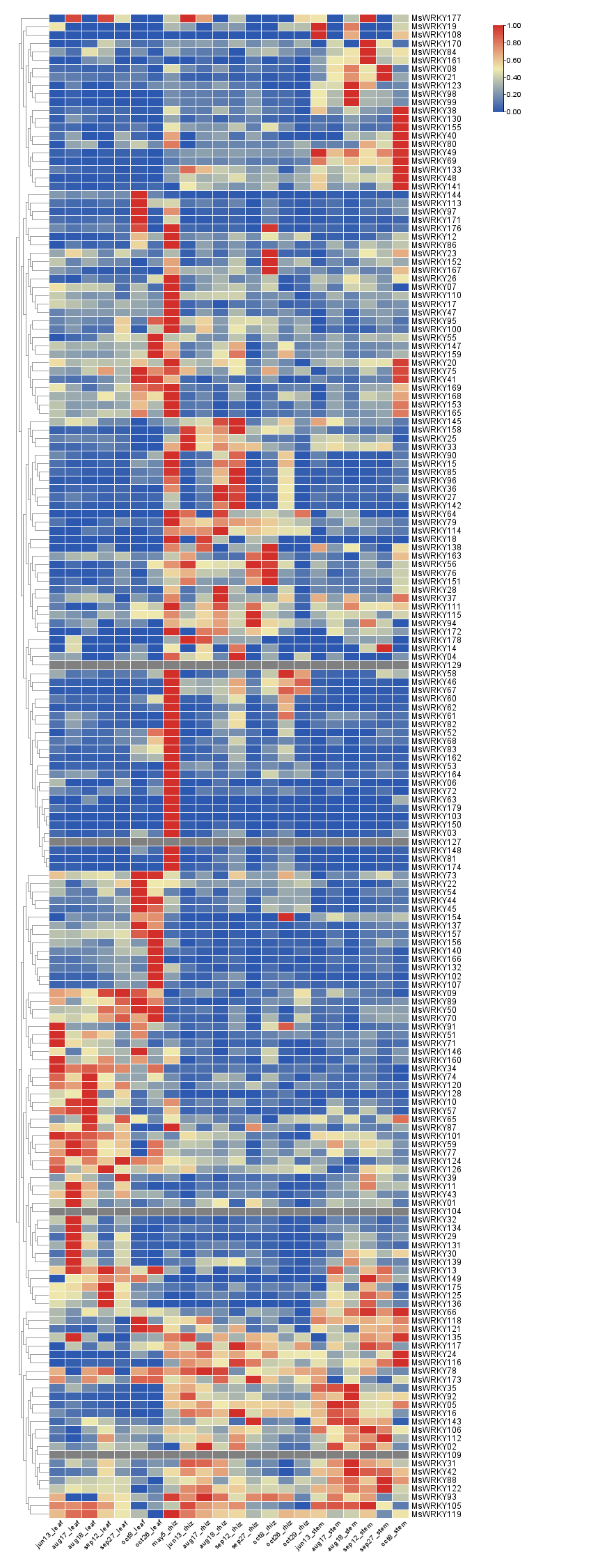

Supplement: Supplementary file 1 — Supplementary Information. [file 41598_2024_55849_MOESM1_ESM.zip › heatmap of the genes/热图出图.png]

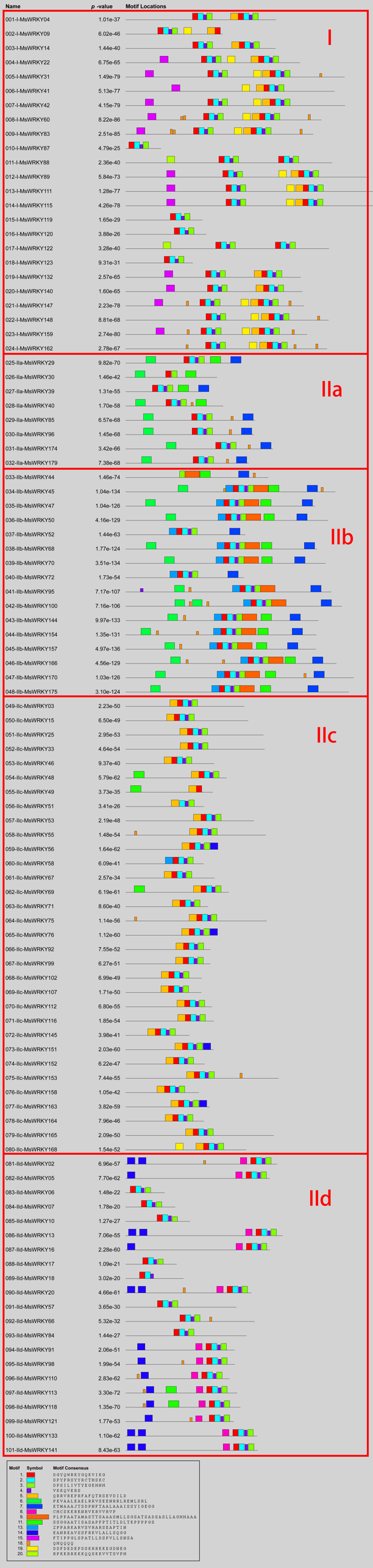

Supplement: Supplementary file 1 — Supplementary Information. [file 41598_2024_55849_MOESM1_ESM.zip › Motif-MEME analysis/motif出图I-IId.png]

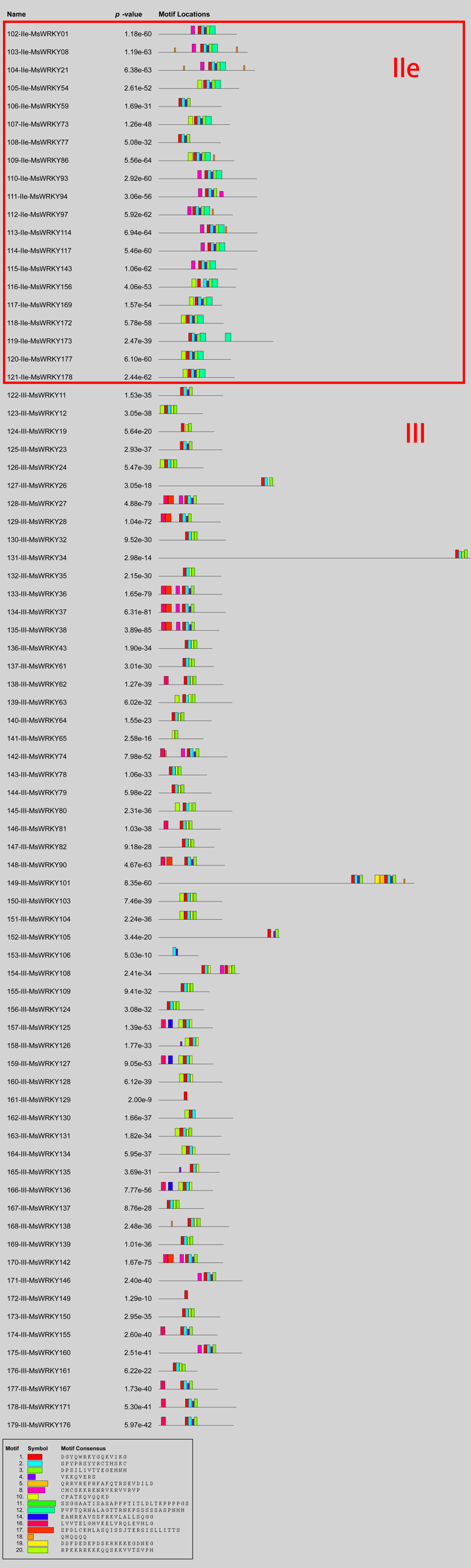

Supplement: Supplementary file 1 — Supplementary Information. [file 41598_2024_55849_MOESM1_ESM.zip › Motif-MEME analysis/motif出图IIe-III.png]

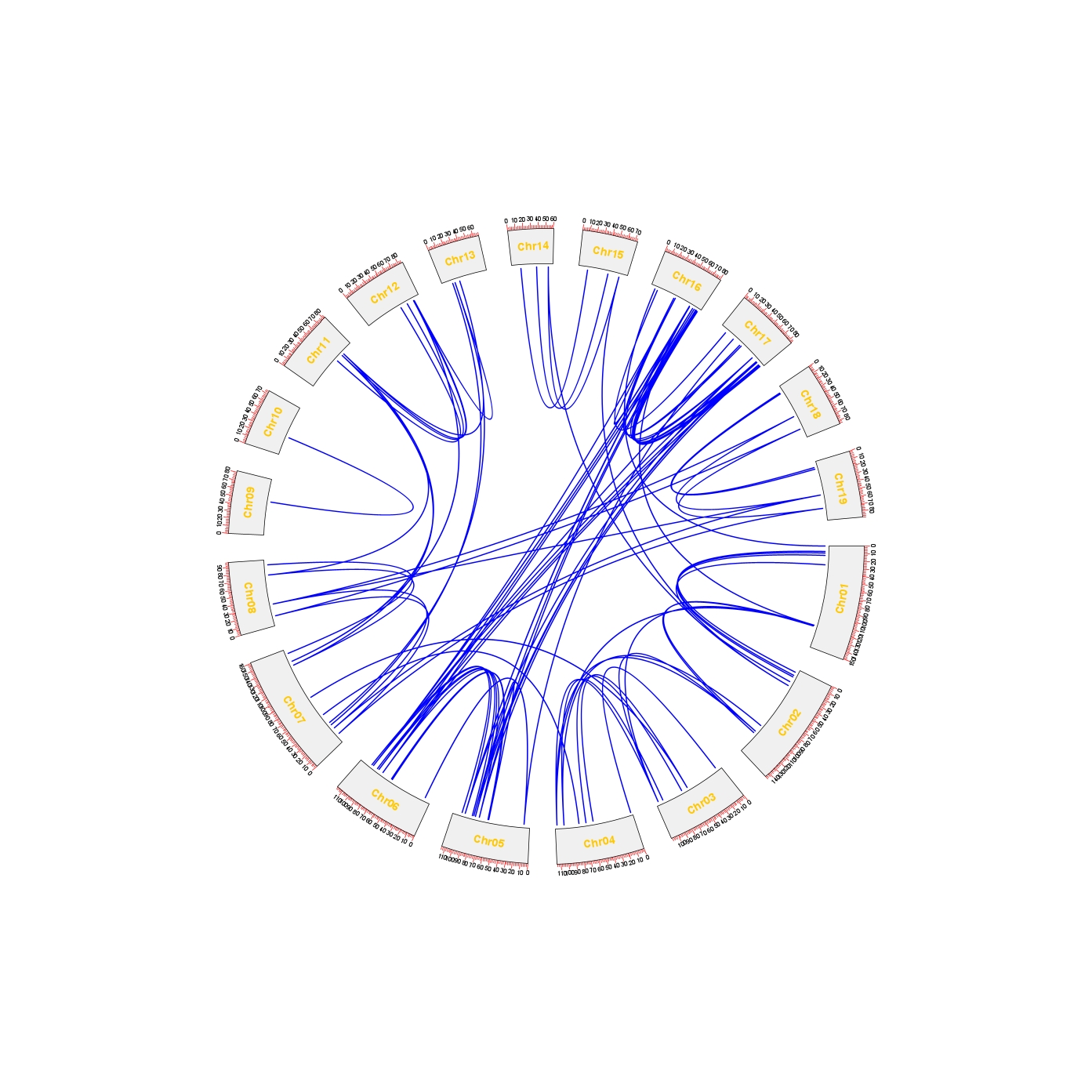

Supplement: Supplementary file 1 — Supplementary Information. [file 41598_2024_55849_MOESM1_ESM.zip › Synteny analysis/串联重复序列分析.jpg]

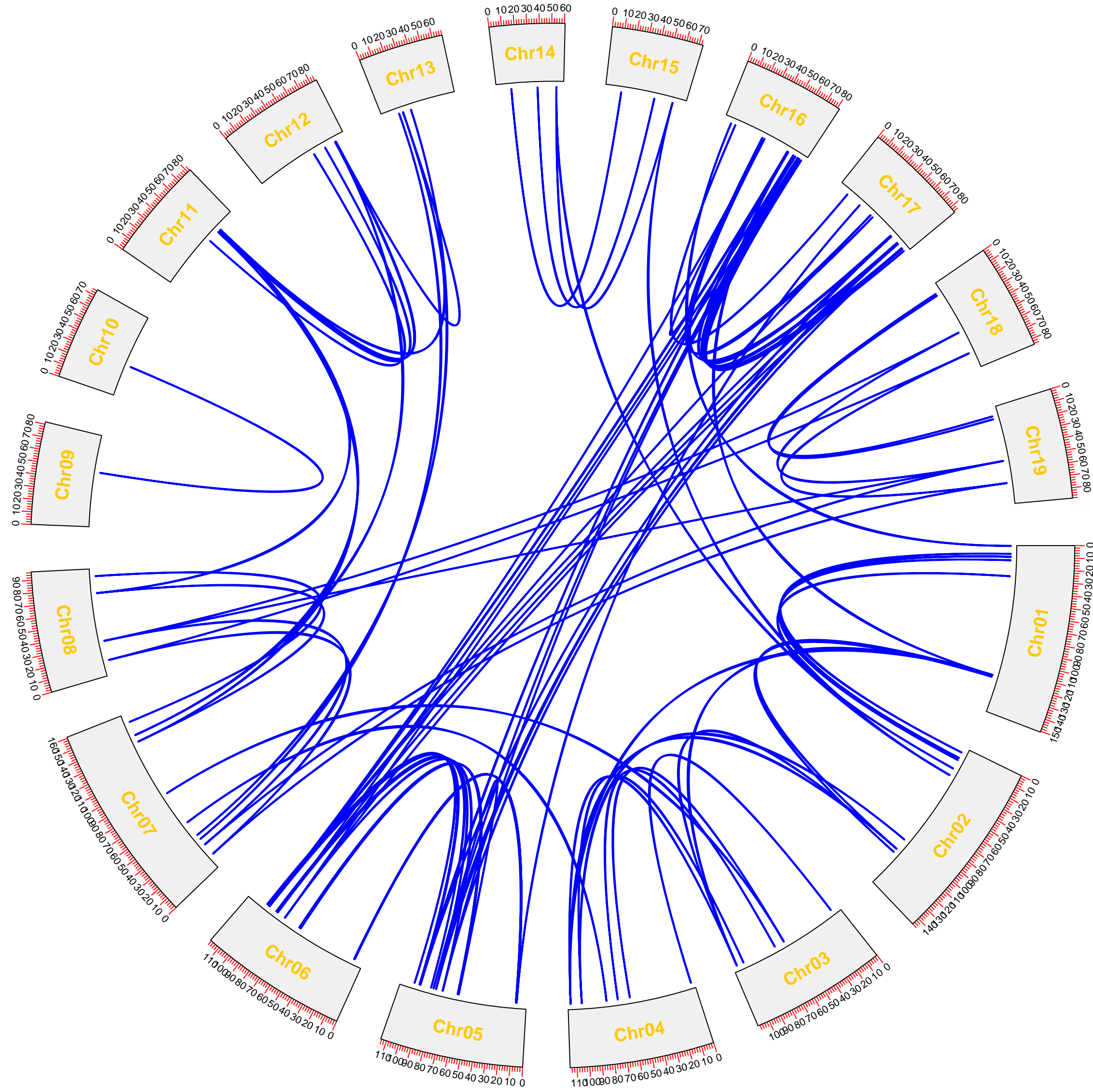

Supplement: Supplementary file 1 — Supplementary Information. [file 41598_2024_55849_MOESM1_ESM.zip › Synteny analysis/串联重复序列分析.pdf]

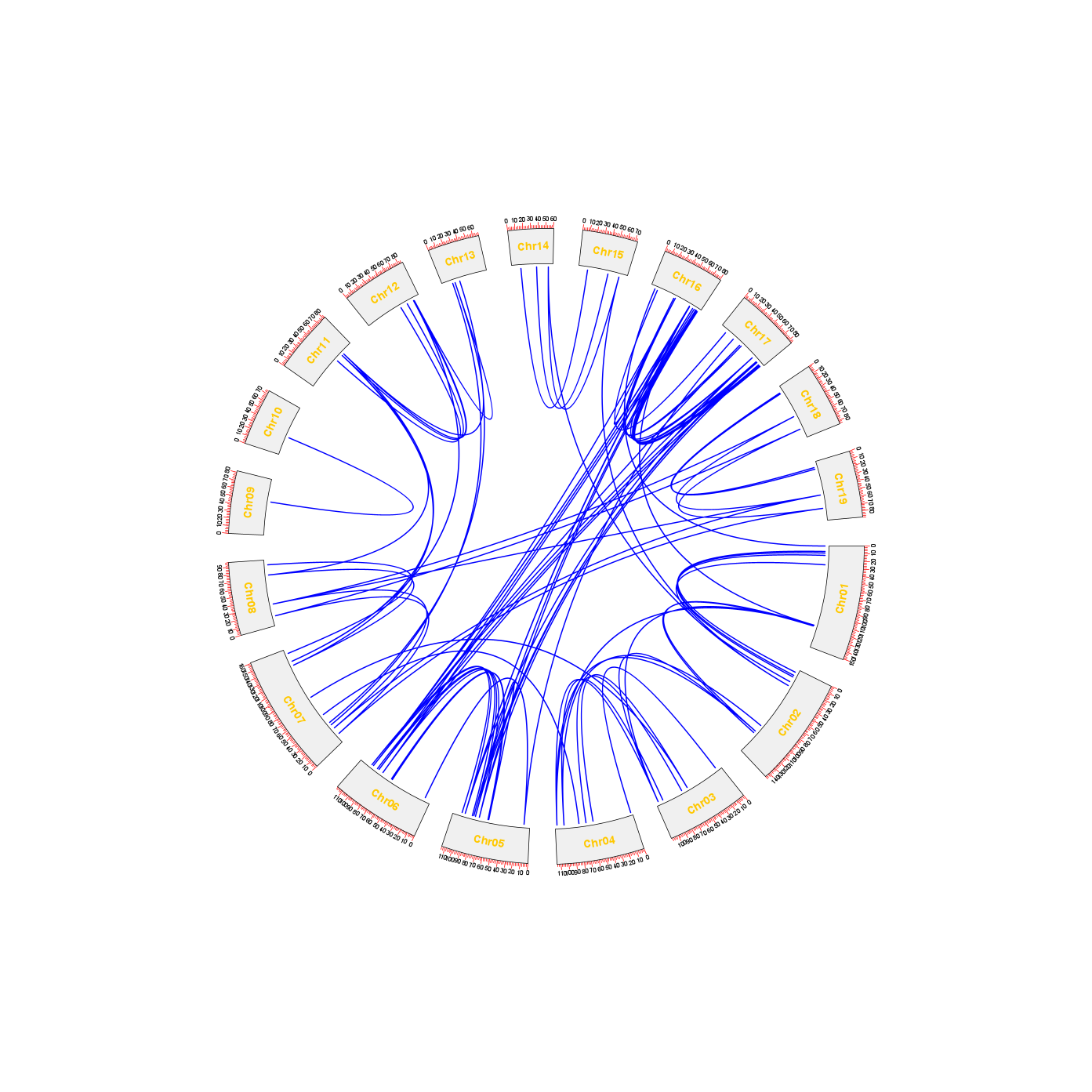

Supplement: Supplementary file 1 — Supplementary Information. [file 41598_2024_55849_MOESM1_ESM.zip › Synteny analysis/串联重复序列分析.png]

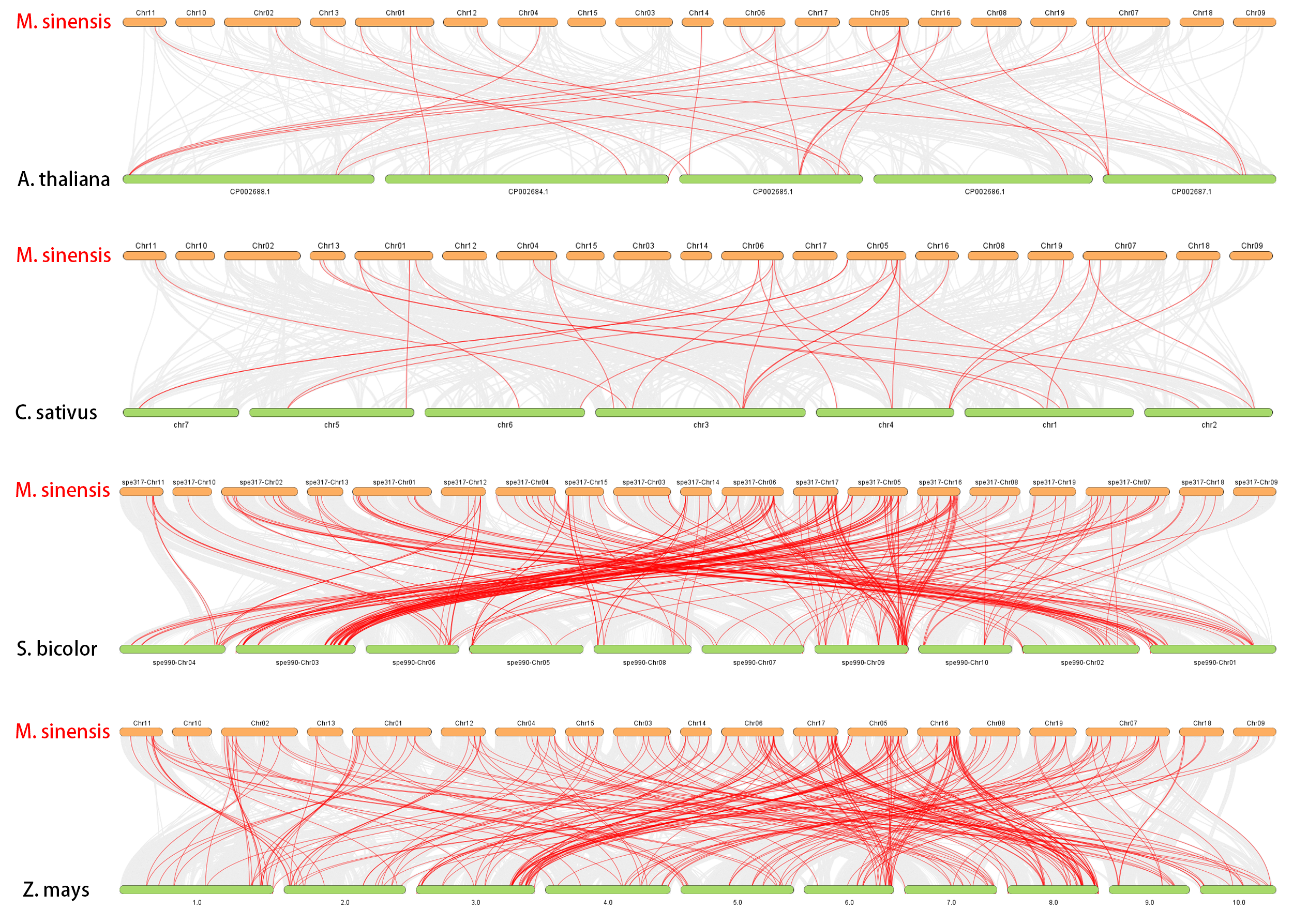

Supplement: Supplementary file 1 — Supplementary Information. [file 41598_2024_55849_MOESM1_ESM.zip › Synteny analysis/基因组比对.png]
